# The DNA-binding induced (de)AMPylation activity of a *Coxiella burnetii* Fic enzyme targets Histone H3

**DOI:** 10.1038/s42003-023-05494-7

**Published:** 2023-11-06

**Authors:** Dorothea Höpfner, Adam Cichy, Vivian Pogenberg, Christoph Krisp, Soraya Mezouar, Nina C. Bach, Jan Grotheer, Sandra Madariaga Zarza, Eric Martinez, Matteo Bonazzi, Matthias J. Feige, Stephan A. Sieber, Hartmut Schlüter, Aymelt Itzen

**Affiliations:** 1 Institute of Biochemistry and Signal Transduction, University Medical Center Hamburg-Eppendorf (UKE), Martinistraße 52, 20246 Hamburg, Germany; 2 Center for Integrated Protein Science Munich (CIPSM), Department Chemistry, Group of Proteinchemistry, Technical University of Munich, Lichtenbergstraße 4, 85747 Garching, Germany; 3 Institute of Clinical Chemistry and Laboratory Medicine, Section Mass Spectrometry and Proteomics, University Medical Center Hamburg-Eppendorf (UKE), Martinistraße 52, 20246 Hamburg, Germany; 4 Aix-Marseille University, Institut de Recherche pour la Développement (IRD), Assistance Publique-Hôpitaux de Marseille (APHM), Microbes Evolution Phylogeny and Infections (MEPHI), Institut Hospitalo-Universitaire (IHU)-Méditerranée Infection, Boulevard Jean Moulin, 13005 Marseille, France; 5 Technical University of Munich (TUM), TUM School of Natural Sciences, Department of Biosciences, Chair of Organic Chemistry II, Center for Functional Protein Assemblies (CPA), Ernst-Otto-Fischer Straße 8, 85748 Garching, Germany; 6 Cellular and Molecular Biology of Bacterial Infections, Institut de Recherche en Infectiologie de Montpellier (IRIM), Université de Montpellier, UMR 9004 - Centre national de la recherche scientifique (CNRS), 1919 Route de Mende, 34293 Montpellier, France; 7 Center for Functional Protein Assemblies (CPA), Department of Bioscience, TUM School of Natural Sciences, Technical University of Munich, Lichtenbergstraße 4, 85748 Garching, Germany; 8 Center for Structural Systems Biology (CSSB), University Medical Center Hamburg-Eppendorf (UKE), Martinistraße 52, 20246 Hamburg, Germany

**Keywords:** Nucleotide-binding proteins, X-ray crystallography, Enzyme mechanisms, Bacterial toxins

## Abstract

The intracellular bacterial pathogen *Coxiella burnetii* evades the host response by secreting effector proteins that aid in establishing a replication-friendly niche. Bacterial *filamentation induced by cyclic AMP* (Fic) enzymes can act as effectors by covalently modifying target proteins with the posttranslational AMPylation by transferring adenosine monophosphate (AMP) from adenosine triphosphate (ATP) to a hydroxyl-containing side chain. Here we identify the gene product of *C. burnetii CBU_0822*, termed *C. burnetii* Fic 2 (CbFic2), to AMPylate host cell histone H3 at serine 10 and serine 28. We show that CbFic2 acts as a bifunctional enzyme, both capable of AMPylation as well as deAMPylation, and is regulated by the binding of DNA via a C-terminal helix-turn-helix domain. We propose that CbFic2 performs AMPylation in its monomeric state, switching to a deAMPylating dimer upon DNA binding. This study unveils reversible histone modification by a specific enzyme of a pathogenic bacterium.

## Abstract (web summary)

The Fic2 enzyme of the pathogen *Coxiella burnetii* can act as a bifunctional histone modification enzyme, capable of AMPylation (as monomer) and deAMPylation (as dimer) upon DNA binding by a C-terminal helix-turn-helix domain.

## Introduction

AMPylation is a posttranslational modification (PTM) with implications for bacterial infection and protein homeostasis. Adenosine triphosphate (ATP) is used as a co-substrate by AMPylating enzymes to transfer the AMP moiety to hydroxyl-containing side chains, such as serine, threonine and tyrosine, of the target protein.

Filamentation-induced-by-cyclic-AMP (Fic) proteins represent a major class of AMP transferases that ubiquitously occur in all kingdoms of life^1^. They are characterized by the conserved Fic domain, which consists of six α-helices and a nine amino acid conserved Fic motif H_cat_xFx(D/E)(A/G)N(G/K)R as the active site^2,3^. The conserved histidine H_cat_ is indispensable for catalytic activity: it acts as a general base and deprotonates the hydroxyl-containing nucleophilic side chain of the target protein^4,5^. Mutations of H_cat_ to alanine thus render the enzyme inactive^6,7^.

Many representatives of Fic proteins are regulated by an inhibitory helix with conserved motif (S/T)xxxE_inh_(G/N). The motif is located either intermolecularly on an antitoxin partner protein (class I) or intramolecularly at the N- (class II) or C-terminal (class III) end of the Fic domain. By contacting the active site it inhibits the enzyme’s AMPylation activity^8^. In this process, the conserved glutamate E_inh_ projects with its negatively charged side chain into the phosphate binding region of the active pocket, where it inhibits AMPylation via a salt bridge with the arginine of the Fic motif, which normally orients the γ-phosphate of ATP^8,9^. Mutation of the conserved E_inh_ to glycine results in a constitutively active enzyme in most Fic proteins^6,10–12^. In many of the Fic proteins studied so far, increased enzymatic activity toward target proteins is accompanied by increased auto-AMPylation in vitro. Therefore, auto-AMPylation is generally accepted as a hallmark of enzymatic activity^9^.

Meanwhile, it has also been shown that some Fic proteins have the potential to reverse their own modification: e.g., the class II metazoan FICD and class II *Enterococcus faecalis* Fic protein (EfFic) can also act as deAMPylases^13–15^. FICD can deAMPylate its target protein, the ER chaperone BiP, both in vitro and in vivo via the same Fic domain that mediates AMPylation^13,14^.

The general mechanisms required to release the blockade by the inhibitory helix as well as switch from AMPylation to deAMPylation activity in class II Fic enzymes are not fully understood. However, one example where regulation is driven by dimerization is presented by the extensively studied bifunctional human FICD: The dimer interface is linked to the enzyme’s active site, so that dimerization transfers rigidity toward the E_inh_, preventing AMPylation and favoring deAMPylation. As a monomer, the E_inh_ has a greater conformational flexibility and ATP binds in an AMPylation-competent manner^16,17^. This ability to switch to deAMPylation upon dimerization is lost with the mutation of the E_inh_ of the inhibitory helix^13,14^, the reason why this mutant is lately referred to as deregulated and deAMPylation-defective instead of constitutively active^16^.

Besides FICD and its implications in protein homeostasis and stress response^18^, AMPylating enzymes occur in large numbers in bacterial pathogens. In particular, their occurrence in the class of Gammaproteobacteria suggests that Fic proteins play a critical role in pathogenicity toward their host cells^1^. So far, small GTPases have been shown to be the prevalent target of FIC enzymes during infection, such as VopS from *Vibrio parahaemolyticus*^7^, IbpA from *Histophilus somni*^6^, Bep1 from *Bartonella rochalimae*^19^ or AnkX from *Legionella pneumophila*^20^. The highly virulent pathogen *Coxiella burnetii* causes Q fever, an asymptomatic disease with acute or chronic symptoms^21,22^. *C. burnetii* is an obligate intracellular, Gram-negative bacterium and is taken up endocytotically by macrophages into the phagosome^23,24^. The emerging acidified phagolysosome activates the metabolism of *C. burnetii*^25,26^. As a consequence, the bacterium releases bacterial effector proteins into the host cell cytosol via its Type 4B secretion system (T4BSS), approximately 4 to 8 h after the onset of infection^27,28^. These bacterial effectors target processes such as apoptosis, transcriptional modulation, proteasomal degradation, and maintenance of *Coxiella*-containing vacuole integrity via manipulation of host proteins^29^. Bioinformatic analyses predict approximately 140 *Coxiella* effectors, whose targeting and function remain unknown in many cases^30^. With the complete sequencing of the *Coxiella* genome five conserved Fic proteins can be predicted on the basis of their Fic motif^3,31,32^. According to S4TE 2.0, a search algorithm for predicting type IV effector proteins, the three genetic loci corresponding to predicted Fic proteins *CBU_0372*, *CBU_0822*, and *CBU_2078* are predicted to produce secreted effectors in *C. burnetii* RSA 493^33^.

Here, we identify the protein product of *CBU_0822*, in the following referred to as *Coxiella burnetii* Fic enzyme 2 (CbFic2), as a DNA-binding protein and AMP-transferase that AMPylates Histone H3 at S10 and S28. Our data suggest that CbFic2 activity is stimulated by DNA binding, which causes a switch from AMPylation to deAMPylation by dimerization.

## Results

### The predicted domain structure of CbFic2 suggests DNA binding

**Fig. 1:**
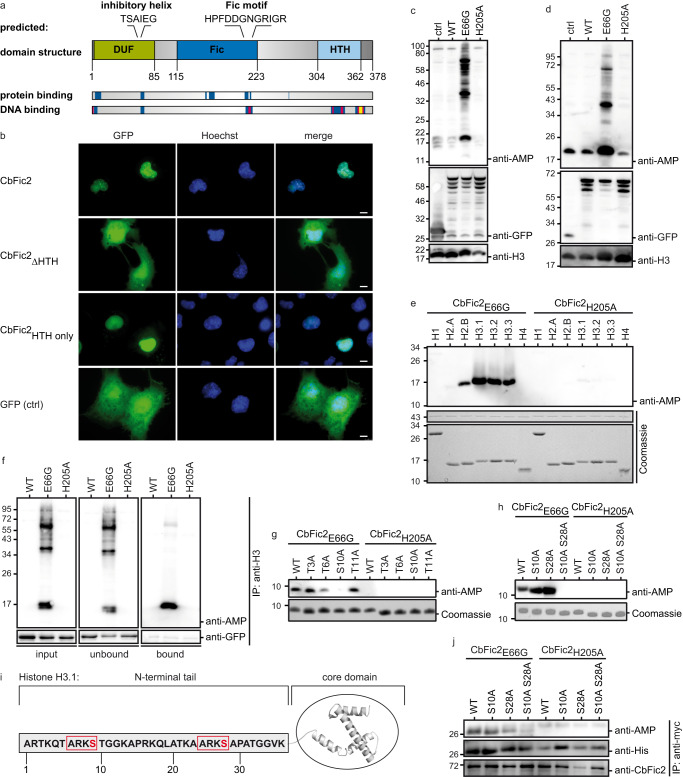
CbFic2 AMPylates Histone H3 in cellulo and in vitro. **a** Domain structure prediction and DNA and protein binding prediction of CbFic2 (*CBU_0822*). CbFic2 is a class II Fic protein consisting of 378 aa. According to SMART analysis, it has a conserved Fic domain (115–223 aa, blue) with the Fic motif HPFDDGNGRIGR (205–216 aa). The inhibitory helix with the sequence TSAIEG (62–67 aa) is located N-terminal to the Fic domain within the DUF4172 domain (4–85 aa, green) of unknown function. The C-terminus contains a helix-turn-helix (HTH) domain (304–362 aa, light blue)^34,100^. Protein- and DNA-binding regions are predicted with PredictProtein^36,101^. Protein binding (RI: 00-33) blue. DNA binding (RI: 00-33) blue, (RI: 34–66) magenta, (RI: 67–100) yellow. RI = reliability index, reliability of positive prediction. The scale of positive prediction ranges from 0 to 100. The higher the score, the more reliable the prediction. **b** Fluorescent microscopy analysis of protein localization after transient heterologous expression of GFP-CbFic2 full length (CbFic2) or without HTH domain (CbFic2_ΔHTH_) or the HTH domain alone (CbFic2_HTH only_) in Cos7 cells. GFP-fusion protein (green) was expressed for 24 h and cell nuclei were stained with Hoechst-33342 (blue). Images were taken by a Leica DMi8 wide field microscope using 100x magnification. Merge of images with GFP and DAPI filter, respectively, reveal co-localization of GFP-CbFic2 to the nucleus. Scale bars: 10 µm. See Supplementary Fig. S1a. **c** WB analysis of AMPylation patterns of whole cell lysates after transient heterologous expression of CbFic2-GFP or its mutants CbFic2_E66G_ and CbFic2_H205A_ in HEK293 cells. ctrl represents the expression of GFP alone. Fusion protein was expressed for 48 h in HEK293 cells. 20 μg of cleared RIPA lysate per lane were run on Bis-Tris gels and blotted on PVDF. Blots were probed with an anti-AMP antibody, stripped, cut into strips, and treated with antibodies against GFP and histone H3 as expression and loading controls, respectively. See Supplementary Fig. S1b. **d** WB analysis of AMPylation patterns in acid-soluble nuclear fraction, containing histones, after transient heterologous expression of CbFic2-GFP or its mutants CbFic2_E66G_ and CbFic2_H205A_ in HEK293 cells. ctrl represents the expression of GFP alone. Fusion protein was expressed for 48 h in HEK293 cells. Acid-soluble nuclear proteins were isolated using acid extraction. 10 μg of acid-soluble nuclear fraction per lane were run on Bis-Tris gels and blotted on PVDF. Blots were probed with an anti-AMP antibody, stripped, cut into strips, and treated with antibodies against GFP and histone H3 as expression and loading controls, respectively. See Supplementary Fig. S1c. **e** WB analysis of AMPylation of recombinant histones by CbFic2_E66G_ in vitro. 0.1 mg ml^−^^1^ histones were incubated with 0.2 µM CbFic2_E66G_ or CbFic2_H205A_ in the presence of ATP, MgCl_2_ and DNA at 23 °C for 20 h. 50 ng histones were run on Laemmli gels, blotted on PVDF and probed with an anti-AMP antibody. For loading controls, 1 µg of histones were run on Laemmli gels and stained with Coomassie. **f** WB analysis of AMPylation after immunoprecipitation against histone H3 on HEK293 lysates after transient heterologous expression of GFP-CbFic2 or its mutants CbFic2_E66G_ and CbFic2_H205A_. 50 μg of lysate after transient heterologous expression of GFP-CbFic2 were treated in 200 µl with 1 μg anti-H3 antibody and protein A/G magnetic beads. Bound proteins were eluted with 50 μl 1x Laemmli. 10 μl each of the input and unbound sample including 6x Laemmli buffer and 10 μl of the elution (bound) were run on Laemmli gels, blotted on PVDF and probed with an anti-AMP antibody, before being stripped and treated with an antibody against GFP. **g** WB analysis of AMPylation of the Twinstrep-tagged N-terminal 20 aa of Histone H3 (TS-H3_1-20aa_) and its mutants T3A, T6A, S10A and T11A by CbFic2_E66G_ or CbFic2_H205A_ in vitro. 1 mg ml^−1^ TS-H3_1-20aa_ were incubated with 1 µM CbFic2_E66G_ in the presence of ATP, MgCl_2_ and DNA at 30 °C for 20 h. 100 ng peptide were run on Tris-Tricine gels, blotted on PVDF and probed with an anti-AMP antibody. For loading controls, 1 µg of peptide was run on Tris-Tricine gels and stained with Coomassie. **h** WB analysis of AMPylation of the Twinstrep-tagged N-terminal 36 aa of Histone H3 (TS-H3_1-36aa_) and its mutants S10A, S28A and S10A S28A by CbFic2_E66G_ or CbFic2_H205A_ in vitro. 1 mg ml^−1^ TS-H3_1-36aa_ were incubated with 5 µM CbFic2_E66G_ in the presence of ATP, MgCl_2_ and DNA at 30 °C for 20 h. 100 ng peptide were run on Tris-Tricine gels, blotted on PVDF and probed with an anti-AMP antibody. For loading controls, 1 µg of peptide was run on Tris-Tricine gels and stained with Coomassie. **i** Representation of modification sites at S10 and S28 (red) within the ARKS motif (red frame) in N-terminal tail of Histone H3.1 by CbFic2 as determined by mutational approaches with WB analysis and MS/MS analysis (Fig. 1g, h; Supplementary Fig. S2a). **j** WB analysis of AMPylation after anti-myc immunoprecipitation against myc- and his-tagged histone H3.1 and its mutants S10A, S28A or S10A S28A, transiently co-expressed in HEK293 cells with either GFP-CbFic2_E66G_ or GFP-CbFic2_H205A_. 50 μg of acid-soluble nuclear proteins 48 h post-transfection were treated in 100 µl with 2 μg anti-myc antibody and protein A/G magnetic beads. Bound proteins were eluted with 50 μl 1x Laemmli buffer. 10 μl were run on Bis-Tris gels, blotted on PVDF and probed with an anti-AMP antibody. The blot was stripped, cut into strips, and reprobed with antibodies against CbFic2, and His as expression control of histone H3.1.

CbFic2 is a class II Fic protein of 378 amino acids (aa) with a predicted isoelectric point (pI) of 9.75^32^. According to sequence analysis using SMART including outlier homologs and PFAM domains, it encompasses a conserved Fic domain (115–223 aa, Fic motif HPFDDGNGRIGR 205–216 aa) with N-terminal inhibitory helix (TSAIEG, 62–67 aa), an N-terminal domain of unknown function (DUF4172 domain, 4–85 aa) and a C-terminal helix-turn-helix (HTH) domain (304–362 aa) of the DeoR family (Fig. 1a)^34^. HTH domains are a common component of transcription factors in all kingdoms of life and are well known for their ability to bind DNA. They consist of a variable motif of two α-helices joined by a turn, and a third α-helix stabilizing the motif. HTH domains often occur in combination with catalytic domains, where the HTH domain may serve in localization or substrate recognition^35^. Typically, HTH domains bind to DNA as a homo- or hetero-dimer. The DeoR family of HTH domains is part of a winged HTH (wHTH) domain superclass that also comprises Z-DNA-binding domains^35^.

Sequence analysis using PredictProtein suggests DNA binding within the HTH domain, the C-terminal part of the Fic domain as well as the very N-terminus of CbFic2^36^. No protein binding is predicted for the HTH domain (Fig. 1a).

### CbFic2 shows HTH domain-dependent nuclear localization and colocalizes with histones

In order to identify potential targets of AMPylation by CbFic2, we analyzed the subcellular localization of Green fluorescent protein (GFP) fusion constructs expressed heterologously in Cos7-cells using fluorescence microscopy (Fig. 1b). GFP-fused CbFic2, but not GFP alone localized exclusively to the cell nucleus demonstrated by fluorescence superimposition of GFP with the nucleus-staining dye Hoechst-33342. Constructs with truncations of the very C-terminus of CbFic2 (CbFic2_1-371aa_ and CbFic2_1-362aa_) still localized to the nucleus, but a deletion of the HTH motif (CbFic2_1-300aa_, in the following termed CbFic2_ΔHTH_) led to dispersion of the protein throughout the cell (Supplementary Fig. S1a, Fig. 1b). A GFP-fusion protein only containing the HTH motif (CbFic2_301-361aa_, in the following termed CbFic2_HTH only_) localizes to the nucleus, thus identifying the HTH domain being responsible for the nuclear localization of CbFic2 (Fig. 1b).

### CbFic2 shows AMPylation in HEK293 cells

In order to identify potential targets of CbFic2, we first heterologously expressed CbFic2 as N- and C-terminal fusion constructs with GFP in HEK293 cells and performed western blotting (WB) with the anti-AMP antibody 17G6^37^ to detect AMP-modified proteins (Fig. 1c, Supplementary Fig. S1b). Despite their physiological irrelevance, Cos7 and HEK293 cells were chosen for ease in transient transfection, as the physiologically more relevant macrophages are notoriously difficult to transiently transfect. In keeping with previous studies of Fic-proteins, the E66G mutation was introduced into CbFic2 to create a deregulated, constitutively AMPylation-active enzyme^8^, whereas the CbFic2_H205A_ mutant served as inactive control^6,7^. The wild type (WT) protein was not expected to show AMPylation as the E66 at the inhibitory α-helix obstructs AMPylation-competent ATP binding^8^. Only the AMPylation-active CbFic2_E66G_ protein but none of the other mutants led to detectable AMPylation with distinct bands at an approximate molecular weight of MW = 20, 40 and 70 kDa as well as an increased background within the whole range of molecular weight (Fig. 1c, Supplementary Fig. S1b).

Since CbFic2 localizes to the nucleus, we speculated that CbFic2 might target histones, which have a similar molecular weight compared to the AMPylation band around MW = 20 kDa that was observed in CbFic2_E66G_ overexpressing cells (Fig. 1c). A WB against GFP and AMPylation after acid extraction of nuclear proteins, including histones, where cells were lysed with 0.5% Triton X-100, nuclei were separated by centrifugation and acid-soluble proteins including histones were extracted with 0.2 N HCl, showed the same MW = 20 kDa AMPylation band after CbFic2_E66G_ overexpression as in whole cell lysate analyses (Fig. 1d, Supplementary Fig. S1c). In N-terminally labeled CbFic2 constructs (GFP-CbFic2), this AMPylation band was dependent on the presence of the HTH domain; deletion of the last ß-sheet of the wing part of the HTH domain (CbFic2_E66G 1-362aa_) or the complete HTH domain (CbFic2_E66G ΔHTH_) resulted in loss of the histone associated AMPylation band, while the deletion of the very C-terminal 7 amino acids maintained AMPylation (CbFic2_E66G 1-371aa_) (Supplementary Fig. S1e, g). In C-terminally labeled CbFic2 constructs (CbFic2-GFP), the AMPylation band at MW = 20 kDa was not affected at all by the loss of the HTH domain (Supplementary Fig. S1d, f). Truncating the HTH domain by deleting the last ß-sheet (CbFic2_E66G 1-362aa_) did not interfere with nuclear localization, hinting at a functional role of the HTH domain beyond localization (Supplementary Fig. S1a, f, g).

### CbFic2 AMPylates Histone H3 at Serine 10 and Serine 28

Since previous results indicated that histones may be targeted by CbFic2, we tested their AMPylation in vitro. Indeed, after incubation of recombinant CbFic2 and histones in the presence of ATP, WB analyses with an anti-AMP antibody revealed that all Histone H3 variants as well as H2B and to a lesser extent H2A are AMPylated by CbFic2_E66G_ but not by catalytically inactive CbFic2_H205A_ (Fig. 1e). An anti-AMP WB after immunoprecipitation (IP) of Histone H3 from HEK293 cells heterologously overexpressing CbFic2 showed a distinct AMPylation signal at the appropriate molecular weight of around 17 kDa for CbFic2_E66G_ but not for CbFic2_H205A_, thus confirming that Histone H3 is also AMPylated by CbFic2_E66G_ in cellulo (Fig. 1f). Since the anti-Histone H3 antibody used for IP was generated using a part of the conserved Histone H3 core (aa 100 to the C-terminus) as immunogen, it cannot differentiate between Histone H3 variants.

Histone H3 was previously reported to be a target of the metazoan FIC protein^12,38,39^, but studies could only show AMPylation on recombinant histones in vitro or in spiked cell lysates, and—while tyrosine was ruled out for modification^39^—AMPylation sites were not identified.

Epigenetic control of histones is achieved by numerous PTMs at their N-terminal sequences. We hence hypothesized that the AMP-modification of Histone H3 by CbFic2 may also take place within the N-terminal tail. We purified recombinant N-terminal H3-peptides N-terminally fused to a Twinstrep tag (TS) from bacterial expression and tested for AMPylation by CbFic2_E66G_ in vitro by WB with an anti-AMP antibody (Fig. 1g, h). The WB demonstrates that the first 20 amino acids of histone H3 (H3_1-20aa_) are indeed enough to achieve AMPylation by CbFic2_E66G_ in vitro (Fig. 1g). A mutational approach revealed that the AMPylation was dependent on the presence of S10 within TS-H3_1-20aa_, since the TS-H3_1-20aa T3A_, TS-H3_1-20aa T6A_, and TS-H3_1-20aa T11A_ mutants, but not the TS-H3_1-20aa S10A_ mutant were AMPylated by CbFic2_E66G_ (Fig. 1g). Reversed phase nano-HPLC in combination with tandem mass spectrometry (MS/MS) of AMPylated H3_1-20aa_ peptide confirmed S10 as the site of histone H3 modification with a localization probability of more than 99% (Supplementary Fig. S2a). Therefore, the MS/MS data are supporting the in vitro AMPylation study and confirm histone H3 S10 as a target for AMPylation by CbFic2_E66G_.

The _7_ARKS_10_ motif modified at S10 in histone H3 is present a second time around S28 (_25_ARKS_28_) (Fig. 1i). We therefore subjected recombinant TS-H3_1-36aa_ to enzymatic AMPylation by CbFic2_E66G_ and confirmed successful modification by anti-AMP WB (Fig. 1h). Mutation of neither S10 nor S28 alone abrogated the anti-AMP signal, but the double mutant TS-H3_1-36aa S10A S28A_ could no longer be modified, suggesting that both S10 and S28, which are conserved among all Histone H3 variants, are targeted by CbFic2 (Fig. 1h, i).

To confirm that these sites are also modified in cellulo, human histone H3.1 and its point mutations S10A, S28A and S10A S28A in fusion with a C-terminal myc- and His_6_-tag, were co-expressed with either CbFic2_E66G_ or CbFic2_H205A_ fused to GFP, respectively, in HEK293 cells. Anti-AMP WB analysis of the bound IP fraction with an anti-myc antibody shows that the double mutation S10A S28A reduces the AMPylation signal of histone H3.1 by CbFic2_E66G_. S10 and S28 therefore seem to be also targeted in a cellular environment, although further modification sites cannot be excluded.

### CbFic2 shows distinct but slow AMPylation in macrophages

In humans, *Coxiella* primarily infects macrophages^24^. In order to confirm Histone H3 AMPylation by CbFic2 in the physiologically more relevant macrophages and identify additional targets, a stable, doxycycline-inducible cell line of N-terminally V5-tagged CbFic2 (V5-CbFic2) was established in the human monocytic cell line THP-1. Before induction of CbFic2 expression by doxycycline, THP-1 cells were then differentiated into monocyte-derived macrophages (MDMs) using phorbol-12-myristate-13-acetate (PMA)^40^ when indicated.

**Fig. 2:**
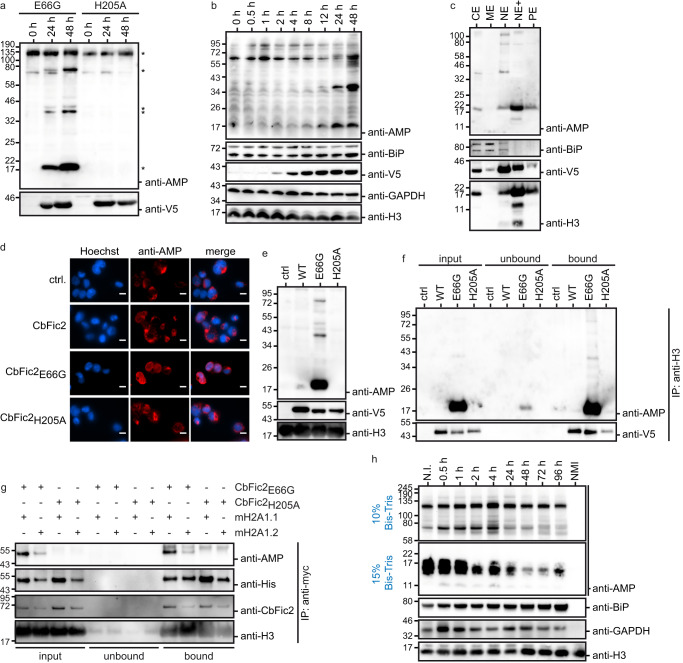
CbFic2 AMPylates Core Histone Macro-H2A.1. **a** WB analysis of AMPylation pattern in THP-1 MDMs before and after induction of V5-CbFic2_E66G_ and V5-CbFic2_H205A_ expression for 24 h and 48 h. The respective stable THP-1 cell lines V5-CbFic2_E66G_ and V5-CbFic2_H205A_ were differentiated into macrophages with PMA for 48 h before inducing the expression of CbFic2 with doxycycline. 20 μg RIPA lysate per lane were run on Bis-Tris gels and blotted on PVDF. The blot was probed with an anti-AMP antibody, stripped, and treated with an antibody against V5 tag. Clearly altered AMPylation bands are marked with an asterisk. **b** WB analysis of time-resolved AMPylation in THP-1 cells up until 48 h after induction of V5-CbFic2_E66G_ expression by doxycycline. The stable THP-1 cell line V5-CbFic2_E66G_ was induced by doxycycline and samples taken at the indicated time points. 20 μg RIPA lysate per lane was run on Bis-Tris gels and blotted on PVDF. The blot was probed with an anti-AMP antibody, stripped, and treated with antibodies against BiP, GAPDH, and Histone H3 as loading controls, and V5 as expression control of CbFic2, respectively. **c** WB analysis of AMPylation pattern in fractionated THP-1 MDMs after induction of V5-CbFic2_E66G_ expression for 48 h. The stable THP-1 cell line V5-CbFic2_E66G_ was differentiated into macrophages with PMA for 48 h before inducing the expression of CbFic2 with doxycycline. Cells were fractionated into cytoplasmic (CE), membrane (ME), nuclear soluble (NE), chromatin-bound (NE+) and cytoskeletal protein (PE) extracts using a subcellular protein fractionation kit for cultured cells. 5 μg per fraction were run on Bis-Tris gels and blotted on PVDF. The blot was probed with an anti-AMP antibody, stripped, cut into strips and treated with antibodies against BiP and Histone H3 as loading and fractionation controls, and V5 as expression and fractionation control of CbFic2, respectively. **d** Immunofluorescence analysis of AMPylation after 48 h of CbFic2 expression in macrophages using anti-AMP antibody. The respective stable THP-1 cell lines CbFic2, CbFic2_E66G_, CbFic2_H205A_, and the control cell line (ctrl.) were differentiated into macrophages for 48 h with PMA before inducing the expression of CbFic2 for 48 h using doxycycline. Cells were fixed and permeabilized. Cell nuclei were stained with Hoechst-33342 (blue), and AMPylation was visualized with antibody 17G6 (red). Scale bars: 10 µm. **e** WB analysis of AMPylation patterns in acid-soluble nuclear fraction, containing histones, after stable expression of V5-CbFic2 or its mutants V5-CbFic2_E66G_ and V5-CbFic2_H205A_ in THP-1 MDMs. ctrl represents the expression of the empty backbone alone. Tagged protein was expressed for 48 h in differentiated THP-1 cells. Acid-soluble nuclear proteins were isolated using acid extraction. 10 μg of acid-soluble nuclear fraction per lane were run on Bis-Tris gels and blotted on PVDF. Blots were probed with an anti-AMP antibody, stripped, cut into strips, and treated with antibodies against V5 and histone H3 as expression and loading controls, respectively. **f** WB analysis of AMPylation after immunoprecipitation against histone H3 from THP-1 MDMs acid-soluble nuclear fraction after stable expression of V5-CbFic2 or its mutants V5-CbFic2_E66G_ and V5-CbFic2_H205A_. 50 μg of acid-soluble nuclear fraction after 48 h of stable expression of V5-CbFic2 were treated in 200 µl with 1 μg anti-H3 antibody and protein A/G magnetic beads. Bound proteins were eluted with 50 μl 1x Laemmli. 10 μl each of the input and unbound sample including 6x Laemmli buffer and 10 μl of the elution (bound) were run on Laemmli gels, blotted on PVDF and probed with an anti-AMP antibody, before being stripped and treated with an antibody against V5. **g** WB analysis of AMPylation after anti-myc immunoprecipitation against myc- and his-tagged Core Histone Macro-H2A.1 isoforms mH2A1.1 or mH2A1.2, both transiently co-expressed in HEK293 cells with either GFP-CbFic2_E66G_ or CbFic2_H205A_. 50 μg of acid-soluble nuclear proteins 48 h post-transfection were treated in 100 µl with 2 μg anti-myc antibody and protein A/G magnetic beads. Bound proteins were eluted with 50 μl 1x Laemmli buffer. 10 μl each of the input and unbound sample including 6x Laemmli buffer and 10 μl of the elution (bound) were run on Bis-Tris gels, blotted on PVDF and probed with an anti-AMP antibody. The blot was stripped, cut into strips, and reprobed with antibodies against Histone H3 as loading control, V5 as expression control of CbFic2, and His as expression control of mH2A1, respectively. **h** WB analysis of AMPylation patterns over the time course of infection of murine J774 macrophages by virulent NMI *C. burnetii*. J774 cells were infected with *C. burnetii*, and at indicated time points lysed by RIPA. 20 μg of lysate per lane was run on Bis-Tris gels (gel percentages indicated to the left; for a full presentation of blots see Supplementary Fig. S2b) and blotted on PVDF. Blots were probed with an anti-AMP antibody, stripped, cut into strips, and treated with antibodies against BiP, GAPDH, and histone H3 as loading controls. N.I.: not infected, NMI: NMI cells alone.

In an anti-AMP WB analysis of whole cell lysates of V5-CbFic2_E66G_ and V5-CbFic2_H205A_ THP-1 MDMs taken 0, 24, and 48 h after induction of CbFic2 expression, an AMPylation pattern with 5 distinct bands at ca. 17, 40, 80, and above 135 kDa could be detected (Fig. 2a). WB analysis of the time course of AMPylation of doxycycline-induced THP-1 monocytes reveals that in cellulo AMPylation by CbFic2 is a slow process: While CbFic2 expression could be detected as early as 2 h after induction, AMPylation cannot be unambiguously detected before 24 h (Fig. 2b). Since the anti-AMP antibody can detect low levels of AMPylated proteins^37^, we conclude that the lack of AMP-signal in the early phase is a result of slow in cellulo AMPylation by CbFic2_E66G_ rather than a detection issue.

WB analysis of fractionation of THP-1 MDMs overexpressing V5-CbFic2_E66G_ for 48 h illustrates an accumulation of anti-V5 signal in the nuclear soluble (NE) and chromatin-bound (NE+) fraction, while the anti-AMP band of ca. 17 kDa is strongest in the chromatin-bound fraction (Fig. 2c). Immunofluorescence analysis of the AMPylation signal shows that upon overexpression of CbFic2_E66G_ but not CbFic2_H205A_ the anti-AMP signal relocates to the nucleus (Fig. 2d). WB analysis of the acid-soluble nuclear fraction of THP-1 MDMs overexpressing V5-CbFic2 for 48 h reveals co-fractionation of the anti-AMP signal at 17 kDa with the anti-V5 signal in the V5-CbFic2_E66G_ expressing sample (Fig. 2e), comparable to the results of Fig. 2c, thus confirming that the nuclear localization of CbFic2 observed in Cos7 cells holds true for the physiologically more relevant MDMs. Just as in HEK293 cells (Fig. 1f), an anti-AMP WB after IP of Histone H3 from THP-1 MDMs cells overexpressing V5-CbFic2 for 48 h showed a distinct AMPylation signal at the appropriate molecular weight of around 17 kDa for CbFic2_E66G_ but not for CbFic2_H205A_, thus confirming that Histone H3 is also AMPylated by CbFic2_E66G_ in the physiologically more relevant THP-1 MDMs (Fig. 2f). Furthermore, detection of CbFic2 by its V5-tag in the same experiment confirms the association of CbFic2 with histone H3 (Fig. 2f). While the association of GFP-CbFic2 with histone H3 after anti-H3 IP from HEK293 cells is only faint (Fig. 1f), the association of V5-tagged CbFic2 with histone H3 in THP-1 cells is more pronounced (Fig. 2f).

To determine the target proteins of AMPylation and the effects of CbFic2 activity in MDMs, immunoprecipitation of cell lysates with an anti-AMP antibody followed by protein identification via LC-MS/MS analysis was performed. The assays were based on biological triplicates of differentiated THP-1 lysates after doxycycline-induced CbFic2_E66G_ or CbFic2_H205A_ expression for 48 h, respectively (Fig. 2a). Based on a Student’s *t*-test (*p*-value < 0.05), 236 proteins were identified as significantly different between both sample sets (Data are available via ProteomeXchange with identifier PXD040330).

When looking at the list of proteins with the most significant enrichment in the CbFic2_E66G_ samples compared to CbFic2_H205A_ (LogFC >1.0; Supplementary Table 1), both Histone H3.3 and Core Histone Macro-H2A.1 (mH2A1) can be identified among others. Histone H3.3 is a non-canonical histone H3 variant prevalent in slow- or non-dividing cells that carries a serine at amino acid position 31 (S31) instead of an alanine and is clustered in euchromatin^41–44^. Identifying Histone H3.3 as AMPylation target by LC-MS/MS in MDMs confirms the validity of the previous identification of H3 AMPylation in HEK293 cells (Fig. 1f) and THP1 MDMs (Fig. 2f) by immunoprecipitation. mH2A1 consists of an H2A-like domain with over 60% sequence identity to H2A followed by a non-histone-related domain and represses transcription^45,46^. It also carries the AMPylation motif of CbFic2 in Histone H3, i.e., the peptide motif ARKS, at residues 155–158 aa^32^.

To validate mH2A1 as an in cellulo target for CbFic2, both isoforms of mH2A1 were expressed in fusion with a C-terminal myc- and His_6_-tag^47^ (MW 44.3 kDa), with co-expression of either CbFic2_E66G_ or CbFic2_H205A_ fused to GFP, respectively, in HEK293 cells. After IP with an anti-myc antibody and WB against AMP, an AMPylated band that overlaid with the anti-His band in the bound fraction of isoform mH2A1.1 in co-expression with GFP-CbFic2_E66G_ could be identified, while isoform mH2A1.2 showed only a weak signal (Fig. 2g). In co-expression with GFP-CbFic2_H205A_, this AMPylation band did not appear, confirming mH2A1.1 as a CbFic2 target in cellulo (Fig. 2g). AMPylation of mH2A.1 (MW 39 kDa) could therefore account for both the 40 kDa AMPylation band in WB analysis of whole cell lysates and acid-soluble nuclear fraction in HEK293 cells (Fig. 1c, d, f) as well as the 40 kDa band in THP-1 lysates (Fig. 2a).

Thus, both Histone H3 and mH2A1.1 are targets of AMPylation by CbFic2, both in HEK293 cells as well as the physiologically more relevant THP-1 MDMs.

### Changes in cellular AMPylation upon virulent *Coxiella* infection cannot be linked to CbFic2

To analyze patterns of AMPylation during infection by *C. burnetii*, the murine monocytic cell line J774 was infected with a multiplicity of infection (MOI) of 100 with virulent NMI *C. burnetii* for 96 h and analyzed for AMPylation by anti-AMP WB (Fig. 2h, Supplementary Fig. S2b). Three distinctive band regions were found in the molecular weight ranges of 17, 70 and 120 kDa. While the signal at 120 kDa remains largely stable over the infection period studied, the signal at 70 kDa first increases after infection before dropping between 4 h and 24 h after infection. The signal around 17 kDa decreases over time in the 15% gel (Fig. 2h, Supplementary Fig. S2b). A 40 kDa band cannot be observed (Supplementary Fig. S2b). Comparing AMPylation patterns during *Coxiella* infection with the pattern after CbFic2 expression in THP-1 macrophages, no obvious correlation of AMPylation patterns is discernible (Fig. 2b, h). Also, histone H3 AMPylation by CbFic2 at the approximate level of 17 kDa does not show obvious similarities with the change after NMI infection, where the AMPylation signal around 17 kDa decreases rather than increases over the time course. Unfortunately, the anti-AMP antibody cross-reacts with ADP ribosylation^37^. ADP ribosylation of target proteins on serine residues preceded by lysine residues, e.g., ADP ribosylation of S10 on Histone H3 within the ARKS motif, has been well described^48,49^. Aided by the Histone PARylation Factor-1 (HPF1), Poly (ADP-ribose) polymerases (PARPs) ADP ribosylate Histone H3 at S10 in response to DNA damage^50^. DNA fragmentation can be observed along with PARP activation after *Coxiella* infection in a similar setting and time span as our infection analysis (Fig. 2h)^51^. We speculate that any CbFic2-induced activity is overlaid by more prominent changes in ADP ribosylation, hampering AMPylation analysis during *Coxiella* infection. Therefore, we cannot contradict nor confirm that CbFic2 and histone AMPylation play a role in *Coxiella* infection up-to-date.

### CbFic2 is a dimer in the crystal structure

**Fig. 3:**
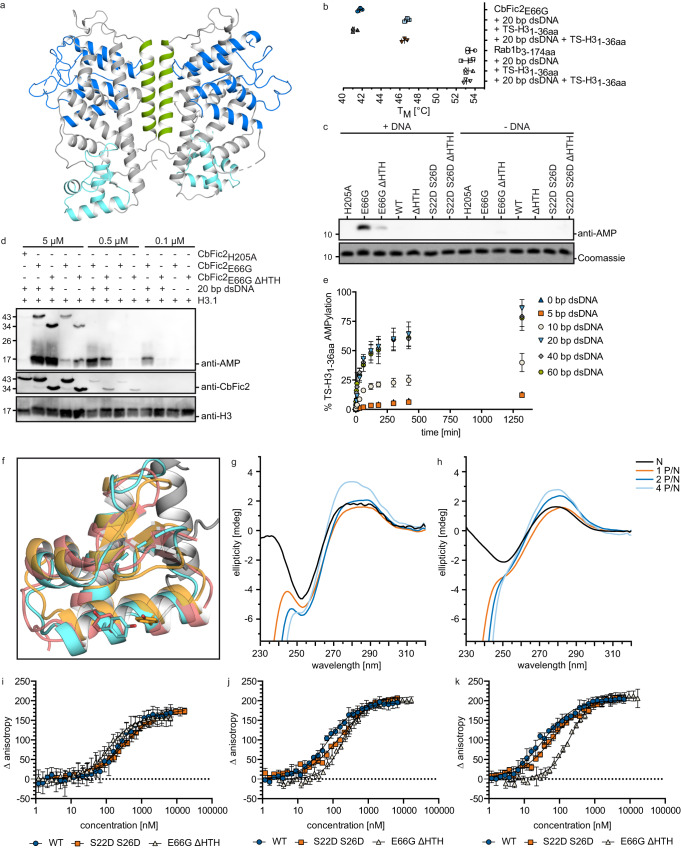
CbFic2 activity is stimulated by DNA binding. **a** Crystal structure of CbFic2 in the apo state. Dimer interface (17–41 aa) in green, Fic domain (96–223 aa) in blue, wHTH domain (300–367 aa) in light blue. For protein:protein contacts of the dimer interface, see Supplementary Fig. S3a. **b** Analysis of the thermostability of CbFic2 in the presence or absence of DNA by TSA. 4 µg (4 µM) TS-CbFic2_E66G_ or 2 µg Rab1b_3-174 aa_ (ctrl) in 20 mM HEPES pH 7.0, 50 mM NaCl, 1 mM MgCl_2_, 2 mM DTT supplemented with 5x SYPRO® Orange were measured in the presence or absence of 4 µM 20 bp dsDNA or 4 µM TS-H3_1 36aa_ as indicated. Samples were heated from 25–95 °C at a rate of 1 °C min^−1^ and fluorescence (ex. 465 nm, em. 590 nm) measured in a RT-PCR cycler. The melting temperature T_M_, as the inflection point of fluorescence increase during thermal protein unfolding, was determined at the zero point of the second derivative of each melting curve. Scatter plots represent technical triplicates as mean value with standard deviation as error bars using GraphPad Prism 8.0. **c** WB analysis of TS-H3_1-36aa_ AMPylation by CbFic2_H205A_, CbFic2_E66G_, CbFic2_E66G ΔHTH_, CbFic2, CbFic2_ΔHTH_, CbFic2_S22D S26D_ and CbFic2_S22D S26D ΔHTH_ in the absence or presence of DNA in vitro. 50 µM TS-H3_1-36aa_ were incubated with 1 µM of the indicated CbFic2 versions with or without 4 µM 20 bp dsDNA in the presence of 1 mM ATP and 1 mM MgCl_2_ at 37 °C for 8 h. 100 ng peptide were run on Tris-Tricine gels, blotted on PVDF and probed with an anti-AMP antibody. For loading controls, 1 µg of peptide was run on Tris-Tricine gels and stained with Coomassie. **d** WB analysis of full-length H3.1 AMPylation by CbFic2_H205A_, CbFic2_E66G_ and CbFic2_E66G ΔHTH_ in the absence or presence of DNA in vitro. 1 mg ml^−1^ H3.1 was incubated with 5 µM CbFic2_H205A_ or 5 µM, 0.5 µM or 0.1 µM CbFic2 CbFic2_E66G_ and CbFic2_E66G ΔHTH_ with or without 5 µM 20 bp dsDNA in the presence of 1 mM ATP and 1 mM MgCl_2_ at 23 °C oN. 50 ng H3.1 was run on 15% glycine gels, blotted on PVDF and probed with an anti-AMP antibody. For loading controls, the WB was stripped and reprobed with anti-CbFic2 and ani-H3 antibodies. **e** Intact MS analysis and quantification of time-resolved TS-H3_1-36aa_ AMPylation by CbFic2_E66G_ in the absence or presence of 5 bp, 10 bp, 20 bp, 40 bp, 60 bp dsDNA. 50 µM TS-H3_1-36aa_ was incubated with 5 µM of CbFic2_E66G_ in the presence of 5 µM DNA as indicated, 2 mM ATP, 4 mM MgCl_2_ at 37 °C for 22 h. AMPylation was measured by the mass increase of 329 Da, and AMPylated peaks were quantified by intensity after deconvolution. AMPylation was defined as a decrease in unAMPylated peptide over time, to reduce the complexity of multiple AMPylation. Each data point represents the mean of biological triplicates; error bars correspond to standard deviation. See Supplementary Fig. S3d and Supplementary Table 3. **f** Structural superposition of crystal structures of Z-DNA- (orange) and B-DNA-binding (red) HTH domains with that of the HTH domain of CbFic2 (cyan). B-DNA binding: human transcription factor E2F4 (PDB: 1cf7, chain C, red)^59^; Z-DNA binding: domain hZα_ADAR1_ of human double-stranded RNA-specific adenosine deaminase ADAR1 (PDB: 1qbj, chain D, orange)^60^. The conserved tyrosine of Z-DNA-binding domains is highlighted as stick. **g**, **h** CD measurement of 20 bp dsDNA with **g** 100% GC or **h** 40% GC content in the absence or presence of increasing amounts of CbFic2. 1 μM of dsDNA was mixed with CbFic2 to final concentrations of 1 μM ([P]/[N] = 1), 2 μM ([P]/[N] = 2) and 4 μM ([P]/[N] = 4). [P] and [N] stand for protein concentration and DNA concentration, respectively. Before each measurement, samples were incubated for 1 h at 25 °C. CD spectra between 230 and 320 nm were collected using a 0.75 cm quartz cell. See Supplementary Fig. S3e, f. **i**–**k** Analysis of the binding affinity of CbFic2, CbFic2_E66G ΔHTH_ and CbFic2_S22D S26D_ against **i** 20 bp, **j** 40 bp and **k** 60 bp dsDNA by fluorescence anisotropy. 1 nM 5‘-FITC-labeled DNA was mixed with a dilution series from 20 μM CbFic2 using a pipetting robot in a 384w format. Values were baseline corrected by anisotropy values of free DNA. Fit corresponds to “Specific binding with Hill slope” (3) (GraphPad Prism 8.0). Data shown correspond to the mean of technical triplicates, error bars to the standard deviation. See Supplementary Fig. S3g, h and Supplementary Table 2.

We determined the crystal structure of wild-type CbFic2 (Fig. 3a, PDB: 8CIL). In the asymmetric unit of the crystal, CbFic2 is present as a symmetrical homodimer which, according to the program PISA^52^, is the only probable assembly and is predicted to be stable. Within the dimer, the interface is composed of amino acids 20–43 aa in helix α1 within the N-terminal DUF4172 domain. According to an analysis by PDBsum^53^, residues S22 and S26 interact across the interface (Supplementary Fig. S3a, b). Therefore, the double mutant CbFic2_S22D S26D_ was designed to weaken the dimer interface.

The crystal structure reveals the predicted HTH domain to be a 2-stranded winged HTH domain where the tri-helical HTH motif is followed by a β-hairpin wing (Fig. 3a).

Affinity measurements of CbFic2 against ATP by fluorescence anisotropy using FAM-labeled ATP as ligand revealed a K_D_ of CbFic2 for ATP of around 640 nM (Supplementary Fig. S3c, Supplementary Table 2). As expected, the AMPylation-stimulating CbFic2_E66G_ mutant revealed an increased affinity for ATP with a K_D_ of approximately 170 nM. Deletion of the HTH domain in CbFic2 (CbFic2_ΔHTH_) had a similar effect to the E66G mutation with an increase in K_D_ to 280 nM, indicating that E66 might experience a stronger flexibility in the absence of the HTH domain.

### DNA binding stimulates CbFic2 activity

As described above, CbFic2 contains an HTH domain and is predicted to bind DNA. To obtain DNA-free protein, contaminating DNA and RNA from protein preparations expressed in *E. coli* were precipitated using polyethyleneimine (PEI)^54^. Without this step, CbFic2 tended to aggregate during purification, particularly at ionic strengths below 500 mM NaCl.

We first examined the ability of CbFic2 to bind nucleic acids and used the change in thermal unfolding as a proxy for their interaction. The addition of 20 base pair (bp) double-stranded (ds) DNA to the sample increases the melting temperature (T_M_) of CbFic2 by approximately 5 °C as determined by a thermal shift assay (TSA), suggesting DNA binding. The addition of DNA to a non-DNA-binding control protein, in this case the small Rab GTPase Rab1b, did not affect protein stability (Fig. 3b). While in the absence of DNA, CbFic2_E66G_ only poorly AMPylated TS-H3_1-36aa_ in vitro, the addition of a randomized 20 bp dsDNA oligomer with 40% GC content visibly increased AMPylation as analyzed by anti-AMP WB (Fig. 3c). The deletion of CbFic2’s HTH domain reduced AMPylation of TS-H3_1-36aa_ in the presence of DNA (Fig. 3c).

To analyze the effect of DNA binding on target affinity, we incubated histone H3.1 with decreasing concentrations of CbFic2_E66G_ and CbFic2_E66G ∆HTH_ in the absence or presence of DNA and analyzed histone AMPylation by WB (Fig. 3d). At 5 µM concentration, CbFic2_E66G_ and CbFic2_E66G ∆HTH_ both AMPylate their target in the presence of DNA, while the signal is strongly reduced for both in the absence of DNA. Similar behavior can be observed at 0.5 µM enzyme concentration, even if the overall AMPylation signal is weaker. At 0.1 µM enzyme concentration, however, CbFic2_E66G ∆HTH_ no longer shows target AMPylation, while CbFic2_E66G_ still AMPylates Histone H3 in the presence of DNA. To better understand the activation of CbFic2_E66G_ by dsDNA, AMPylation of TS-H3_1-36aa_ by CbFic2_E66G_ was monitored in a time-resolved manner via MS in the presence of dsDNA of 5 bp, 10 bp, 20 bp, 40 bp or 60 bp (Fig. 3e). AMPylation of TS-H3_1-36aa_ was quantified via the specific mass increase of 329 Da in intact MS. The increase in DNA length correlated with an increase in enzyme activation up until 20 bp dsDNA. While the addition of 5 bp dsDNA did not show any difference in the initial rate of product formation over time in comparison to DNA-free CbFic2_E66G_ (slope 0.03), 10 bp activated the enzyme more than 8-fold (slope 0.25), and 20 bp even stronger by over 22-fold (slope 0.68) (Supplementary Table 3, Supplementary Fig. S3d). Longer DNA up to 60 bp did not further stimulate enzyme activity in comparison to 20 bp (Fig. 3e).

PDBeFold^55^ analysis of the HTH domain of crystallized CbFic2 revealed structural similarity (Q-score >0.48) albeit low sequence similarity (%_seq_ < 18%) to Z-DNA-binding (ZDB) proteins, which belong to the same subclass of wHTH domains as proteins of the DeoR family^35^. ZDB proteins can be characterized by the presence of a conserved tyrosine residue within the third helix of the wHTH domain^56–58^. A structural superimposition of CbFic2’s HTH domain with the B-DNA-binding human transcription factor E2F4^59^ and the Z-DNA-binding domain hZα_ADAR1_ of human double-stranded RNA-specific adenosine deaminase ADAR1^60^ reveals that this tyrosine is not present at the conserved position in CbFic2, but moved by one helix turn toward the N-terminus (Y341), similar to the tyrosine in E2F4 but with a different rotation (Fig. 3f). To investigate a role of CbFic2 in Z-DNA binding we analyzed B- to Z-transition of DNA in the presence of CbFic2 via circular dichroism (CD) (Fig. 3g). CbFic2 did not induce a transition to Z-DNA in a ds(GC)_10_ oligomer, which would be indicated by a negative signal at 290 nm and a positive signal around 260 nm (for comparison, see control of ds(GC)_10_ in Z-form in the presence of 4 M NaCl (Supplementary Fig. S3e)). Instead, the CD spectra of 20 bp dsDNA, with 100% GC (Fig. 3g) or 40% GC content (Fig. 3h, control experiments in Supplementary Fig. S3f), show a CbFic2 concentration-dependent decrease in ellipticity at 249 nm and an increase at 279 nm, more typical for the A-form of DNA^61^. These CD measurements show that CbFic2 does not transition B-DNA to Z-DNA, but nevertheless changes DNA conformation with increasing protein concentrations.

To determine the affinity between CbFic2 and DNA, anisotropy measurements were performed using 5‘-fluoresceine isothiocyanate (FITC) labeled DNA as ligand (Fig. 3i–k). CbFic2 binds 20 bp dsDNA with 40% GC content with an approximate K_D_ of 220 nM (Supplementary Table 2). The omission of the HTH domain does not reduce DNA binding as expected (Fig. 3i). On the contrary, here the K_D_ is reduced to approximately half at 120 nM compared with the full-length proteins (Supplementary Table 2). The addition of a TS-tag to the N-terminus of the protein drastically reduces the affinity of TS-CbFic2_ΔHTH_ to a K_D_ of 750 nM, while the full-length protein’s K_D_ (TS-CbFic2) slightly decreases to 116 nM (Supplementary Fig. S3g, h; Supplementary Table 2). Thus, DNA binding seems to be not solely dependent on the presence of the HTH domain, since other protein regions show unspecific contribution, maybe due to ionic interaction with the highly basic surface of CbFic2. To ensure that the 5‘-FITC label of the DNA does not interfere with binding in anisotropy measurements, 40 bp and 60 bp dsDNA were also measured against CbFic2 (Fig. 3j, k). For full-length protein, longer DNA increased affinity from 220 nM (20 bp) to 109 nM (40 bp) and 37 nM (60 bp), while for CbFic2_ΔHTH_ the K_D_ increased from 120 nM (20 bp) to 204 nM (40 bp) or 195 nM (60 bp) with longer DNA (Supplementary Table 2). Mutation of the dimerization interface in CbFic2_S22D S26D_ only slightly reduced affinities toward DNA with K_D_ of 333 nM (20 bp), 96 nM (40 bp) and 64 nM (60 bp) (Fig. 3i–k, Supplementary Table 2).

### CbFic2 has AMPylase as well as deAMPylase activity

**Fig. 4:**
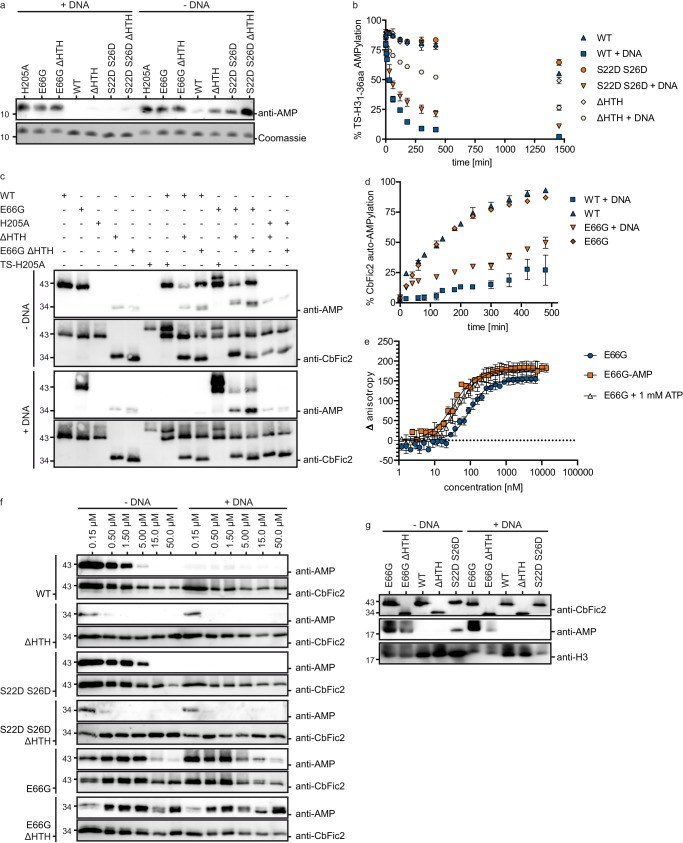
CbFic2 is regulated by a monomer/dimer equilibrium. **a** WB analysis of TS-H3_1-36aa_-AMP deAMPylation by CbFic2_H205A_, CbFic2_E66G_, CbFic2_E66G ΔHTH_, CbFic2, CbFic2_ΔHTH_, CbFic2_S22D S26D_ and CbFic2_S22D S26D ΔHTH_ in the absence or presence of DNA in vitro. 50 µM TS-H3_1-36aa_-AMP were incubated with 1 µM of the indicated CbFic2 versions with or without 4 µM 20 bp dsDNA in the presence of 1 mM MgCl_2_ at 37 °C for 8 h. 100 ng peptide was run on Tris-Tricine gels, blotted on PVDF and probed with an anti-AMP antibody. For loading controls, 1 µg of peptide was run on Tris-Tricine gels and stained with Coomassie. **b** Intact MS analysis and quantification of time-resolved TS-H3_1-36aa_-AMP deAMPylation by CbFic2, CbFic2_S22D S26D_ or CbFic2_ΔHTH_ in the absence or presence of DNA in vitro. 50 µM TS-H3_1-36aa_-AMP were incubated with 0.5 µM CbFic2 as indicated in the presence of 5 µM DNA, 1 mM MgCl_2_ at 37 °C for 24 h. DeAMPylation was measured by the mass loss of 329 Da, and peaks were quantified by intensity after deconvolution. deAMPylation was defined as an increase in unAMPylated peptide over time. Each data point represents the mean of biological triplicates; error bars correspond to standard deviation. See Supplementary Fig. S4a and Supplementary Table 3. **c** WB analysis of auto-AMPylation of CbFic2 in cis/trans. 0.3 µM CbFic2 versions as indicated were incubated alone or in the presence of another CbFic2 version, in the presence or absence of 2.5 µM 20 bp dsDNA, in the presence of 1 mM ATP and 1 mM MgCl_2_ for 8 h at 37 °C. 50 ng protein was run on Laemmli gels, blotted on PVDF and probed with an anti-AMP antibody. For loading controls, blots were stripped and incubated with an anti-CbFic2 antibody. See Supplementary Fig. S4b. **d** Intact MS analysis of auto-AMPylation of CbFic2 and CbFic2_E66G_ over the time course of incubation with ATP in the presence or absence of DNA. 0.2 mg ml^−1^ (4 µM) CbFic2 or CbFic2_E66G_ were incubated in 20 mM HEPES pH 7.5, 150 mM NaCl, 1 mM MgCl_2_, 1 mM TCEP, 1 mM ATP both in the presence and absence of 5 μM 20 bp dsDNA at 37 °C and analyzed by MS. The degree of automodification was detected by the specific mass gain of AMPylation of 329 Da. AMPylation was quantified by the ratio of the specific signal intensity to the total intensity of all CbFic2 signals. As CbFic2 shows multiple auto-AMPylations (see supplement for detailed depiction), data represent the decrease of unAMPylated CbFic2. Each data point represents the mean of biological triplicates; error bars correspond to standard deviation. See Supplementary Fig. S4c. **e** Fluorescence anisotropy analysis of the influence of CbFic2 auto-AMPylation and the presence of ATP on DNA binding. 1 nM 5‘-FITC-labeled 20 bp dsDNA was mixed with a dilution series from 20 μM auto-AMPylated CbFic2_E66G_-AMP or CbFic2_E66G_ in the presence of 1 mM ATP using a pipetting robot in a 384w format. Values were baseline corrected by anisotropy values of free DNA. Fit corresponds to “Specific binding with Hill slope” (3) (GraphPad Prism 8.0). Data shown correspond to the mean of technical triplicates, error bars to the standard deviation. See Supplementary Table 2. **f** WB analysis of concentration-dependent auto-AMPylation of CbFic2. From a starting concentration of 50 µM CbFic2 versions as indicated, protein was diluted to 15 µM, 5 µM, 1.5 µM, 0.5 µM and 0.15 µM, and incubated in the presence or absence of 50 µM, 15 µM or 5 µM or 4 µM (for protein concentrations of or below 1.5 µM) 20 bp dsDNA, respectively, in the presence of 1 mM ATP and 1 mM MgCl_2_ for 8 h at 37 °C. 50 ng protein was run on Laemmli gels, blotted on PVDF and probed with an anti-AMP antibody. For loading controls, blots were stripped and incubated with an anti-CbFic2 antibody. **g** WB analysis of Histone H3.1 AMPylation by CbFic2_E66G_, CbFic2_E66G ΔHTH_, CbFic2, CbFic2_ΔHTH_, CbFic2_S22D S26D_ at low enzyme concentrations in the absence or presence of DNA in vitro. 0.1 mg ml^−1^ Histone H3.1 was incubated with 0.5 µM of the indicated CbFic2 versions with or without 5 µM 20 bp dsDNA in the presence of 1 mM ATP and 1 mM MgCl_2_ at 37 °C for 8 h. 100 ng Histone H3.1 were run on Tris-Tricine gels, blotted on PVDF and probed with an anti-AMP antibody. For loading controls, blot was stripped, cut into strips and reprobed with anti-Histone H3 and anti-CbFic2 antibodies.

When incubating purified AMPylated TS-H3_1-36aa_ (TS-H3_1-36aa_-AMP) with different CbFic2 mutants in the absence or presence of DNA, WB analysis against AMPylated protein shows that wild-type CbFic2, but not CbFic2_E66G_ is capable of deAMPylation. This deAMPylase activity is stimulated by the addition of DNA. Even after deletion of the HTH domain or mutation of the dimerization interface, deAMPylation activity is still detectable via WB (Fig. 4a). Time-resolved MS analysis of TS-H3_1-36aa_-AMP deAMPylation by CbFic2 shows a comparably low activity among wild type CbFic2, the dimer interface mutant CbFic2_S22D S26D_ and CbFic2_ΔHTH_ in the absence of DNA (Fig. 4b). This lack of deAMPylating activity among all DNA-free CbFic2 versions translates into next to no negative slope when calculating the initial rate of product formation over time by a linear fit to the early phase of the graph, assuming steady-state conditions (Supplementary Fig. S4a, Supplementary Table 3). However, the addition of 20 bp dsDNA increases the deAMPylation activity of CbFic2 (slope −0.75), but less so with the mutation of the dimer interface in CbFic2_S22D S26D_ (slope −0.55) and even less with a deletion of the HTH domain in CbFic2_ΔHTH_ (slope −0.16) (Fig. 4b, Supplementary Fig. S4a, Supplementary Table 3). In consequence, deAMPylation by CbFic2 is influenced both by dimerization as well as HTH domain-dependent and independent DNA binding.

Although CbFic2 showed no measurable AMPylation activity toward TS-H3_1-36aa_ (Fig. 3c), it is capable of automodification in the absence of DNA (Fig. 4c, for control measurements without ATP see Supplementary Fig. S4b). Thus, CbFic2 appears to bind ATP in an AMPylation-competent manner despite the presence of the obstructive acidic side chain of E66. The addition of DNA suppresses the auto-AMPylation signal in CbFic2 but not the deAMPylation-defective CbFic2_E66G_ at the assay concentration of 0.3 µM (Fig. 4c), hinting at a stimulation of deAMPylation activity instead of a sole suppression of AMPylation activity. The auto-AMPylation activity of CbFic2 at 0.3 µM is drastically reduced with the deletion of the HTH domain, independent of the presence of E66 and/or DNA (CbFic2_E66G ΔHTH_ and CbFic2_ΔHTH_) (Fig. 4c). Co-incubation of different CbFic2 mutants reveals that automodification is taking place in trans as well as *in cis*, since co-incubation of CbFic2_E66G_ with TS-CbFic2_H205A_ results in a modification of the inactive TS-CbFic2_H205A_ while CbFic2 in the absence of DNA only AMPylates itself but not TS-CbFic2_H205A_ (Fig. 4c). At the same time, auto-AMPylation of wild type CbFic2 in the absence of DNA seems to be reduced in the presence of CbFic2_ΔHTH_ but not CbFic2_E66G ΔHTH_, hinting at deAMPylation also being possible in trans (Fig. 4c). Time-resolved analysis of CbFic2 automodification via intact MS shows that CbFic2 is as active in regards to auto-AMPylation as CbFic2_E66G_ at a concentration of 4 µM (Fig. 4d, Supplementary Fig. S4c). In the presence of DNA, auto-AMPylation is reduced for both CbFic2_E66G_ as well as CbFic2 (Fig. 4d). While automodification of Fic enzymes might be only an in vitro artifact without physiological consequence, it still can serve as readout for AMPylation-competent ATP binding. While the addition of DNA impacts auto-AMPylation activity (Fig. 4c, d), DNA-affinity measurements by fluorescence anisotropy reveal that automodification of CbFic2 does not influence the affinity toward DNA more than the addition of ATP can achieve (Fig. 4e). In the process of sample preparation for these anisotropy experiments, CbFic2 could only be auto-AMPylated at reduced concentrations (Fig. 4c, d); and concentrating CbFic2 samples for anisotropy measurements resulted in loss of auto-AMPylation, so that measurements could only be performed for CbFic2_E66G_, whose auto-AMPylation remained stable (Fig. 4e). This observation, along with the observed differences in DNA impact on CbFic2_E66G_ auto-AMPylation between 0.3 µM (Fig. 4c) and 4 µM (Fig. 4d), led us to investigate a concentration dependent effect of auto-AMPylation vs auto-deAMPylation: in WB analysis of auto-AMPylated CbFic2, automodification of CbFic2 can only be observed below an assay concentration of 5 µM CbFic2 (Fig. 4f). Mutation of the dimerization interface in CbFic2_S22D S26D_ slightly increases the threshold of auto-AMPylation to above 5 µM. The automodification is in either case completely suppressed by the addition of DNA (Fig. 4f).

Keeping in mind that CbFic2 forms a dimer in the crystal structure (Fig. 3a), and human FICD was shown to be regulated by a monomer-dimer equilibrium, where the monomer is an AMPylase and the dimer a deAMPylase^16^, we hypothesized that CbFic2 might be similarly regulated: At low concentrations below the K_D_ of dimerization, and in the absence of DNA, CbFic2 might be a monomer and therefore an AMPylase. DNA might induce a dimer via binding to the HTH domain and thereby shift the equilibrium toward a dimer, just as DNA-free enzyme concentrations above the K_D_ of dimerization, stimulating deAMPylation. The shift from auto-AMPylation to loss of signal around 5 µM might therefore represent a shift from monomer to dimer (Fig. 4f).

According to this hypothesis, CbFic2 should be capable of target AMPylation at low concentrations in the absence of DNA, and the disruption of the dimer interface should increase that effect. Indeed, when incubating Histone H3.1 with 0.5 µM CbFic2 (Fig. 4g), the dimer interface mutant CbFic2_S22D S26D_ shows Histone H3.1 AMPylation in the absence of DNA, even though the autoinhibitory E66 is still present. In the presence of DNA, target AMPylation by CbFic2_S22D S26D_ is not detectable. Together with the observation that target deAMPylation in the presence of DNA after mutation of the dimerization interface is weakened but still clearly present (Fig. 4b), and autoAMPylation by CbFic2_S22D S26D_ is still suppressed at higher concentrations (Fig. 4f), we speculate that the CbFic2_S22D S26D_ mutation only weakens but not destroys dimer formation and that DNA binding strongly induces dimer formation via the HTH domain and therefore overrides CbFic2_S22D S26D_’s weakened dimerization interface at the analyzed concentrations.

### CbFic2 is regulated by DNA-binding-induced dimerization

**Fig. 5:**
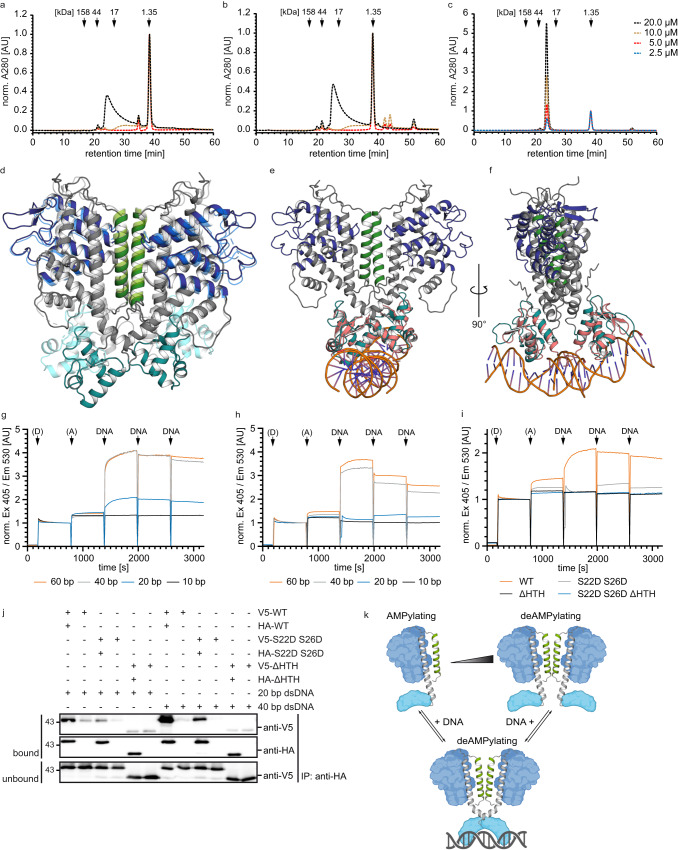
CbFic2 shows DNA-induced dimerization. **a**–**c** Analysis of concentration-dependent dimerization of **a** CbFic2, **b** CbFic2_S22D S26D_ and **c** CbFic2_ΔHTH_ by analytical size exclusion chromatography. CbFic2 was injected at indicated concentrations onto a Superdex 75 pg 10/300 (Cytiva), run at 0.5 ml min^−1^ in 20 mM HEPES pH 7.5, 150 mM NaCl, 1 mM MgCl_2_, 1 mM TCEP, and protein was detected by absorbance at 280 nm. Data intensity was normalized to the internal control of vitamin B_12_ (t_R_ = 38.5 min). Arrows indicate the gel filtration standard (BioRad) comprising bovine γ-globulin (MW 158 kDa), chicken ovalbumin (MW 44 kDa), horse myoglobin (MW 17 kDa) and vitamin B_12_ (MW 1.35 kDa). **d** Superimposition of CbFic2 crystal structure (lighter shades; light green dimerization interface; middle blue Fic domain; cyan HTH domain) with AlphaFold model (darker shades; dark green dimerization interface; dark blue Fic domain; turquoise HTH domain) shows structural flexibility in the HTH domain by a kink in the long connecting helix between HTH domain (turquoise) and Fic domain (blue). **e** Proposed model for DNA-binding induced dimerization. Superimposition of HTH domain (turquoise) of CbFic2 AlphaFold structure with human transcription factor E2F4 (PDB: 1cf7, chain C, red)^59^ bound to B-DNA suggests that bound 20 bp DNA to CbFic2 might span across both monomers and thereby stimulate dimerization. **f** 90° turn of (**e**) around the vertical axis. **g**–**i** Analysis of DNA-induced dimerization by in-solution FP-fusion FRET time course measurements in 20 mM HEPES pH 7.5, 150 mM NaCl, 1 mM MgCl_2_, 1 mM TCEP. After 3 min, donor CyPet-CbFic2 or its mutants (D) and after another 10 min, acceptor YPet-CbFic2 or its mutants (A) were added at concentrations of 0.5 µM (resulting in total CbFic2 concentrations of 1 µM). After another 10 min incubation, 4 µM of dsDNA was added three times in succession (DNA), with each incubation lasting 10 min. **g** represents 0.5 µM CyPet- and YPet-CbFic2 followed by 4 µM 10 bp, 20 bp, 40 bp or 60 bp dsDNA, **h** 0.5 µM CyPet- and YPet-CbFic2_S22D S26D_ followed by 4 µM 10 bp, 20 bp, 40 bp or 60 bp dsDNA and **i** 0.5 µM CyPet- and YPet-CbFic2 or -CbFic2_S22D S26D_ or -CbFic2_ΔHTH_ or -CbFic2_S22D S26D ΔHTH_ each followed by 4 µM 20 bp dsDNA. Measurements were performed at 25 °C, with an excitation wavelength of 405 nm and an emission wavelength of 530 nm. Intensities were normalized to the value at 760 s corresponding to the endpoint intensity of donor addition. **j** WB analysis of hetero-dimer formation after co-IP of recombinant HA- and V5- CbFic2 using an anti-HA antibody. 0.2 µM HA- and 0.2 µM V5-tagged CbFic2, CbFic2_S22DS26D_ or CbFic2_ΔHTH_, respectively, were incubated with 4 µM 20 bp or 40 bp dsDNA in the presence of 2 µg anti-HA antibody in 20 mM HEPES pH 7.0, 150 mM NaCl, 1 mM MgCl_2_, 20% (v/v) glycine, 0.1% (v/v) Tween 20 and precipitated by protein A/G magnetic beads. For each CbFic2 version, a control assay without HA-tagged protein was prepared in addition, to control for unspecific binding of CbFic2. HA-tagged CbFic2 and its binding partners were eluted by 0.1 M glycine, pH 2.0. Samples were separated by 12% Tris-glycine gels, blotted, blocked and detection of the potential heterodimer was performed via anti-V5 tag antibody. **k** CbFic2 regulation by DNA-induced dimerization. Suggested model of CbFic2 regulation on the basis of our data.

In order to analyze the dimerization of CbFic2 and the impact of the S22D S26D mutation and the loss of the HTH domain, we subjected CbFic2, CbFic2_S22D S26D_ and CbFic2_ΔHTH_ to analytical size exclusion chromatography (Fig. 5a–c). For both CbFic2 and CbFic2_S22D S26D_, a shift in retention time to higher MW can be observed with rising protein concentration (Fig. 5a, b). The same is true for CbFic2_E66G_ (Supplementary Fig. S5a). However, they all show secondary interaction with the column matrix as demonstrated by strong peak tailing, low peak height and delayed retention times in relation to their MW, which could be resolved by high NaCl (500 mM) or arginine (200 mM) concentrations (Supplementary Fig. S5b, c). Unfortunately, under these buffer conditions, the concentration-dependent shift in retention time was also abrogated. Looking at the dimerization interface of CbFic2’s crystal structure (Supplementary Fig. S3a, b), high salt conditions would destroy the polar and electrostatic interaction as well as the hydrogen bonds involved in the interface. While this experiment strengthens the theory that CbFic2 shows concentration-dependent dimerization and that CbFic2_S22DS26D_ is still capable of dimerization despite the mutations to its dimer interface, the secondary interaction prevents quantification of MW or determination of a potential K_D_ of dimerization. CbFic2_ΔHTH_ on the other hand shows no secondary interaction and elutes from the column as a clear monomer with no concentration-dependent effect (Fig. 5c), so that mutation of the dimerization interface in CbFic2_S22D S26D ΔHTH_ has no effect on the retention behavior (Supplementary Fig. S5d).

At first glance, superposition of the CbFic2 structure with crystallized HTH domains in complex with DNA makes dimer formation of CbFic2 via the crystal dimer interface (Fig. 3a, Supplementary Fig. S3a, b) unlikely to be compatible with a simultaneous binding of both HTH domains to the same DNA-strand of about 20 bp, if not for a major rearrangement of CbFic2 or bending of DNA. Despite our best efforts, the co-crystallization of CbFic2 with DNA remained unsuccessful. However, AlphaFold prediction of CbFic2 results in five models (see Supplementary Data), which show flexibility in the orientation of the HTH domain in relation to the Fic and DUF domain, communicated by a kink in the long connecting helix between the HTH and Fic domains (Fig. 5d). Superposition of the CbFic2 model that has the most extreme HTH domain orientation in comparison to the crystal structure with crystallized human transcription factor E2F4 bound to B-DNA^59^ reveals alignment of the bound DNA in one horizontal line across the HTH domains of the dimer, allowing for the possibility that a bound 20 bp DNA might span across both CbFic2 monomers and thereby stimulate dimerization (Fig. 5e, f). This might explain why 5 bp DNA does not induce any CbFic2 activation, and binding of 10 bp DNA only achieves less than half of the initial rate that binding of 20 bp DNA yields, while 40 bp and 60 bp do not stimulate the enzyme’s activity further (Fig. 3e, Supplementary Table 3). According to analysis of the dimer interface of this AlphaFold model by PDBsum^53^, the residues involved in dimer formation change in comparison to our crystal structure: Where in the crystal structure S22 and S26 interact via non-bonded interaction with their counterpart (Supplementary Fig. S3a), in the AlphaFold model S22 interacts now with E18, and S26 with D189 via hydrogen bonds (Supplementary Fig. S5e). In addition, the C-terminus becomes involved in dimer formation, where Q44 builds a hydrogen bond with E373 (Supplementary Fig. S5e).

To verify this model of DNA-binding induced dimerization, we designed an in vitro in-solution Förster resonance energy transfer (FRET) assay using the fluorescent proteins (FP) CyPet and YPet, each individually fused to the N-terminus of CbFic2, CbFic2_S22D S26D_, CbFic2_ΔHTH_ or CbFic2_S22D S26D ΔHTH_, as donor and acceptor, respectively^62^. In a time-resolved experiment, fluorescence at a donor excitation of 405 nm and acceptor emission of 530 nm was followed throughout the successive addition of donor, acceptor and—three times in a row—dsDNA to guarantee protein saturation and observe the dilution effect. When a CbFic2 dimer is formed, the FP-FRET label at the N-termini of CyPet-CbFic2 and YPet-CbFic2 are expected to get into close proximity, emitting FRET signal. Using CbFic2, the addition of 20 bp dsDNA or longer to the reaction mixture clearly induces a FRET signal for the first DNA addition, while 10 bp dsDNA does not cause a signal change (Fig. 5g). This effect is true for a concentration range of 0.2–2 µM (sum of CbFic2 protein 0.4–4 µM) (Supplementary Fig. S5f). The mutation of the dimerization interface in CbFic2_S22D S26D_ changes the behavior in response to 20 bp dsDNA, where the fluorescent signal gets quenched with the first addition and increased with the second addition of 20 bp dsDNA, while the addition of 40 bp and 60 bp dsDNA imitate the behavior of wild type CbFic2 albeit with lower intensities (Fig. 5h). Comparing wild type CbFic2 with the mutants CbFic2_S22D S26D_, CbFic2_ΔHTH_ and CbFic2_S22D S26D ΔHTH_ at 0.5 µM concentration and 20 bp dsDNA addition illustrates that DNA induces a dimer in wild type CbFic2, but not in CbFic2 lacking the HTH domain (Fig. 5i). This effect is also observable for 40 bp dsDNA (Supplementary Fig. S5g). Control measurements with the FRET label alone, not fused to CbFic2, show no increase in signal upon any DNA addition at any concentration and thus confirm that the dimerization upon DNA addition is CbFic2 dependent (Supplementary Fig. S5h, i).

To better understand the behavior of CbFic2_S22D S26D_ upon 20 bp vs 40 bp dsDNA displayed in the FRET experiments, and the differences toward wild type CbFic2, we employed a co-IP experiment where HA- and V5-tagged CbFic2 are incubated with dsDNA before being precipitated by an anti-HA antibody. To control for unspecific binding, all assays were also performed without the addition of HA-tagged protein. Hetero-dimer formation was detected by an anti-V5 WB. CbFic2 in the presence of 20 bp dsDNA shows a clear band in the anti-V5 WB that is visibly stronger in the presence of 40 bp dsDNA. CbFic2_S22D S26D_, on the other hand, shows a clear anti-V5 signal only in the presence of 40 bp dsDNA, albeit with lower intensity than the wild type, but only a faint band in the presence of 20 bp dsDNA. CbFic2_ΔHTH_ shows no increased band in the presence of either 20 bp or 40 bp dsDNA, as expected (Fig. 5j). The co-IP assays thus confirm the FRET experiments and shed further light on the influence of the dimerization interface mutation.

We therefore confirm that CbFic2 is regulated by a monomer-dimer transition, where monomeric CbFic2 AMPylates histones and dimeric CbFic2 acts as deAMPylase (Fig. 5k). CbFic2 forms dimers either at increased concentrations (Fig. 5a) or in the presence of dsDNA (Fig. 5g, j), and the disruption of the dimerization helix at positions S22 and S26 only weakens but not abolishes dimerization (Fig. 5b, h, j). The presence of the HTH domain is necessary since deletion causes monomerization independent of concentration (Fig. 5c) or the presence of dsDNA (Fig. 5i, j).

## Discussion

Here we show that CbFic2 is an AMP transferase targeting histones, which is regulated by the binding of DNA. CbFic2 AMPylates Histone H3 in vitro and in cellulo at S10 and S28 within the ARKS sequence motif of the N-terminal tail. CbFic2 also AMPylates Histone H2B in vitro and mH2A1 in cellulo. CbFic2 binds DNA with nanomolar affinity via its HTH domain, although nonspecific binding contributes outside the HTH domain.

Random DNA sequences were used in the activity assays and DNA-binding studies of CbFic2 (Supplementary Tables 4 and 5). The high affinity for these random DNA sequences could indicate that CbFic2 is a nonspecific DNA-binding protein and does not rely on a specific sequence, e.g., a promoter region, for regulation. In this case, DNA binding could merely serve to ensure general proximity to target proteins such as histone H3 to increase residence time at the target protein. On the other hand, many DNA-binding proteins are able to interact with DNA outside their specific sequence as part of their strategy to find a small region on a much larger DNA molecule through nonspecific binding mechanisms such as sliding, hopping, or intersegmental transfer^63^. The intensively studied transcription factor p53 exhibits variance in the mid- to low-nM range with respect to its affinity for various specific DNA sequences, with no significant difference compared to its affinity for nonspecific sequences^64,65^. Although the p53 DNA-binding domain does not resemble an HTH domain, it is conceivable that CbFic2, despite its high affinity for nonspecific DNA, also recognizes certain DNA motifs in a sequence-specific manner. Nonspecific electrostatic interactions with DNA can disguise binding cooperativity in anisotropy data, and cooperativity occurs increasingly for specific DNA sequences^65^. Thus, the lack of cooperativity of CbFic2 in our anisotropy data despite evidence for DNA-induced dimerization could indicate a high degree of nonspecific interaction. In p53, nonspecific DNA binding is mediated by the C-terminus of the protein and regulated by PTMs such as acetylation and phosphorylation within the C terminus, so that despite the absence of the nuclear DNA-binding domain, the transcription factor still exhibits measurable affinity for DNA^66–68^. Despite the lack of homology of p53 with CbFic2, this binding and regulatory mechanism could explain why CbFic2 without HTH domain still binds to DNA. As localization experiments using GFP-tagged CbFic2 in microscopy (Supplementary Fig. 1a) show, truncation of CbFic2’s N-terminus in CbFic2_41-378_ and CbFic2_86-378_ leads to loss of nuclear localization. In addition, AMPylation by these constructs can no longer be detected in a cellular environment (Supplementary Fig. 1d–g). While these data might suggest that the N-terminal DUF domain also plays a role in localization, the structural information on CbFic2 is the basis for caution: Deletion of the first 40 or 85 amino acids, respectively, leads with high probability to high structural rearrangement, if not to loss of folding. Trying to express the constructs recombinantly in *E. coli* leads to low solubility and increased degradation and was not successful in our hands. Nevertheless, DNA binding (Fig. 3i–k) as well as deAMPylation activity (Fig. 4a, b) despite the removal of the HTH domain in the CbFic2_∆HTH_ construct suggests that the N-terminus still plays a role in DNA binding and enzyme regulation. In favor of this interpretation, the addition of a twin-strep tag to the N-terminus leads to reduced DNA affinity in TS-CbFic2_∆HTH_ (Supplementary Fig. S3g) and the addition of DNA to CbFic2_∆HTH_ or CbFic2_E66G ∆HTH_ stimulates deAMPylation (Fig. 4b) and AMPylation (Fig. 3d) activity, respectively. At the same time, however, loss of the HTH domain changes the affinity toward ATP (Supplementary Fig. S3c) and therefore has a clear impact on the enzyme’s active center. A side-by-side comparison of end-point AMPylation by CbFic2_E66G_ or CbFic2_E66G ∆HTH_, where CbFic2_E66G ∆HTH_ loses its ability of target AMPylation at low enzyme concentrations (Fig. 3d), suggests that the affinity toward the target might be overall reduced with the loss of the HTH domain. In addition, analytical SEC indicates that CbFic2_∆HTH_ cannot dimerize anymore despite the presence of the DUF dimerization motif, and in-solution FP-FRET and co-IP further corroborate that DNA-induced dimerization is no longer possible. We therefore suggest that the measurable DNA-binding and DNA-stimulated enzyme activity in CbFic2_∆HTH_ is the unspecific effect of a deregulated enzyme with a basic pI, whose positively charged surface interacts with negatively charged DNA, to bind a positively charged target. After all, despite its monomeric state, CbFic2_∆HTH_ is not capable of either auto- (Fig. 4f) or target-AMPylation (Fig. 3c) if the inhibitory E66 is present.

When studying protein-DNA interactions, it is generally difficult to accurately infer physiological consequences from in vitro data since the chromatin environment and accessibility in the cell are greatly altered by the presence of nucleosomes, PTMs, and other chromatin-interacting factors. Allosteric effects in DNA can cause the binding of one protein to affect the binding of another, for example, by deforming the double-stranded helix^69^. Since it seems likely that CbFic2 binds near nucleosomes to modify histone H3, this factor probably also plays an important role in DNA recognition, binding, and activation of CbFic2. If, however, CbFic2 was a specific DNA-binding protein, the affine albeit nonspecific binding of DNA in our assays may not be sufficient to induce the structural change required for maximal enzymatic activity. Future studies focusing on a specific binding sequence as well as secretion of CbFic2 during *Coxiella* infection would therefore be of special interest and might shed some light on the unresolved physiological relevance and consequence of CbFic2 activity.

The domain structure of CbFic2 is predicted to be shared by a variety of Fic enzymes of bacterial species, e.g., SoFic from *Shewanella oneidensis*^70^ and CccR from *Yersinia pseudotuberculosis*^71^ although structure-based sequence alignment similarity according to PDB is low. Similar to CbFic2, their crystal structures include a C-terminal wHTH domain in addition to the N-terminal DUF4172 domain. SoFic is a dimer in the crystal structure, in which the subunits interact via the same N-terminal helix of the DUF4172 domain as CbFic2^70^. However, DNA-binding for SoFic has not been experimentally demonstrated to date. CccR is a toxin and T6SS effector, that auto-represses its own expression via the P_cccR_ promotor but causes growth arrest in neighboring bacterial cells by AMPylating the cell division protein FtsZ^71^. An electrophoretic mobility shift assay showed binding to palindromic sequences within the P_cccR_ promotor but isothermal titration calorimetry determined an approximately 10-fold higher K_D_ of 2.6 µM for DNA than that of CbFic2 (Supplementary Table 2).

We show that CbFic2 is a bifunctional enzyme, capable of both AMPylation and deAMPylation. Similar to reports on other Fic proteins, deAMPylation in CbFic2 depends on the presence of the inhibitory glutamate E66^13,14^. Furthermore, mutation of the CbFic2 dimer interface induces target AMPylation and reduces target deAMPylation. Together with the concentration-dependent auto-AMPylation behavior of CbFic2, which shows auto-AMPylation only at low enzyme concentration, it hints at regulation by a monomer-dimer transition. This is reminiscent of the regulation of FICD, which is an AMPylase in the monomeric form and a deAMPylase in the dimeric state. Impairment of CbFic2-dimerization via interface mutations shifts the critical concentration for auto-AMPylation to higher concentrations, demonstrating that CbFic2 is an AMPylase in the monomeric form. However, the addition of DNA apparently induces CbFic2-dimerization and thus stimulates target deAMPylation and abrogates auto-AMPylation.

The loss of the HTH domain results in a higher ATP-affinity of CbFic2, an effect similar to the mutation of the inhibitory residue E66. We speculate that the HTH domain might influence the flexibility of E66; in contrast, mutation of the dimer interface results in a lower ATP affinity, so both dimerization and the HTH domain may influence ATP binding. Unfortunately, our efforts to determine whether DNA addition increases the ATP affinity of CbFic2 were not successful since the low solubility of the CbFic2:DNA complex hindered affinity measurements.

As previous publications have already suggested, regulation by monomerization and dimerization might play a more general regulatory role for class II Fic-proteins^16^. Since deAMPylation has only been investigated for a fraction of all published class II Fic-enzymes, data for the regulation of deAMPylation are scarce, with FICD being the notable exception. However, CdFic from *Clostridium difficile* has been reported to be capable of auto-AMPylation despite the presence of an autoinhibitory helix, and a mutation of the dimer interface increased automodification similar to the behavior of CbFic2^72,73^. The authors attributed CdFic’s behavior to an atypical phosphate binding of ATP and exposure of auto-AMPylation sites after disruption of dimerization;^73^ but a concentration-dependent effect, as well as deAMPylation activity, were not investigated. More recently, CccR from *Y. pseudotuberculosis* showed a stoichiometry of protein binding to DNA of 2:1 in isothermal titration calorimetry experiments, and mutation of the dimer interface reduced the repression effect of CccR on the P_cccR_ promotor while having little effect on CccR’s toxicity by AMPylation^71^. The authors concluded that the regulatory and toxic activities of CccR were independent^71^. However, the data could also be interpreted as dimerization at the respective promotor DNA within *Y. pseudotuberculosis*, inducing a deAMPylating dimer and therefore no toxicity in addition to the reported repression of transcription. In the absence of the promotor DNA in *E. coli* cells, CccR might monomerize and therefore AMPylate with toxic effects, and a dimerization mutant would have no additional toxic effect as reported. Examining deAMPylation, and obtaining in vitro data on DNA-free vs. DNA-bound behavior as well as concentration-dependent AMPylation by CccR in the future would provide valuable clarification. As these observations are only speculative at the moment, examining deAMPylation and dimerization more broadly in the future would be of high importance for the understanding of class II Fic enzyme regulation.

In summary of our data, we suggest that CbFic2 can exist as either an AMPylating monomer or a deAMPylating dimer in solution. Upon DNA binding, this equilibrium might be shifted toward a deAMPylating dimer with a strong tendency toward aggregation at higher concentrations. Our data show that CbFic2 AMPylates histones in mammalian cells, but the physiological context and relevance of this modification remain unclear.

## Methods

### Statistics and reproducibility

Anti-AMP IP for LC-MS/MS analysis was performed in three independent biological replicates (*n* = 3). TSA assay data represents technical triplicates. Time-resolved (de)AMPylation analyzed by LC-MS was performed as biological triplicates. CD measurements were performed in technical triplicates. Anisotropy data is shown as technical triplicates. Anisotropy measurements were repeated at least as biological duplicates to ensure data reproducibility. Time-resolved auto-AMPylation analyzed by LC-MS was performed as biological triplicates. Time-resolved and concentration-dependent assays analyzed by WB were performed as biological duplicates, representative blots are shown. Analytical size exclusion chromatography was performed as biological duplicates; representative chromatograms are shown. At least technical duplicates were produced and representative data is shown for time-resolved FP-FRET. Co-IP experiments of HA- and V5-tagged CbFic2 were performed as technical duplicates, representative blots are shown. Microscopy data are based on single experiments. Anti-H3 and anti-myc IPs and *Coxiella* infection experiments were performed only once. All other WB data is representative of at least biological duplicates.

### Molecular biology

Unless otherwise indicated, all genes were synthesized (Integrated DNA Technologies, IDT) and codon-optimized for expression in *E. coli* by omitting rare codons. Plasmids were cloned by sequence- and ligation-independent cloning (SLIC)^74^ using Q5® High-Fidelity DNA Polymerase (New England Biolabs (NEB)) and T4 DNA Polymerase (NEB). Point mutations, insertions and deletions were introduced by Q5® Site-Directed Mutagenesis Kit (NEB). PCRs were performed using the T100 thermal cycler (BioRad). DNA was purified from gel using the Monarch® DNA Gel Extraction Kit (NEB). Cloned constructs were transformed into chemically competent (Mix & Go, Zymo Research) Mach1 cells, or NEB® stable cells (NEB) for stable cell line generation, and positive clones were determined by complete Sanger Sequencing (GATC or Microsynth). For studies of CbFic2 in HEK293 and Cos7 cells, CbFic2 gene (*CBU_0822*) constructs were cloned into pAc-GFP-N1 (Takara) using XhoI (NEB) and HindIII (NEB) cloning sites for transient expression in mammalian cells with a C-terminal GFP-fusion, resulting in a protein product of MW 72.2 kDa. N-terminal GFP-fusion constructs were built in a two-step process. First GFP was inserted upstream of the MCS of pAc-GFP-N1 and then respective gene constructs were cloned using XhoI and NotI (NEB) cloning sites (protein product MW 70.9 kDa). Deletions and point mutations were introduced as indicated. CbFic2 constructs with truncations at the N-terminus were labeled C-terminally and C-terminal truncations were labeled N-terminally with GFP. For generation of the H3.1-CT-MYC plasmid and its point mutations, mH2A1.2 in mH2A1.2-CT-MYC (Addgene plasmid # 45168)^47^ was replaced by human H3.1, and point mutations were introduced as described. For generation of stable THP-1 cells, CbFic2 and respective mutants were introduced into the multiple cloning site (MCS) of pCW57-GFP-P2A-MCS plasmid (Addgene plasmid #89181)^75^ by SLIC, before the simian virus 5 (V5) tag was inserted directly behind the 2A sequence from porcine teschovirus 1 (P2A), resulting in a P-V5-G-A-H-R-L-M-CbFic2 protein product of MW 45.7 kDa. For bacterial expression, CbFic2 was introduced into the pSF (Oxford Genetics) backbone, preceded either by a N-terminal His_10_-GFP-TEV sequence (MW 73.9 kDa), which after Tobacco Etch Virus (TEV) cleavage resulted in an untagged CbFic2 protein product fused to G-H-M at the N-terminus (CbFic2, MW 43.9 kDa), or by a N-terminal His_10_-GFP-TEV-TS-EK sequence (MW 77.7 kDa), which after TEV cleavage resulted in a CbFic2 protein product fused to a Twin-Strep (TS) tag via an enterokinase cleavage site (EK) with the sequence G-H-M-TS-G-A-EK-H-M at the N-terminus (TS-CbFic2, MW 47.8 kDa), or by a N-terminal His_10_-GFP-TEV-HA sequence (MW 74.9 kDa), which after TEV cleavage resulted in a CbFic2 protein product fused to a human influenza hemagglutinin (HA) tag (YPYDVPDYA) with the sequence G-HA-G-A-M at the N-terminus (HA-CbFic2, MW 45 kDa), or by a N-terminal His_10_-GFP-TEV-V5 sequence (MW 75.3 kDa), which after TEV cleavage resulted in a CbFic2 protein product fused to a V5 tag (GKPIPNPLLGLDST) with the sequence G-V5-G-A-M at the N-terminus (V5-CbFic2, MW 45.3 kDa). For in solution FP-FRET, GFP was replaced in the pSF-His_10_-GFP-TEV constructs by YPet and CyPet from pYPet-His (Addgene plasmid # 14031) and pCyPet-His (Addgene plasmid # 14030)^76^, respectively, resulting in a protein product of MW 73.3 kDa for both, YPet-CbFic2 and CyPet-CbFic2. For the CyPet-YPet positive control, CbFic2 was then replaced in the pSF-His_10_-CyPet-TEV-CbFic2 construct by YPet, resulting in a protein product of MW 56.4 kDa. TS-H3_1-20aa_ and TS-H3_1-36aa_ were generated by cloning the respective amino acids of human Histone H3.1 into the pSF backbone, preceded by a N-terminal His_10_-GFP-TEV-TS-EK sequence (MW 36.3 kDa and 37.7 kDa, respectively), which after TEV cleavage resulted in a Histone H3.1 protein product fused to G-H-M-TS-G-A-EK-H-M at the N-terminus (MW 6.4 kDa and 7.7 kDa, respectively). For evaluation of the AMP-modification site in histone H3 synthesized complementary oligonucleotides coding the histone H3 peptide H3_1-20aa_ sequence with respective mutations were introduced into a pGATEV plasmid^77^, leading to constructs with an N-terminal His_6_-GST-tag (MW 31.6 kDa) followed by a TEV cleavage site for separation of the untagged H3_1-20aa_ peptide from the glutathione S-transferase (GST) tag leaving only a G-H-M- at the N-terminus (MW 2.5 kDa).

#### Hybridization of oligonucleotides

Complementary sequences (fw, rv) (Supplementary Tables 4 and 5) were mixed in equimolar ratio in ddH_2_O, and incubated for 5 min at 95 °C in a metal block. The samples were then allowed to cool slowly in the metal block to room temperature (RT). Concentration was determined by UV/VIS analysis, extinction coefficients and molecular mass were calculated using the IDT Oligo Analyzer (IDT). To determine the concentration of hybridized double-stranded oligonucleotides, unlike single-stranded oligonucleotides, the hypochromicity of the extinction coefficient of the double strand versus the sum of the extinction coefficients of the corresponding single strands was taken into account. Thus, the extinction coefficient *ε*_ds_ of the dsDNA is calculated from the extinction coefficients *ε*_ss_ of the single strands as follows:

1 $${\varepsilon }_{{{{{{\rm{ds}}}}}}}=\left({\varepsilon }_{{{{{{\rm{ss}}}}}},{{{{{\rm{fw}}}}}}}+{\varepsilon }_{{{{{{\rm{ss}}}}}},{{{{{\rm{rv}}}}}}}\right)\times (1-h)$$

with

2 $$h=(0059\times {fGC})+(0287\times {fAT})$$

*fGC* denotes the proportion of guanine and cytosine bases, and *fAT* the proportion of adenine and thymine bases. Efficient hybridization was verified by the change in absorption at 260 nm due to hypochromicity and non-denaturing polyacrylamide gel electrophoresis at 20% gel concentration with 1x TBE as gel- and running buffer, followed by staining with GelStar (Lonza).

### Cell culture

Cells were incubated in a 37 °C incubator with 5% CO_2_ and humidified atmosphere. Cells were counted and viability was determined using Trypan blue stain 0.4% (Invitrogen) in combination with Countess™ cell counting slides and Countess® Automated Cell Counter (Invitrogen). Adherent cells were passaged by trypsinization with 1x trypsin-EDTA solution (Thermo Fisher Scientific) in standard cell culture dishes (Sarstedt). Suspension cells were passaged by dilution in suspension cell culture dishes (Sarstedt). HEK293 (DSMZ ACC-305) and Cos7 cells (Sigma) were cultured in DMEM (Invitrogen)+10% FBS (Invitrogen) and HEK293-T cells were cultured in RPMI-1640 Medium (Sigma)+10% FBS. THP-1 cells (ATCC TIB-202) were cultured in RPMI-1640 Medium+10% FBS and rigorously maintained between 3–8 × 10^5^ cells per ml to prevent unwanted differentiation.

#### Transient transfection of HEK293 cells with Lipofectamine

For this, 3 × 10^5^ cells per ml were seeded at 2 ml per well in a standard 6w plate (Sarstedt) the day before transfection. On the day of transfection, the medium of the cells was changed. 2 µg of plasmid DNA (pAc-GFP-CbFic2 versions) as well as 6 µl of Lipofetamine2000 (Thermo Fisher Scientific) were diluted each in 150 µl of OptiMEM (Thermo Fisher Scientific). Both dilutions were combined, mixed, incubated for 5 min at RT, and added dropwise to the seeded cells. 48 h after transfection, cells were harvested and lysed with RIPA buffer (Thermo Fisher Scientific), or acid-soluble nuclear proteins were extracted.

#### Transient co-transfection of HEK293 cells with polyethyleneimine

mH2A1 (mH2A1-CT-MYC)^47^ and CbFic2 (pAc-GFP-CbFic2), or H3.1 (H3.1-CT-MYC) and CbFic2 (pAc-GFP-CbFic2), were co-transfected in HEK293 cells by a 3x excess polyethyleneimine (PEI) transfection. Then, 7 × 10^5^ cells per ml were seeded at 2 ml per well in a standard 6w plate (Sarstedt) the day before transfection. On the day of transfection, the medium of the cells was changed. Next, 6 µg of plasmid DNA, as well as 18 µg of branched PEI, M_w_ ~ 25,000 (Sigma-Aldrich), were diluted each in 150 µl of OptiMEM (Thermo Fisher Scientific). Both dilutions were combined, mixed, incubated for 5 min at RT, and added dropwise to the seeded cells. Forty-eight hours after transfection, cells were harvested and acid-soluble nuclear proteins were extracted.

#### Generation of stable THP-1 cell lines

Using lentiviruses, human monocytic THP-1 cells were transduced with a G418-selectable Tet-On® plasmid^75^ expressing doxycycline-inducible GFP-P2A-V5-CbFic2 and the respective CbFic2_E66G_ and CbFic2_H205A_ mutants. These stable monocytic cell lines were then differentiated into monocyte-derived macrophages (MDMs) using phorbol-12-myristate-13-acetate (PMA)^40^ before the induction of expression by doxycycline. The GFP-P2A sequence upstream of the CbFic2 sequence is used to rapidly follow the successful induction of expression. The P2A sequence causes ribosomes to skip synthesis of the glycyl-prolyl peptide bond at the C-terminus of the peptide^78,79^ so that GFP and the downstream CbFic2 are present separately in the cell. CbFic2 thus carries only a V5 tag at the N-terminus.

##### Virus production in HEK293-T cells

Per stable cell line (including control plasmid and control without target plasmid), 5 × 10^6^ HEK293-T cells were seeded in a 10 cm dish in THP-1 culture medium (RPMI-1640+10% FBS). One day later, HEK293-T cells were transfected for virus production using Lipofectamine2000 (Thermo Fisher Scientific) with the respective target plasmid and pack plasmids: 10 µg target plasmid (pCW57-GFP-P2A-V5-CbFic2), 10 µg pMDLg/pRRE (#12251 addgene), 5 µg pRSV-Rev (#12253 addgene), and 2 µg pMD2.G (#12259 addgene)^80^ were added to 750 µl OptiMEM (Thermo Fisher Scientific). For each batch, 40 µl Lipofectamine2000 was added to 750 µl OptiMEM and incubated for 5 min at RT. Plasmids and Lipofectamine in OptiMEM were mixed and incubated for 20 min at RT. The HEK293-T cell medium was removed, the cells were washed with DPBS (Sigma-Aldrich), and 5 ml of fresh RPMI-1640+10% FBS medium was added. The plasmid-Lipofectamine mixture was added to the cells and incubated for 3 h at 37 °C, 5% CO_2_. Then, another 5 ml of RPMI-1640+10% FBS medium was added to the cells, and the cells were cultured for 24 h.

##### Lentiviral transduction with 24, 48 and 72 h titers

Twenty-four hours later (24 h titer), 1 ml of 5 × 10^5^ THP-1 cells were freshly seeded per batch (including control plasmid and control without target plasmid) in a 6w suspension culture plate (Sarstedt). The 10 ml viral supernatant was removed from the HEK293-T cells with a syringe and aliquoted into 2 ml after passage through a sterile filter (0.45 µm). To the HEK293-T cells, 10 ml of fresh RPMI-1640+10% FBS medium was added, and the cells were further cultured for 24 h. Then, 2 ml each of the filtered virus supernatant was added to the THP-1 cells (totaling 3 ml medium per well), after which cells were spinfected at 500×*g*, RT, for 1 h before further culturing for 24 h. Then, 24 h later (48 h titer), the same procedure was repeated (5 ml medium per well in total). Another 24 h later (72 h titer), the viral supernatant was collected and filtered as described before while discarding the HEK293-T cells. THP-1 cells were counted and if >3 × 10^5^ cells per well were reached, divided into 2x 6w plates of 2.5 ml per well. For each stable cell line, 2 × 2 ml of the sterile-filtered virus supernatant was added to the THP-1 cells (total 2 × 4.5 ml per well) before spinfection for 1 h at 500×*g*, RT. After 24 h, the THP-1 cell medium was renewed by combining both wells with THP-1 cells per target plasmid (total 9 ml per target plasmid) and centrifugation at 300×*g* for 5 min at RT. The supernatant was then discarded, and 5 ml of fresh medium was added. Each batch was transferred to a T25 suspension culture bottle (Sarstedt) and incubated upright for 48 h.

##### Selection

Forty-eight hours after media renewal and 72 h after the last spinfection, cell selection was started with G418 disulfate salt (Sigma-Aldrich) at 300 ng µl^−1^ as determined by a kill curve. During this phase, cell density was maintained between 0.2 × 10^6^ and 0.8 × 10^6^ cells per ml by dilution with medium containing G418 every 2–3 days, and cell viability was monitored. No cells were discarded during selection, and the culture flask was enlarged when necessary. When the control cells without target plasmid had completely died and the stable cell lines had reached viability above 90%, a portion of each stable cell line culture medium + 5% DMSO was frozen. Successful generation of the stable cell line was confirmed by WB against the target proteins CbFic2 and GFP or microscopy against GFP after induction of expression.

#### Differentiation of THP-1 cells

THP-1 cells were seeded in 10 cm dishes (Sarstedt) at a concentration of 3 × 10^5^ cells per ml in RPMI-1640+10% FBS spiked with 25 nM PMA (Sigma-Aldrich). Cells were differentiated for 48 h before the medium was changed to PMA-free RPMI-1640+10% FBS to recover differentiated cells (adherent) and remove undifferentiated cells (non-adherent)^40^.

#### Expression of CbFic2 in stable THP-1 cells

When expression of CbFic2 in monocyte-derived macrophages (MDMs) is described, stable THP-1 cell lines were differentiated as described. If MDMs are not mentioned, undifferentiated THP-1 cells were used for expression. To induce expression, 1 µg ml^−1^ doxycycline (Sigma-Aldrich) was added to the culture medium and renewed every 24 h. Expression was monitored by fluorescence microscopy, GFP filter during the experiment.

#### *Coxiella* infection

*Coxiella burnetii* Nine Mile (NM) strain was cultured in L929 cells for 10 days. Infected cells were sonicated and centrifuged at 10,000×*g* for 10 min, then washed and stored at −80 °C. Then, 3 × 10^6^ J774 cells per well were infected with *C. burnetii* NMI (100 MOI). After 96 h of infection, infected cells were collected and centrifuged at 500×*g* for 10 min prior to cell lysis.

#### Cell lysis

Adherent cells were harvested by media removal and scraping cells in cold DPBS. Afterward, cells were treated the same as suspension cells. Cells were pelleted by centrifugation at 300×*g*, 5 min, 4 °C and washed twice with cold DPBS. The cell pellet was then resuspended in RIPA lysis and extraction buffer (Thermo Fisher Scientific) with the addition of cOmplete EDTA free protease inhibitor (Roche) and incubated for 15 min at 4 °C under rotation. The cell lysate was then centrifuged at 20,000×*g* for 15 min, and the supernatant was transferred to a new tube. The concentration was determined by Bradford assay using Protein Assay Dye Reagent Concentrate (BioRad) and Pierce™ Bovine Serum Albumin Standard Ampules (Thermo Fisher Scientific) as standard and the cell lysate flash frozen and stored at −80 °C.

#### Cell fractionation

Cells were harvested as described for cell lysis. The cell pellet was fractionated using the Subcellular Protein Fractionation Kit for Cultured Cells (Thermo Fisher Scientific) according to the manufacturer’s protocol. In short, the cell pellet was stepwise separated into cytoplasmic, membrane, nuclear soluble, chromatin-bound and cytoskeletal protein extracts. All steps were performed either at 4 °C or on ice, and Halt™ Protease Inhibitor Cocktail (Thermo Fisher Scientific) was added to all buffers. Protein content was determined by Bradford assay as described. Aliquots were flash-frozen at −80 °C.

#### Preparation of acid-soluble nuclear fraction according to abcam

HEK293 cells were counted, harvested and washed 2 times with ice-cold DPBS. Pellets were resuspended in Triton extraction buffer (TEB: DPBS, 0.5% Triton X-100 (v/v)) at a cell density of 10^7^ cells per ml. Cells were incubated on ice for 10 min under mild agitation for lysis. Samples were centrifuged at 6500×*g* for 10 min at 4 °C to pellet the nuclei, and the supernatant was discarded. Nuclei were resuspended again in half the volume of TEB and centrifuged as before. Pellets were resuspended in 0.2 N HCl at a density of 4 × 10^7^ nuclei per ml and acid-extracted overnight (oN) at 4 °C. Samples were centrifuged at 6500×*g* for 10 min at 4 °C to pellet nuclear debris. The histone-containing acidic supernatant was neutralized with 1/10 volume of 2 M NaOH, and protein content was determined by Bradford assay as described. Aliquots were frozen at −20 °C.

#### Protein localization by fluorescence microscopy

Cos7 cells were seeded on 8w µ-slides (ibidi) at a density of 2–4 × 10^4^ cells per cm^2^ and transiently transfected with plasmid DNA using Torpedo^DNA^ (ibidi) on the next day according to the manufacturer’s instructions. Then, 24 h post-transfection cells were stained with 1 µg ml^−1^ Hoechst 33342 (Thermo Fisher Scientific) in PBS for 10 min to label nuclei. After staining, cells were washed three times with PBS (Sigma Aldrich) and supplemented with phenol red-free growth medium (DMEM, Sigma Aldrich) for live cell imaging. Images were acquired with a Leica DMi8 wide-field microscope (Leica microsystems) using a 100x magnification objective and the manufacturer’s LAS X 2 software. Filters: GFP (Ex.: 450–490, Em.: 500–550), DAPI (Ex.: 325–375, Em.: 435–485). Greyscale images were transformed into RGB color images and RGB merged in green or blue, respectively, with ImageJ 1.37a. RGB images were overlaid with Photoshop Version 11.0 without any further adjustments.

#### Immunofluorescence (IF)

For immunofluorescence, THP-1 cells were seeded, differentiated, and induced for CbFic2 expression as described, in 8w µ-slides (ibidi). Afterward, the medium was removed and cells were washed with warm DPBS. Cells were then fixed for 15 min at 37 °C with warm 4% PFA in medium + 10% FCS. Fixed cells were incubated 3x for 5 min each with warm 0.1% Triton X-100 in DPBS, and then washed 3x with warm DPBS. Permeabilized cells were incubated with primary antibody 17G6 1:100 overnight at 4 °C in DPBS + 4% FCS. Cells were washed 3x for 10 min with DPBS and incubated with secondary antibody anti-mouse atto568 1:2000 in DPBS+4% FCS for 1 h at RT. Cells were then counterstained with DAPI 1:2000 in DPBS for 4 min. Cells were washed 3x for 10 min with DPBS, and stored covered with PBS protected from light at 4 °C until visualization. Cells were visualized with the EVOS M5000 Imaging System (Thermo Fisher Scientific) using the GFP (Ex. 470/ Em. 525 nm) and DAPI cubes (Ex. 357/Em. 447 nm).

#### Immunoprecipitation (IP)

##### Anti-H3 IP for WB

Histone H3 was precipitated from RIPA lysed, transiently transfected HEK293 cells or from the acid-soluble nuclear fraction of differentiated THP-1 cells expressing CbFic2 using Pierce ChIP-grade protein A/G magnetic beads (Thermo Fisher Scientific). In a total volume of 200 µl, 50 µg of RIPA lysate (HEK293) or acid-soluble nuclear fraction (THP-1) were incubated with 1 µg of anti-histone H3 antibody ab1791 (abcam) in 25 mM Tris pH 7.4, 150 mM NaCl, 1 mM EDTA, 5% glycerol oN at 4 °C before precipitation with 25 µl of equilibrated beads for 1 h at RT. The beads were then washed 3 times with buffer before eluting the AMPylated proteins with 50 µl of 1x Laemmli for 15 min at 30 °C. Each 10 µl input and unbound sample including 6x Laemmli buffer and 10 µl elution were analyzed by 15% SDS PAGE and WB as described.

##### Anti-myc IP for WB

In a total volume of 100 µl, 50 µg acid-soluble nuclear fraction from HEK293 cells co-expressing mH2A1 and CbFic2 mutants, or H3.1 mutants and CbFic2, respectively, were incubated with 2 µg of anti-myc antibody 9E10 (Santa Cruz) in 20 mM HEPES pH 7.5, 150 mM NaCl, 1 mM MgCl_2_, 20% glycerol oN at 4 °C before precipitation with 25 µl of equilibrated Pierce ChIP-grade protein A/G magnetic beads for 1 h at RT. The beads were then washed 3 times with buffer before eluting the target protein with 50 µl of 1x Laemmli for 15 min at 30 °C. Each 10 µl input and unbound sample including 6x Laemmli buffer and 10 µl elution were analyzed by 12% Bis-Tris gels and WB as described.

##### Co-IP for WB

In the co-IP, it was tested whether CbFic2 forms DNA-induced hetero-dimers of V5- and HA-tagged protein. For this purpose, 0.2 µM HA- and 0.2 µM V5-tagged CbFic2, CbFic2_S22DS26D_ or CbFic2_ΔHTH_, respectively, were incubated with 4 µM 20 bp or 40 bp dsDNA in the presence of 2 µg mouse anti-HA Tag Monoclonal Antibody (2-2.2.14) (Thermo Fisher Scientific) in a total volume of 115 µl 20 mM HEPES pH 7.0, 150 mM NaCl, 1 mM MgCl_2_, 20% (v/v) glycine, 0.1% (v/v) Tween 20 oN at 4 °C. For each CbFic2 version, a control assay without HA-tagged protein was prepared in addition, to control for unspecific binding of CbFic2. The anti-HA antibody and its bound target were then precipitated with 25 µl of equilibrated Pierce ChIP-grade protein A/G magnetic beads for 1 h at RT. The unbound sample was saved, and the beads were washed 3 times with buffer before eluting HA-tagged CbFic2 and its binding partners with 100 μl of 0.1 M glycine, pH 2.0 for 10 min. After elution, the pH was neutralized by the addition of 15 µl 1 M Tris, pH 8.5. 5x Laemmli buffer was added to bound and unbound samples, which were subsequently separated by 12% Tris-glycine gels, and detection of the potential heterodimer was performed via anti-V5 tag antibody in a Western blot analysis.

##### Anti-AMP IP for LC-MS/MS

All chemicals used in this experimental setup were proteomic grade. Only low-binding reaction tubes (Sarstedt) were used. Glycerol-free anti-AMP antibody 17G6^37^ was centrifuged for 10 min, 16,000×*g* at 4 °C. To avoid elution of the antibody with the target protein, 17G6 was covalently coupled to beads. For this, 7 mg of magnetic Dynabeads™ M-270 epoxy beads (Thermo Fisher Scientific) were coupled with 180 µg of 17G6 according to the manufacturer’s instructions of the Dynabeads™ Co-Immunoprecipitation Kit (Thermo Fisher Scientific) and stored at a concentration of 10 mg ml^−1^ of 17G6-coupled beads at 4 °C. To allow the enrichment of AMPylated proteins, competing nucleotides were removed by proteome precipitation. Then, 250 µg of differentiated THP-1 lysates after 48 h of CbFic2_E66G_ or CbFic2_H205A_ expression were methanol/chloroform precipitated in biological and technical triplicates: lysate was diluted with 3 volumes of methanol and vortexed before adding 1 volume of chloroform (Sigma-Aldrich) and vortexing the samples. Then, 3 volumes of ddH_2_O were added and the samples vortexed before centrifugation at 15,000 rpm for 2 min at 4 °C. The aqueous upper phase was carefully aspirated, 3 volumes of methanol were added, the samples vortexed before centrifugation at 15,000 rpm for 5 min at 4 °C. The supernatant was then removed and the protein pellets were air-dried. Pellets were dissolved under polymer-free conditions in a final volume of 100 µl binding buffer (25 mM Tris pH 7.4, 100 mM NaCl) and pH was adjusted to 7.5 with 5 M NaOH. Then, 100 µl of magnetic epoxy-coupled anti-AMP antibody beads per batch were washed with 900 µl of binding buffer, resuspended in 100 µl binding buffer and combined with the solubilized protein pellet. Samples were incubated oN at 4 °C under rotation and then washed 3 times with 200 µl binding buffer. The supernatant was removed and bound proteins were eluted with 120 µl 0.5 M NH_4_OH, 0.5 mM EDTA for 20 min at RT. The procedure was repeated, and the eluates combined. Elutions were dried in a SpeedVac alpha RVC (Christ) without heat and then submitted for trypsin digestion and LC-MS/MS analysis.

### Proteinchemical methods

Recombinant human histones were purchased from NEB. Rab1b_3-174aa_ was produced as previously described^81^.

#### Recombinant expression

All *E. coli* strains used for recombinant expression were obtained from in-house stocks. Chemically competent cells (Lemo21(DE3) for all CbFic2 versions and FRET control constructs; Rosetta 2 for TS-H3 peptides, BL21 (DE3) for GST-H3 peptides) were transformed with plasmid DNA (50 ng). A single colony was collected and cultured in 50 ml LB medium supplemented with the respective selection antibiotics at 37 °C oN. LB medium supplemented with the respective antibiotics was inoculated at a ratio of 1:100 (v/v) with the preculture and cultured at 37 °C, 180 rpm to an OD_600 nm_ of 0.5–0.8. Cells were cooled to 23 °C and recombinant protein expression was induced by the addition of 0.5 mM IPTG, or in the case of CyPet and YPet by the addition of 2 g L^−1^ arabinose. Cells were cultured at 180 rpm for 20 h before being harvested by centrifugation at 5000×*g*, 30 min, 4 °C. The cell pellet was washed with 1x PBS pH 7.5 before being frozen at −20 °C until further use.

#### Bacterial cell lysis

Cell pellets were thawed on ice and resuspended in 10 ml of cold buffer (20 mM HEPES pH 7.5, 500 mM NaCl, 1 mM MgCl_2_, 1 mM ß-Me for CbFic2 versions; 20 mM HEPES pH 7.5, 500 mM NaCl, 1 mM ß-Me for TS-H3 peptides and GST-H3 peptide) per 5 ml of cell pellet. Cells were disrupted with Constant Cell Disruption Unit (Constant Systems) at 2.1 kbar without any DNAse addition before the addition of 1 mM PMSF. For all CbFic2 versions encompassing the HTH domain 0.075% PEI was added dropwise under constant stirring from a 5% stock to precipitate bound DNA. Crude cell lysates were centrifuged at 48,000×*g* for 45 min, 4 °C, and the supernatant was further purified.

#### Ion metal affinity chromatography (IMAC)

The supernatant after cell disruption containing recombinant His-tagged protein was supplemented with 40 mM Imidazole and subsequently loaded onto a Ni^2+^-loaded HiTrap 5 ml chelating HP column (GE Healthcare) or Ni^2+^-loaded Bio-Scale mini Nuvia IMAC cartridge (BioRad) pre-equilibrated in buffer A (20 mM HEPES pH 7.5, 500 mM NaCl, 1 mM MgCl_2_, 1 mM ß-Me for CbFic2 versions and FRET control constructs; 20 mM HEPES pH 7.5, 500 mM NaCl, 1 mM ß-Me for TS-H3 peptides and GST-H3 peptides). The column was washed with 40 mM imidazole and His-tagged protein was eluted using a 5 ml fractional gradient of 40–350 mM imidazole over 20 column volumes. Protein-containing fractions were analyzed by SDS PAGE and pooled for TEV protease cleavage, if applicable. For GST-H3 peptides, protein-containing fractions were analyzed by SDS PAGE, combined, and concentrated as well as buffer exchanged to 20 mM HEPES pH 7.5, 100 mM NaCl, 2 mM DTT using Amicon Ultra centrifugal filter units (Merck Millipore), before being subjected to TEV cleavage.

#### TEV protease cleavage

For TEV protease cleavage of fusion tags after IMAC chromatography, 1 mg of His_6_-tagged TEV protease from in-house stock was added to every 45 mg of protein of a size of 68 kDa. The mixture was dialyzed against 5 l of TEV dialysis buffer (20 mM HEPES pH 7.5, 500 mM NaCl, 1 mM MgCl_2_, 1 mM ß-Me for all CbFic2 versions; 20 mM HEPES pH 7.5, 100 mM NaCl, 1 mM ß-Me for TS-H3 peptides) at 4 °C oN in dialysis tubing with a molecular weight cut-off (MWCO) of 12,000–14,000 Da (CbFic2 versions) or MWCO of 6000–8000 Da (TS-H3 peptides) and 29 mm diameter (Serva Electrophoresis). Afterward, the protein solution was submitted a second time to IMAC. Unlike the first run, no imidazole was added to the protein before loading, and the protein eluted in the flow-through or 40 mM imidazole wash step due to the lack of His tag, while the cleaved off fusion tag and the TEV protease bound to the column. Protein-containing fractions were analyzed by SDS PAGE, combined, and concentrated for injection onto preparative size exclusion chromatography. For H3 peptides from pGATEV, concentrated GST-H3 peptides were cleaved by TEV in a 1.5 ml reaction tube oN at 4 °C, before being centrifuged through 0.5 ml Amicon Ultra centrifugal filter units (Merck Millipore) with a MWCO of 10 kDa. The peptide containing flow-through was collected, and concentration determined by UV/Vis analysis at 205 nm with an extinction coefficient of ε^1 mg ml^^−1^ = 31^82^.

#### Preparative size exclusion chromatography (SEC)

After the previous IMAC, the protein solution was concentrated to less than 2 ml using Amicon Ultra centrifugal filter units (Merck Millipore) with appropriate MWCO, centrifuged for 5 min at 20,000 rpm, 4 °C, and subjected to size exclusion chromatography on a HiLoad™ 16/600 Superdex™ 75 pg column (GE Healthcare) using SEC buffer (20 mM HEPES pH 7.5, 500 mM NaCl, 1 mM MgCl_2_, 1 mM DTT for all CbFic2 versions and FRET control constructs; 20 mM HEPES pH 7.5, 100 mM NaCl for TS-H3 peptides). The run was collected into 2 ml fractions. Fractions were analyzed by SDS PAGE, target protein-containing samples were pooled, concentrated to approximately 10 mg ml^−1^ using Amicon Ultra centrifugal filter units (Merck Millipore) with appropriate MWCO, frozen in liquid nitrogen, and stored at −80 °C.

#### Preparative TS-H3_1-36aa_-AMPylation and CbFic2-AMPylation

For TS-H3_1-36aa_-AMP, 100 µM TS-H3_1-36aa_ were incubated with 10 µM TS-CbFic2_E66G_, 10 µM 20 bp dsDNA, 2.5 mM ATP in 20 mM HEPES pH 7.5, 50 mM NaCl, 5 mM MgCl2, 2 mM DTT oN at 30 °C. TS-H3_1-36aa_-AMP was concentrated using Amicon Ultra centrifugal filter units (Merck Millipore) and purified via SEC with a Superdex 10/300 75 pg column (GE Healthcare) coupled to a Prominence HPLC system (Shimadzu) in 20 mM HEPES pH 7.5, 100 mM NaCl. Protein concentration was determined by Bradford assay as described, using TS-H3_1-36aa_ as standard. For CbFic2_E66G_-AMP, 5 µM CbFic2_E66G_ were incubated with 1 mM ATP in 20 mM HEPES pH 7.5, 150 mM NaCl, 1 mM MgCl2, 2 mM DTT oN at 30 °C. CbFic2_E66G_-AMP was concentrated using Amicon Ultra centrifugal filter units (Merck Millipore) and buffer exchanged 5 times. Protein concentration was determined by Bradford assay as described. In both cases, AMPylation was confirmed by MS.

### Analytical methods

#### SDS PAGE analysis

For Tris-Glycine and Bis-Tris gels, samples were dissolved in SDS-PAGE sample buffer (Laemmli) (62 mM Tris-HCL, pH 6.8, 2% (w/v) SDS, 10% (v/v) glycerol, 5% (v/v) ß-Me, 0.001% (w/v) bromophenol blue) and heated to 95 °C for 5 min. For Tris-Tricine separation, samples were dissolved in 50 mM Tris-HCl pH 6.8, 10% (v/v) glycerol, 0.5% (w/v) SDS, 0.01% (w/v) Serva G-250, 0.5% (v/v) ß-Mercaptoethanol. For WB analysis of cell lysates, 20 μg total protein, for acid-soluble nuclear fraction, 10 μg was applied; 1–5 μl Color Prestained Protein Standard, Broad Range (11–245 kDa or 10–250 kDa) (NEB) was used as a standard.

##### Tris-Glycine gels

Gels were cast using 4x stacking gel buffer (0.5 M Tris-HCl pH 6.8, 0.4% (w/v) SDS), 4x separation gel buffer (1.5 M Tris-HCl pH 8.8, 0.4% (w/v) SDS), an acrylamide/bis solution 37.5:1 (30% w/v); 2.6% crosslinker (Serva) and polymerization starters 200 × 10% (w/v) ammonium persulfate (APS) and 2000x tetramethylethylenediamine (TEMED) (VWR International) poured into a Mini Protean™ Tetra System (BioRad). 12% or 15% Tris-glycine gels were run at RT at 60 mA per gel (buffer: 25 mM Tris, 0.1% (w/v) SDS, 192 mM glycine).

##### Bis-Tris gels

Gels were cast using 3x gel buffer (1 M Bis-Tris, Sigma-Aldrich), an acrylamide/Bis solution 37.5:1 (30% w/v); 2.6% crosslinker (Serva) and polymerization starters 200 × 10% (w/v) APS and 2000x TEMED (VWR) in a Mini Protean™ Tetra System (BioRad). 10%, 12% or 15% Bis-Tris gels were run at RT in 1x low-MW running buffer (50 mM MES, 50 mM Tris base, 1 mM EDTA, 0.1% SDS) supplemented with 5 mM sodium bisulfite at 30 mA per gel.

##### Tris-Tricine gels

Gels were cast using 3x gel buffer (3 M Tris-base, 0.3% (w/v) SDS, pH 8.45), an acrylamide/Bis solution 37.5:1 (30% w/v); 2.6% crosslinker (Serva) and polymerization starters 200 × 10% (w/v) APS and 2000x TEMED (VWR) in a Mini Protean™ Tetra System (BioRad). 16.5% Tris-Tricine gels were run at RT in 1x Tris-Tricine running buffer (100 mM Tris base, 100 mM Tricine, 0.1% (w/v) SDS) at 60 mA per gel.

After separation, gels were subjected to WB or protein bands were directly visualized using Coomassie staining: Gels were soaked in 0.15% (w/v) Coomassie blue R250, 44% (v/v) ethanol, 12% (v/v) acetic acid for 10 min before destaining with 10% (v/v) acetic acid.

#### Western blot analysis

For this, 50–200 ng of recombinant protein as indicated or 20 μg of cell lysate or 10 μg of acid-soluble nuclear fraction, respectively, were separated by SDS-PAGE as indicated. The gel was transferred as a sandwich with Whatman paper and a transfer buffer of 48 mM Tris, 39 mM glycine, 1.3 mM SDS, 20% methanol onto a MeOH-activated Immobilon®-P membrane (Merck Millipore). In the case of all recombinant histone and TS-H3 peptide analyses, Immobilon®-PSQ membrane (Merck Millipore) was used. For blotting, a constant current of 0.7 mA cm^–2^ was applied for 2 h to the semi-dry blotter V20-SDP (SCIE-PLAS, Cambourne, United Kingdom). After blotting, the PVDF membrane was blocked with 1x Roti® block (Carl Roth, Karlsruhe, Germany) in Tris-buffered saline containing 0.1% Tween20 (TBS-T) for 1 h. The primary antibody was then added to the blocking solution and incubated oN at 4 °C. The membrane was then washed 3 times with TBS-T for 10 min, followed by incubation with a secondary antibody-peroxidase conjugate in TBS-T for 1 h. Again, the membrane was washed 3 times for 10 min in TBS-T before the peroxidase signal was developed using the SuperSignal™ West Dura (Thermo Fisher Scientific) and chemiluminescence was detected using the Intas ECL Chemocam (Intas Science Imaging Instruments, Göttingen, Germany). Mouse anti-AMP monoclonal antibody 17G6^37^ was used 1:1000 (0.5 μg ml^−1^) in the presence of 1 mM MnCl_2_. Mouse anti-GAPDH monoclonal antibody sc-47724 (Santa Cruz Biotechnology, Dallas, Texas) was used 1:1000. Rabbit polyclonal anti-histone H3 antibody ab1791 (Abcam) was used 1:5000. Polyclonal rabbit anti-GRP78/BiP antibody PA5-34941 (Thermo Fisher Scientific) was used 1:5000. Chicken anti-CbFic2 antibody (3.5 mg ml^−1^) (AG Kaspers, LMU, Munich, Germany) was used 1:1000. Rabbit anti-GFP Polyclonal Antibody A111-22 (Thermo Fisher Scientific) was used 1:2000. Mouse anti-V5 Tag Monoclonal Antibody (TCM5), eBioscience™ 14-6796-82 (Thermo Fisher Scientific) was used 1:1000. Mouse anti-HA Tag Monoclonal Antibody (2-2.2.14) (Thermo Fisher Scientific) was used 1:5000. HisProbe™-HRP-conjugate 15165 (Thermo Fisher Scientific) was used according to the manufacturer’s protocol. Secondary goat anti-mouse IgG (H+L) HRP conjugate 31430 (Thermo Fisher Scientific) was used at a ratio of 1:20,000. Secondary goat anti-rabbit IgG H&L (HRP) preadsorbed ab7090 (abcam) was used at a ratio of 1:20,000. Secondary rabbit anti-chicken IgY (IgG) (H+L) HRP conjugate 303-035-003 (Jackson ImmunoResearch Laboratories) was used 1:5000. For reprobing, WB membranes were stripped in ready-to-use Roti®Free Stripping-Buffer (Carl Roth) for 5 min at 60 °C in a water bath, before being washed 6 × 10 min in TBS-T at RT. Afterward, membranes were re-blocked and probed as described above. All brightness and contrast changes were applied uniformly to the entire image. Clipping, e.g., due to different molecular weights, is indicated by a break in the image frame.

#### Assays for analysis by WB

Recombinant Histone AMPylation by CbFic2: 0.1 mg ml^−1^ recombinant histones (NEB) were incubated with 0.2 µM CbFic2_E66G_ or CbFic2_H205A_, respectively, in the presence of 1 mM ATP in a buffer of 20 mM HEPES pH 7.5, 50 mM NaCl, 1 mM MgCl_2_, 1 mM TCEP, 1x cOmplete™ EDTA-free protease inhibitor (Roche) at 23 °C for 20 h. Then, 50 ng histones were run on 15% Laemmli gels and blotted on 0.22 µm PVDF. For loading controls, 1 µg of histones were run on 15% Laemmli gels and stained with Coomassie.

Mutational analysis of TS-H3_1-20aa_ and TS-H3_1-36aa_ AMPylation: 1 mg ml^−1^ TS-H3_1-20aa_ and its mutants TS-H3_1-20aa T3A_, TS-H3_1-20aa T6A_, TS-H3_1-20aa S10A_, TS-H3_1-20aa T11A_ were incubated with 1 µM CbFic2_E66G_ or CbFic2_H205A_, respectively, and TS-H3_1-36aa_ and its mutants TS-H3_1-36aa S10A_, TS-H3_1-36aa S28A_, TS-H3_1-36aa S10A S28A_ were incubated with 5 µM CbFic2_E66G_ or CbFic2_H205A_, respectively, in the presence of 5 µM 20 bp dsDNA, 2.5 mM ATP in 20 mM HEPES pH 7.5, 50 mM NaCl, 5 mM MgCl_2_, 1 mM DTT, 10% glycerol for 30 °C oN. 100 ng peptides were run on 16.5% Tris-Tricine gels and blotted on 0.22 µm PVDF membranes. For loading controls, 1 µg peptides were run on 16.5% Tris-Tricine gels and Coomassie stained.

TS-H3_1-36aa_ (de)AMPylation by different CbFic2 mutants: For AMPylation, 50 µM TS-H3_1-36aa_ were incubated with 1 µM CbFic2_E66G_, CbFic2_E66G ΔHTH_, CbFic2, CbFic2_ΔHTH_, CbFic2_S22D S26D_ and CbFic2_S22D S26D ΔHTH_ in the presence or absence of 4 µM 20 bp dsDNA, 1 mM ATP in 20 mM HEPES pH 7.5, 50 mM NaCl, 1 mM MgCl_2_, 1 mM TCEP for 8 h at 37°. For deAMPylation 50 µM TS-H3_1-36aa_–AMP were incubated with 1 µM CbFic2_E66G_, CbFic2_E66G ΔHTH_, CbFic2, CbFic2_ΔHTH_, CbFic2_S22D S26D_ and CbFic2_S22D S26D ΔHTH_ in the presence or absence of 4 µM 20 bp dsDNA in 20 mM HEPES pH 7.5, 50 mM NaCl, 1 mM MgCl_2_, 1 mM TCEP for 8 h at 37°. Then, 100 ng peptides were run on 16.5% Tris-Tricine gels and blotted on 0.22 µm PVDF membranes. For loading controls, 1 µg peptides were run on 16.5% Tris-Tricine gels and Coomassie stained.

CbFic2 co-incubation for cis/trans auto-AMPylation: WB analysis of auto-AMPylation of CbFic2 in cis/trans. 0.3 µM CbFic2 versions as indicated were incubated alone or in the presence of another CbFic2 version, in the presence or absence of 2.5 µM 20 bp dsDNA, in the presence of 1 mM ATP in a buffer of 20 mM HEPES pH 7.5, 150 mM NaCl, 1 mM MgCl_2_, 1 mM TCEP for 8 h at 37 °C. Then, 50 ng protein was run on 12% Laemmli gels and blotted on 0.45 µm PVDF.

CbFic2 concentration-dependent auto-AMPylation: From a starting concentration of 50 µM CbFic2 versions as indicated, protein was diluted to 15 µM, 5 µM, 1.5 µM, 0.5 µM and 0.15 µM, and incubated in the presence or absence of 50 µM, 15 µM or 5 µM or 4 µM (for protein concentrations of or below 1.5 µM) 20 bp dsDNA, respectively, in the presence of 1 mM ATP in a buffer of 20 mM HEPES pH 7.5, 150 mM NaCl, 1 mM MgCl_2_, 1 mM TCEP for 8 h at 37 °C. 50 ng protein were diluted in Laemmli sample buffer, run on 12% Laemmli gels, and blotted on 0.45 µm PVDF.

##### H3.1 FL AMPylation by CbFic2 versions

0.1 mg ml^−1^ Histone H3.1 was incubated with 5 µM, 0.5 µM or 0.1 µM of CbFic2_E66G_ or CbFic2_E66G ΔHTH_ with or without 5 µM 20 bp dsDNA in the presence of 1 mM ATP, or, 0.1 mg ml^−1^ Histone H3.1 was incubated with 0.5 µM of CbFic2_E66G_, CbFic2_E66G ΔHTH_, CbFic2, CbFic2_ΔHTH_, and CbFic2_S22D S26D_ with or without 5 µM 20 bp dsDNA in the presence of 1 mM ATP in a buffer of 20 mM HEPES pH 7.5, 50 mM NaCl, 1 mM MgCl_2_, 1 mM TCEP, 1x cOmplete™ EDTA-free protease inhibitor (Roche) at 37 °C for 8 h. Then, 100 ng Histone H3.1 was diluted in Tris-Tricine sample buffer, run on 16.5% Tris-Tricine gels and blotted on 0.22 µm PVDF.

#### B–Z transition by circular dichroism

B–Z transition of DNA duplex was monitored as described previously^58,83^. An oligonucleotide of d(CG)_10_ (IDT) or 20 bp (40% GC content) (IDT) was dissolved in CD1-buffer (5 mM HEPES, pH 7.0, 10 mM NaCl) and hybridized prior to use. Protein was dialyzed against CD1-buffer for 20 h at 4 °C prior to use. 1 μM of dsDNA was dissolved in CD1-buffer and mixed with CbFic2 to final concentrations of 1 μM ([P]/[N] = 1), 2 μM ([P]/[N] = 2) and 4 μM ([P]/[N] = 4). [P] and [N] stand for protein concentration and DNA concentration, respectively. As control, dsDNA was measured in 5 mM HEPES, pH 7.0, 4 M NaCl, and 75% ethanol. Before each measurement, samples were incubated for 1 h at 21 °C. CD spectra were recorded at 21 °C on a Chirascan CD Spectrometer (Applied Photophysics, Leatherhead, Surrey, UK) between 230 and 320 nm using a 0.5 cm quartz cuvette. Machine settings were as follows: 1 nm bandwidth, 1 s response and 1 nm data pitch. Spectra were background subtracted and visualized using GraphPad Prism 8.0. Each curve represents the mean of three separate measurements.

#### Fluorescence anisotropy

Concentrations of all proteins were determined by Bradford as described. Per measurement, all data points were pipetted in technical triplicates into black low-binding, flat-bottom 384w plates (Greiner Bio-One) using the epMotion 5075t pipetting robot (Eppendorf). Per well, 20 µl of 1 nM labeled ligand in 20 mM HEPES pH 7.5, 150 mM NaCl, 1 mM MgCl_2_, 1 mM TCEP were pipetted. 20 µM or 50 µM of the indicated CbFic2 version in the same buffer served as the starting point of a dilution series by a factor of 0.75 over 31 data points. The last data point corresponds to buffer without protein. For each triplicate, 10 µl of the dilution series were added to the ligand. The plate was incubated at 25 °C for 10 min before fluorescence anisotropy was measured in a Spark plate reader (Tecan) after 10 s of orbital shaking with excitation at 485 nm, bandwidth 20 nm and emission at 535 nm, bandwidth 25 nm, gain 70 at 25 °C. Technical triplicates were averaged and the delta anisotropy was calculated by subtracting protein-free anisotropy from the anisotropy at highest protein concentration. The standard deviation was mapped as error bars. Data were blotted and fitted using the „*Specific binding with Hill slope*” model (3) in GraphPad Prism 8.0:

3 $$Y=\frac{{B}_{\max }\times {X}^{h}}{{{K}_{{{{{{\rm{D}}}}}}}}^{h}+{X}^{h}}$$

with *B*_max_ as the maximum of specific binding in the same unit as *Y*, *K*_D_ as the concentration of the ligand at half-maximum binding in the same unit as *X*, *h* as the hill slope and indicator of cooperativity. If DNA was used as ligand, 5‘-fluoresceine isothiocyanate (FITC) fluorescent oligonucleotides were hybridized with their respective non-fluorescent reverse complement oligonucleotide (Supplementary Table 5) as described. For ATP binding measurements, N6-(6-Aminohexyl)-ATP-5-FAM (Jena Bioscience) was applied as ligand.

#### Solution based FP-FRET

Time course measurements were performed using a Jasco FP-8300 Spectrofluorometer. The measurement was started with 700 µl of filtered and degassed buffer (20 mM HEPES pH 7.5, 150 mM NaCl, 1 mM MgCl_2_, 1 mM TCEP) in a stirred quartz cuvette at 25 °C. After 3 min, CyPet-CbFic2 or its mutants (donor) and after another 10 min, YPet-CbFic2 or its mutants (acceptor) were added at concentrations of 0.2 µM, 0.5 µM, 1 µM or 2 µM as indicated (resulting in total CbFic2 concentrations of 0.4 µM, 1 µM, 2 µM or 4 µM, respectively). After another 10 min incubation, 4 µM of the indicated dsDNA was added three times in succession, with each incubation lasting 10 min. For the control measurements, free CyPet and YPet were added in the order described, but DNA was added only twice before a CyPet-YPet fusion protein was added at the same concentration as donor and acceptor alone as a positive control for maximum FRET signal. Measurements were performed at 25 °C, with an excitation wavelength of 405 nm, bandwidth 2.5 nm, and an emission wavelength of 530 nm, bandwidth 5 nm, a response time of 1 s, a data interval of 1 s and medium sensitivity (with the exception of low sensitivity for the control measurement at 2 µM). Data were collected with the Spectra Time Course Measurement and smoothed by the means-movement method using a convolution width of 11 with Spectra Analysis Version 2.15.18 within the Spectra Manager Version 2.15.01 (JASCO Corporation). Intensities were normalized to the value at 760 s corresponding to the endpoint intensity of donor addition and visualized using GraphPad Prism 8.0.

#### Analytical size exclusion chromatography

In 100 μl, CbFic2, CbFic2_ΔHTH_, CbFic2_S22D S26D_, CbFic2_S22D S26D ΔHTH_ or CbFic2_E66G_ were diluted to 40 µM, 20 µM, 10 µM, 5 µM or 2.5 µM in running buffer including 12 μM vitamin B12 as an internal standard. Then, 90 μl of the sample was injected onto a Superdex 10/300 75 pg column (GE Healthcare) coupled to a Prominence HPLC system (Shimadzu, Kyōto, Japan) and run at 0.5 ml min^−1^ for 60 min in 20 mM HEPES pH 7.5, 150 mM NaCl, 1 mM MgCl_2_, 1 mM TCEP, or in 20 mM HEPES pH 7.5, 500 mM NaCl, 1 mM MgCl_2_, 1 mM TCEP or in 20 mM HEPES pH 7.5, 150 mM NaCl, 1 mM MgCl_2_, 200 mM arginine, 1 mM TCEP. Protein retention times were detected at 280 nm (A280 nm). Intensities were normalized to the vitamin B12 peak intensity and data were visualized using GraphPad Prism 8.0. Gel Filtration Standard (BioRad) comprising bovine thyroglobulin (MW 670 kDa), bovine γ-globulin (MW 158 kDa), chicken ovalbumin (MW 44 kDa), horse myoglobin (MW 17 kDa) and Vitamin B12 (MW 1.35 kDa) was used to calculate the molecular weight of the analyte.

#### Thermal shift assay (TSA)

For DNA binding, 4 µg (4 µM) TS-CbFic2_E66G_ or 2 µg Rab1b_3-174aa_ (ctrl) in 20 mM HEPES pH 7.0, 50 mM NaCl, 1 mM MgCl_2_, 2 mM DTT supplemented with 5x SYPRO® Orange (Sigma Aldrich) were measured in the presence or absence of 4 µM 20 bp dsDNA or 4 µM TS-H3_1-36aa_ as indicated. Samples were cleared from aggregates by centrifugation, and measurements were done in technical triplicates in sealed 0.2 ml 96 well PCR plates (Sarstedt) in a total volume of 20 µl. Samples were heated in the Mx3000P Real Time PCR Cycler (Agilent Technologies, Santa Clara, USA) with a heating profile of 25–95 °C at a rate of 1 °C min^−1^ and fluorescence was excited at 465 nm and emission was measured at 590 nm. The melting temperature T_M_, as the inflection point of fluorescence increase during thermal protein unfolding, was determined at the zero point of the second derivative of each melting curve. Each condition was plotted as mean value with standard deviation as error bars using GraphPad Prism 8.0.

#### Assays for analysis by mass spectrometry

Intact LC-MS analysis of TS-H3_1-36aa_ (de)AMPylation: For time-resolved observation of TS-H3_1-36aa_ AMPylation by CbFic2_E66G_, 50 µM TS-H3_1-36aa_ was incubated with 5 µM CbFic2_E66G_, 5 µM dsDNA and 1 mM ATP in 20 mM HEPES pH 7.4, 150 mM NaCl, 1 mM MgCl_2_, 1 mM DTT at 37 °C. Different dsDNA constructs were used as indicated. For time-resolved observation of TS-H3_1-36aa_-AMP deAMPylation by CbFic2, CbFic2_S22D S26_ and CbFic2_ΔHTH_, 50 µM TS-H3_1-36aa_-AMP were incubated with 0.5 µM CbFic2 in 20 mM HEPES pH 7.4, 150 mM NaCl, 1 mM MgCl_2_, 1 mM DTT at 37 °C. 5 µM 20 bp dsDNA were added as indicated. After sample collection, CbFic2 was inactivated by heat (70 °C, 10 min), centrifuged at 21,000×*g*, 4 °C for 5 min, and the supernatant analyzed by LC-MS. Samples were analyzed with an amaZon speed ESI-LCMS (Bruker Daltonics, Billerica, USA) coupled to an Ultimate 3000 UHPLC (Thermo Fisher Scientific) using a ProSwift™ RP-4H 1 ×50 mm column (Thermo Fisher Scientific). Data were evaluated using DataAnalysis (Version 5.1, Bruker Daltonics). The degree of automodification was detected by the specific mass gain of AMPylation of 329 Da. AMPylation was quantified by the proportion of the sum of the signal intensity of all AMPylated signals to the total intensity of all TS-H3_1-36aa_ signals. Measurements were performed in biological triplicates. Data were processed using GraphPad Prism 8.0 and represent the mean with standard deviation as error bars.

##### Intact LC-MS analysis of CbFic2 auto-AMPylation

In 100 µl total volume, 0.2 mg ml^−1^ (approximately 4.5 µM) of CbFic2 or CbFic2_E66G_ were incubated in 20 mM HEPES pH 7.5, 150 mM NaCl, 1 mM MgCl_2_, 1 mM TCEP, 1 mM ATP each in the presence and absence of 5 µM 20 bp dsDNA at 37 °C in the autosampler of LC-MS. Samples were analyzed hourly with maXis II ETD ESI-qTOF LC-MS (Bruker Daltonics) coupled to Elute UHPLC (Bruker) using a ProSwift™ RP-4H 1 ×50 mm column (Thermo Fisher Scientific). Data were evaluated using DataAnalysis (Version 5.1, Bruker Daltonics). The degree of automodification was detected by the specific mass gain of AMPylation of 329 Da. AMPylation was quantified by the proportion of the sum of the signal intensity of all AMPylated signals to the total intensity of all CbFic2 signals. Measurements were performed in biological triplicates. Data were processed using GraphPad Prism 8.0 and represent the mean with standard deviation as error bars.

##### LC-MS/MS identification of AMP modification site

For initial AMP modification site identification 100 µM synthetic histone H3_1-20_ peptide (Anaspec) incubated with 5 µM CbFic2_E66G_ in the presence of 2.5 mM ATP in 20 mM NaH_2_PO_4_/Na_2_HPO_4_ pH 7.0, 100 mM NaCl, 5 mM MgCl2, 1 mM DTE, 10% glycerol was subjected to LC-MS/MS analysis on an Orbitrap Fusion instrument coupled to an Ultimate3000 Nano-HPLC via a nano flex electrospray source (all Thermo Fisher Scientific). The sample was measured with a proteomic setup consisting of a 2 cm PepMap RSLC C18 trap column for desalting and a 15 cm PepMap RSLC C18 column (both columns particles 2 µm, 100 A, inner diameter 75 µm, Thermo Fisher Scientific). Separation was performed during a 33 min gradient from 3–13% (10 min 3%, 33 min 3–13%, 2 min 13–40%, 0.1 min to 90%) acetonitrile, 0.1% formic acid (FA). The column oven was set to 40 °C. Survey scans (m/z 300–1700) were acquired in the orbitrap with a resolution of 120,000 at m/z 200 and the maximum injection time set to 50 ms (target value 4·10^5^). Most intense ions of charge states 2–7 were selected for fragmentation with high-energy collisional dissociation at a collision energy of 27%. The instrument was operated in top speed mode and spectra acquired in the ion trap with the maximum injection time set to 40 ms (target value 1·10^4^). Detection of single charged product ions of m/z 136.062 (adenine), 250.09 (adenosine) or 348.07 (phosphoadenosine) triggered for refragmentation of the precursor with electron transfer dissociation (ETD), while the highest charge states were prioritized over most intense ions of precursors. The option to use calibrated charge-dependent ETD parameters was enabled and the maximum injection time was set to 40 ms (target value 5·10^4^). Data were acquired using Xcalibur software version 3.0sp2 (Thermo Scientific). The MS raw file was analyzed with MaxQuant software (version 1.5.3.8)^84^ and the peptide sequence ARTKQTARKSTGGKAPRKQL used for the implemented Andromeda search engine.

##### LC-MS/MS analysis of anti-AMP IP

After anti-AMP IP from cell lysates, all samples were dissolved in 0.1 M TEAB with 1% SDC and heated for 5 min at 95 °C for protein denaturation. Disulfide bonds were reduced, using 10 mM DTT for 30 min at 60 °C. Alkylation of free thiol groups was achieved with 20 mM iodoacetamide (IAA) for 30 min at 37 °C in the dark. For tryptic digestion, 250 ng protein was used. Digestion was performed at 37 °C oN. SDC was precipitated by 1% FA. The supernatant was dried in the vacuum concentrator SpeedVac SC110 Savant (Thermo Fisher Scientific, Bremen, Germany) and stored at −80 °C until further usage. Directly prior to LC-MS analysis, samples were resolved in 10 µl 0.1% FA. Then, 1 µl was injected into a nanoACQUITY Ultra-Performance Liquid Chromatography (UPLC) system (Waters, Milford, MA, USA). Chromatographic separation of peptides was achieved with a two-buffer system (buffer A: 0.1% FA in water, buffer B: 0.1% FA in acetonitrile). Attached to the UPLC was a peptide trap (180 μm × 20 mm, 100 Å pore size, 5 μm particle size, Symmetry C18, Waters) for online desalting and purification followed by a 25 cm C18 reversed-phase column (75 μm × 200 mm, 130 Å pore size, 1.7 μm particle size, Peptide BEH C18, Waters). Peptides were separated using an 80 min method with linearly increasing acetonitrile concentration from 2% to 35% acetonitrile in 60 min. Eluting peptides were analyzed on a Quadrupole Orbitrap hybrid mass spectrometer (QExactive, Thermo Fisher Scientific, Bremen, Germany). Here, the top 12 ions (intensity) per precursor scan (AGC Target:1 × 106 ions; Resolution: 70,000 at 200 m/z; Fill time:240 ms) were analyzed by MS/MS (Higher-energy collisional dissociation (HCD): 25 NES; AGC Target:1 × 105 ions; Resolution: 17,500 at 200 m/z; Fill time: 50 ms; Mass range: 400–1200 m/z; Dynamic precursor exclusion: 20 s). LC-MS/MS data were searched with the Sequest algorithm integrated in the Proteome Discoverer software (v 2.41.15, Thermo Fisher Scientific) against a reviewed human Swissprot database, obtained in April 2020, containing 20365 entries. Carbamidomethylation was set as fixed modification for cysteine residues (+57.021 Da). The oxidation of methionine (+15.995 Da), pyro-glutamate formation at glutamine residues and the peptide N-terminus (+17.027 Da), acetylation of the protein N-terminus (+42.001 Da) and the AMPylation of serine, tyrosine and threonine residues (+329.053 Da) were allowed as variable modifications. A maximum number of 2 missing tryptic cleavages was allowed. Peptides between 6 and 144 amino acids where considered. A precursor mass tolerance of 10 ppm and a fragment mass tolerance of 0.02 Da were allowed. A strict cutoff (false discovery rate (FDR) < 0.01) was set for peptide and protein identification. Protein quantification was carried out, using the Minora Algorithm, implemented in Proteome Discoverer. For statistical analysis protein abundance values were log2 transformed and median normalized across columns to compensate for injection amount differences. Statistical testing was carried out, using the Perseus software (Max Plank Institute for Biochemistry, Version 1.5.8.5). Proteins, identified with a *p*-value < 0.05 and at least 2 times higher abundance in AMP pulldowns were considered further. The data have been deposited to the ProteomeXchange Consortium^85^ via the PRIDE^86^ partner repository with the dataset identifier PXD040330.

#### Protein crystallization and structure determination

Sitting drop crystallization trials were carried out at 19°C, by mixing equal volumes (0.1 µL) of reservoir solution and the 6 mg mL^−1^ protein solution. Crystals grew in a condition containing TRIS 0.1 M pH 8.5 and PEG1000 20% (w/v). Crystals were soaked in cryo-solutions containing the crystallization mother liquor supplemented with 22% [v/v] glycerol, mounted onto a cryoloop (Hampton Research), and immediately flash-cooled in liquid nitrogen. Diffraction data were collected at EMBL beamline P13 at the PETRA III storage ring (DESY, Hamburg, Germany). Diffraction data were processed using XDS^87^ and scaled with Aimless from the CCP4 suite^88,89^. The structure was solved by molecular replacement using the Auto-Rickshaw automatic procedure^90^. During the workflow, the program MoRDa^91^ used search models based on a homologous Fic structure (PDB code 4RGL) to solve the structure by molecular replacement. The initial solution was further constructed and partially refined respectively with Buccaneer^92^ and Refmac^93^. The automatically built model was then corrected and further built manually with COOT^94^ and refined using the PHENIX suite^95^. The quality of the final model was assessed using the wwPDB validation server^96^ and MolProbity^97^. Structures were visualized and superimposed using The PyMOL Molecular Graphics System, Version 2.3.2 Schrödinger, LLC. X-ray data collection and refinement statistics are listed in Table 1.

**Table 1:** X-ray data collection and refinement statistics.

	CbFic2
**Crystal parameters**	
Space group	P2_1_
Cell constants	a = 69.62 Å, b = 124.38 Å, c = 71.64 Å α = 90°, β = 98.13°, γ = 90°
Subunits/asymmetric unit	2
**Data collection**	
Beamline	P13, EMBL, DESY
Wavelength (Å)	0.97625
Resolution range (Å)^a^	49.02–1.98
No. observations	357,827
No. unique reflections	83,795
Multiplicity^a^	4.3 (4.1)
Completeness (%)^a^	99.9 (100)
R_merge_ (%)^a, b^	0.135 (0.894)
CC_1/2_^a^	0.98 (0.65)
I/σ (I)^a^	5.3 (1.4)
**Refinement**	
Resolution range (Å)	46.76–1.98 (2.05–1.98)
No. refl. working set	83,700
No. refl. test set	4044
No. non-hydrogen atoms	6074
Protein	5849
Solvent	225
R_work_/R_free_ (%)^c^	18.7 (20.8)
r.m.s.d. bond (Å)/angle (°)^d^	0.17/1.40
Average B-factors (Å^2^)	56.9
Protein	57.0
Solvent	55.9
Ramachandran Plot (%)^e^	99.01/0.85/0.14
PDB accession code	8CIL

^a^The values in parentheses correspond to the highest resolution shell.

^b^R_merge_(I) = Σ_hkl_Σ_j_ | I(hkl)_j_ - <I(hkl)> | / Σ_hkl_ Σ_j_ I(hkl)_j_, where I(hkl)_j_ is the j^th^ measurement of the intensity of reflection hkl and <I(hkl)> is the average intensity.

^c^R = Σ_hkl_ | |F_obs_| - |F_calc_| |/Σ_hkl_ |Fobs|, where R_free_ is calculated without a sigma cut-off for a randomly chosen 5% of reflections, which were not used for structure refinement, and R_work_ is calculated for the remaining reflections.

^d^Deviations from ideal bond lengths/angles.

^e^Percentage of residues in favored region/allowed region/outlier region.

#### AlphaFold protein modeling

CbFic2 protein structure and dimer prediction were generated by ColabFold: AlphaFold2 using MMseqs2^98,99^. Structures were visualized and superimposed using The PyMOL Molecular Graphics System, Version 2.3.2 Schrödinger, LLC.

### Reporting summary

Further information on research design is available in the Nature Portfolio Reporting Summary linked to this article.

### Supplementary information

Supplementary Information

Description of Additional Supplementary Files

Supplementary Data

Reporting Summary

## Data Availability

Plasmids generated in this study are available upon request. Reasonable requests for the monoclonal anti-AMP antibody^37^ as well as the anti-CbFic2 antibody will be fulfilled by the corresponding author, A.I. Further information and requests for resources and reagents should be directed to and will be fulfilled by the corresponding author, A.I. (a.itzen@uke.de). This study did not generate code. Structure factors and model coordinates have been deposited in the RCSB PDB under the accession code 8CIL. The mass spectrometry proteomics data have been deposited to the ProteomeXchange Consortium^85^ via the PRIDE^86^ partner repository with the dataset identifier PXD040330. All data supporting the findings of this study are available within the paper and its Supplementary Information. Should any raw data files be needed in another format they are available from the corresponding author upon reasonable request. Source data behind graphs and all generated AlphaFold models, as well as all raw images behind Western Blot and gel depictions are deposited in the Supplementary Data file.
