# Supplementary material for: The DNA-binding induced (de)AMPylation activity of a Coxiella burnetii Fic enzyme targets Histone H3
Source: Commun Biol. 2023 Nov 6;6:1124. doi: 10.1038/s42003-023-05494-7 (PMC10628234; doi:10.1038/s42003-023-05494-7)
Supplement: Supplementary file 1 — Supplementary Information [file 42003_2023_5494_MOESM1_ESM.pdf]

# Supplemental Information

## The DNA-binding induced (de)AMPylation activity of a *Coxiella burnetii* Fic enzyme targets Histone H3

**Table of contents**


**Supplemental Figures**

Supplemental Figures S1-S5

**Supplemental Tables**

Supplemental Tables 1-5

## Supplemental Figures:

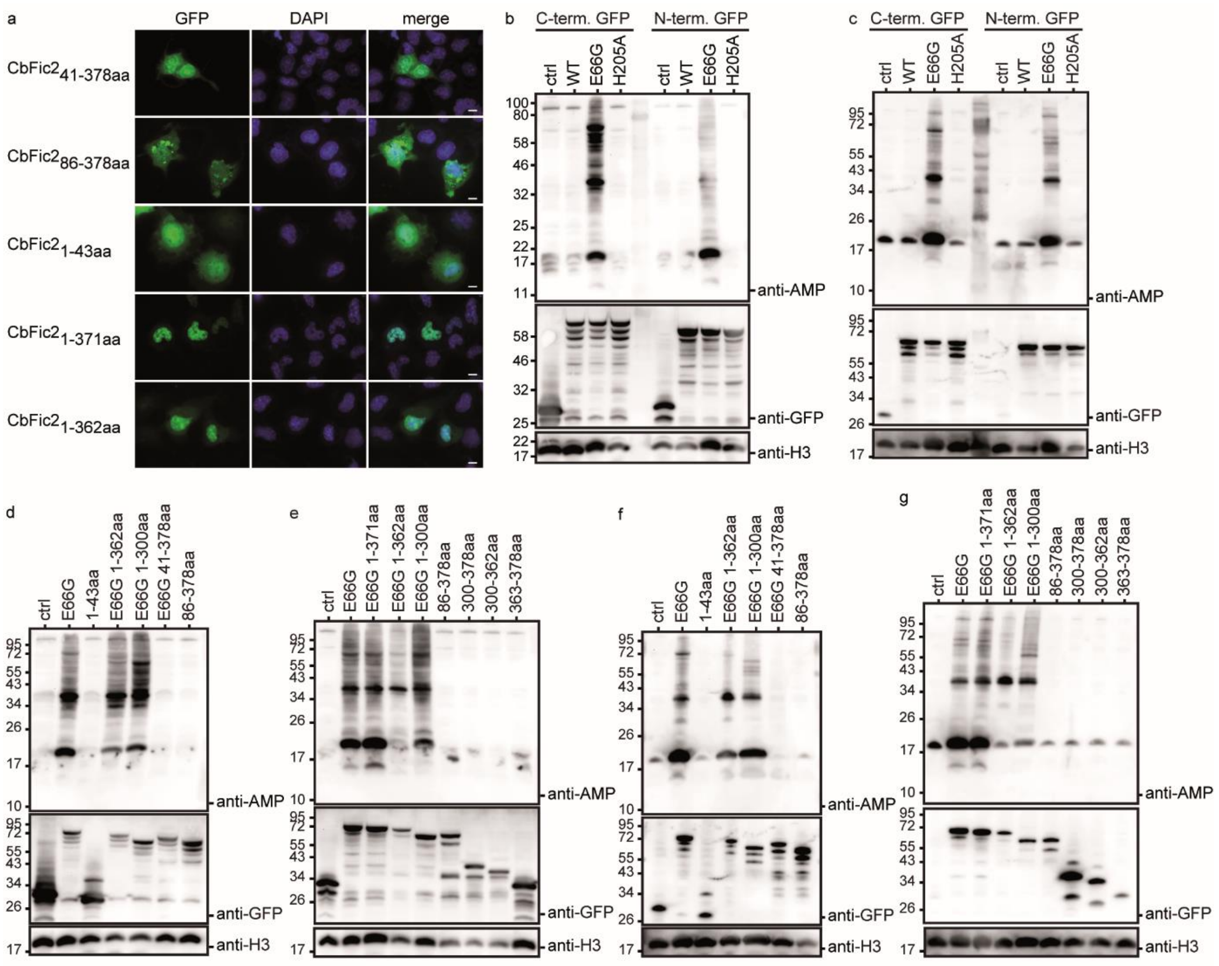


## Supplemental Figure S 1

a) Extended data of fluorescent microscopy analysis of protein localization after transient heterologous expression of indicated GFP-CbFic2 truncation constructs in Cos7 cells from Figure 1b. GFP-fusion protein (green) was expressed for 24 h and cell nuclei were stained with Hoechst-33342 (blue). Images were taken by a Leica DMi8 wide field microscope using a 100x magnification. Scale bars: 10 µm.

b) Extended data of WB analysis of AMPylation patterns of whole cell lysates after transient heterologous expression of C– or N-terminally (Figure 1c) GFP-fused CbFic2 or its mutants CbFic2E66G and CbFic2H205A in HEK293 cells. ctrl represents the expression of GFP alone. Fusion protein was expressed for 48 h in HEK293 cells. 20 µg of cleared RIPA lysate per lane were run on Bis-Tris gels and blotted on PVDF. Blots were probed with an anti-AMP antibody, stripped, cut into strips, and treated with antibodies against GFP and histone H3 as expression and loading controls, respectively.

c) Extended data of WB analysis of AMPylation patterns in histone fraction after transient heterologous expression of C– or N-terminally (Figure 1d) GFP-fused CbFic2 or its mutants CbFic2$_{E66G}$ and CbFic2$_{H205A}$ in HEK293 cells. ctrl represents the expression of GFP alone. Fusion protein was expressed for 48 h in HEK293 cells. Histones were isolated using acid extraction. 10 µg of histone extraction per lane were run on Bis-Tris gels and blotted on PVDF. Blots were probed with an anti-AMP antibody, stripped, cut into strips, and treated with antibodies against GFP and histone H3 as expression and loading controls, respectively.

d) – e) WB analysis of AMPylation patterns of whole cell lysates after transient heterologous expression of (d) CbFic2$_{E66G}$-GFP or (e) GFP-CbFic2$_{E66G}$ and truncations thereof in HEK293 cells. ctrl represents the expression of GFP alone. Fusion protein was expressed for 48 h in HEK293 cells. 20 µg of cleared RIPA lysate per lane were run on Bis-Tris gels and blotted on PVDF. Blots were probed with an anti-AMP antibody, stripped, cut into strips, and treated with antibodies against GFP and histone H3 as expression and loading controls, respectively.

f) – g) WB analysis of AMPylation patterns in histone fraction after transient heterologous expression of (f) CbFic2$_{E66G}$-GFP or (g) GFP-CbFic2$_{E66G}$ and truncations thereof in HEK293 cells. ctrl represents the expression of GFP alone. Fusion protein was expressed for 48 h in HEK293 cells. Histones were isolated using acid extraction. 10 µg of histone extraction per lane were run on Bis-Tris gels and blotted on PVDF. Blots were probed with an anti-AMP antibody, stripped, cut into strips, and treated with antibodies against GFP and histone H3 as expression and loading controls, respectively.

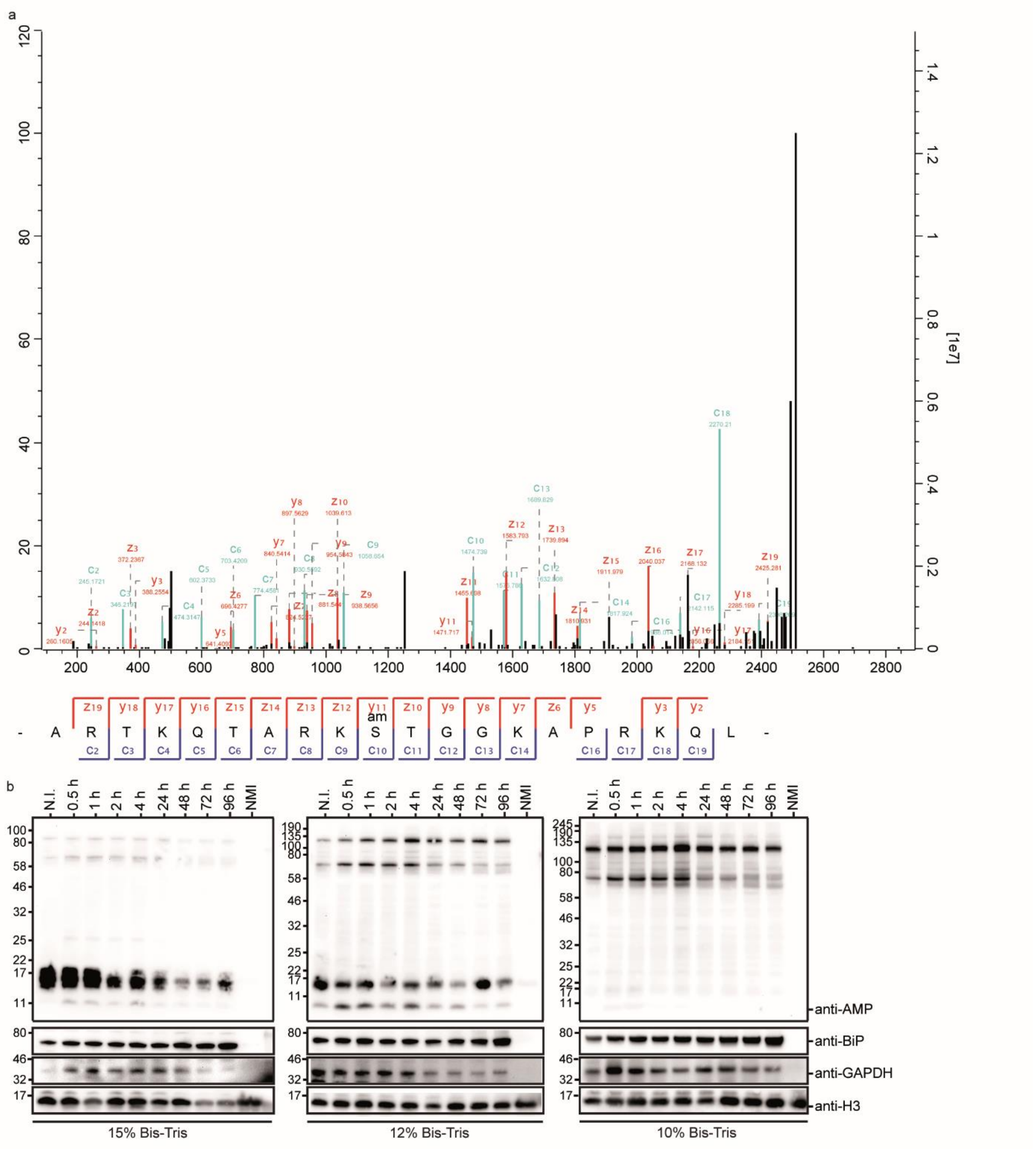


## Supplemental Figure S 2

a) MS/MS analysis of AMP modification site identification of Histone H3 by CbFic2. Histone $H3_{1\text{-}20}$ peptide was subjected to LC-MS/MS analysis after incubation with $CbFic2_{E66G}$ in the presence of ATP. The sample was run via a proteomic setup over a C18 column and eluted with increasing acetonitrile concentrations, in 0.1% formic acid. Survey scans (m/z 300-1700) were acquired in the orbitrap and most intense ions of charge states 2-7 were selected for fragmentation with high-energy collisional dissociation. Detection of single charged product ions of m/z 136.062 (adenine), 250.09 (adenosine) or 348.07 (phosphoadenosine) were triggered for refragmentation of the precursor with electron transfer dissociation (ETD). Data were acquired using Xcalibur software version 3.0sp2 (Thermo Scientific). The MS raw file was analyzed with MaxQuant software (version 1.5.3.8) (Cox and Mann 2008) and the peptide sequence ARTKQTARKSTGGKAPRKQL used for the implemented Andromeda search engine.

b) Full representation of WB analysis of AMPylation patterns over the time course of infection of murine J774 macrophages by virulent NMI *C. burnetii* (**Figure 2**i). J774 cells were infected with *C. burnetii*, and at indicated time points lysed by RIPA. 20 µg of lysate per lane was run on Bis-Tris gels (gel percentages indicated at the bottom) and blotted on PVDF. Blots were probed with an anti-AMP antibody, stripped, cut into strips, and treated with antibodies against BiP, GAPDH, and histone H3 as loading controls. N.I.: not infected, NMI: NMI cells alone.

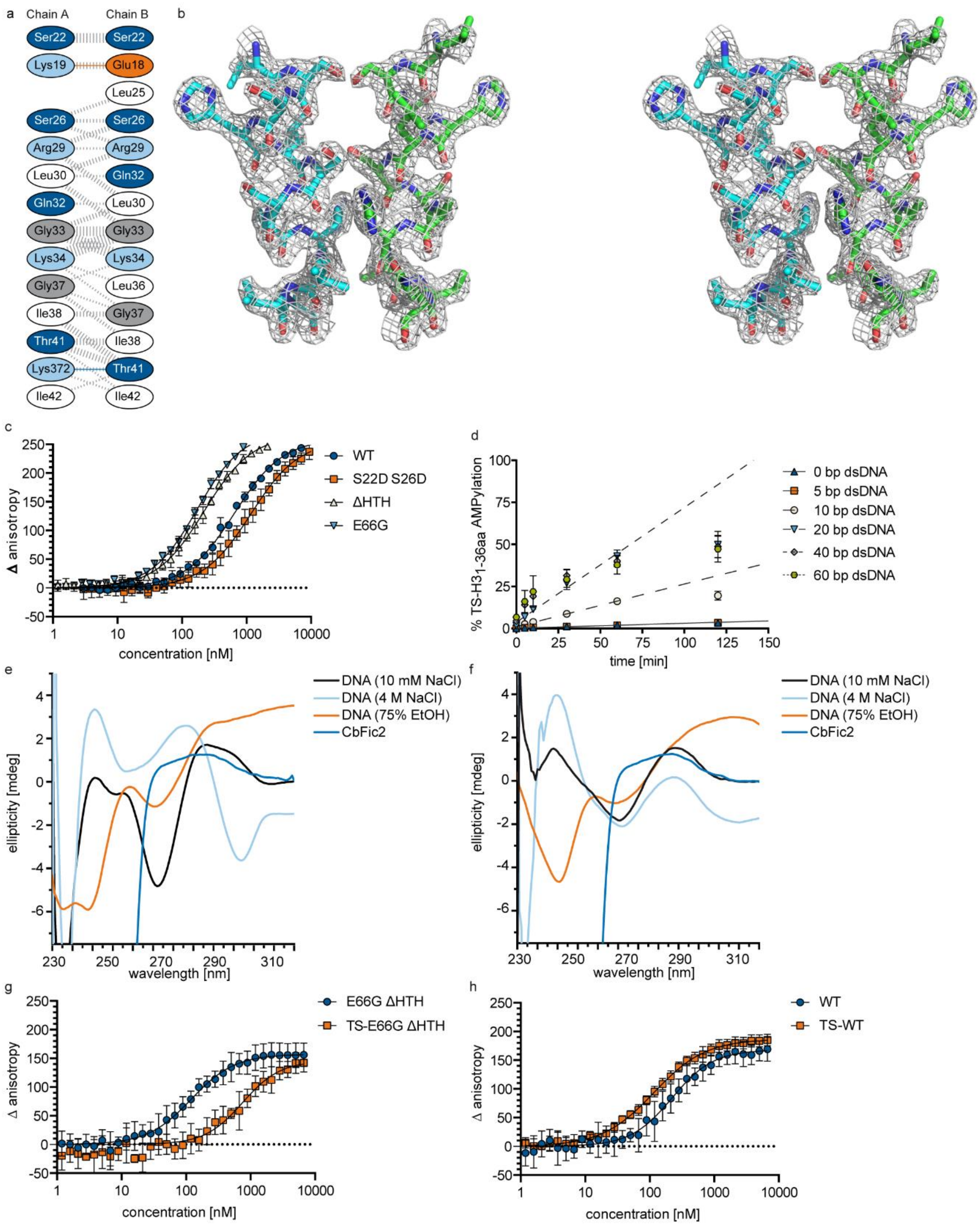


## Supplemental Figure S 3

a) Residue interactions across dimer interface of the CbFic2 crystal structure (**Figure 3**a) according to PDBsum analysis. Orange lines represent salt bridges, blue lines hydrogen bonds, grey dashed lines non-bonded contacts. For non-bonded contacts the width of the striped line is proportional to the number of atomic contacts. Positive residues (H, K, R) are colored in light blue, negative residues (D, E) in orange, neutral residues (S, T, N, Q) in dark blue, aliphatic residues (V, L, I, M) in white, proline and glycine in grey.

b) A portion of the 2mFo-Fc electron density map with a 2σ contour, in which residues 24-34 of the two CbFic protomers are represented as sticks.

c) Analysis of the binding affinity of CbFic2, CbFic2$_{E66G}$, CbFic2$_{\Delta HTH}$ and CbFic2$_{S22D\ S26D}$ against ATP by fluorescence anisotropy. 1 nM N6-(6-Aminohexyl)-ATP-5-FAM was mixed with a dilution series from 20 µM CbFic2 ($_{E66G}$, $_{\Delta HTH}$) or 50 µM CbFic2 ($_{WT}$, $_{S22D\ S26D}$) using a pipetting robot in a 384w format. Values were baseline corrected by anisotropy values of free DNA. Fit corresponds to "Specific binding with Hill slope" (3) (GraphPad Prism 8.0). Data shown correspond to the mean of technical triplicates, error bars to the standard deviation. For extended data, see **Supplemental Table 2**.

d) Linear fit to the early linear steady-state phase of the graph obtained from intact MS analysis and quantification of time-resolved TS-H3$_{1\text{-}36aa}$ AMPylation by CbFic2$_{E66G}$ in the absence or presence of 5 bp, 10 bp, 20 bp, 40 bp, 60 bp dsDNA (**Figure 3**e). 50 µM TS-H3$_{1\text{-}36aa}$ were incubated with 5 µM of CbFic2$_{E66G}$ in the presence of 5 µM DNA as indicated, 2 mM ATP, 4 mM $MgCl_2$ at 37 °C for 22 h. AMPylation was measured by the mass increase of 329 Da, and AMPylated peaks were quantified by intensity after deconvolution. AMPylation was defined as decrease in unAMPylated peptide over time, to reduce complexity of multiple AMPylation. Each data point represents the mean of biological triplicates; error bars correspond to standard deviation. Linear fit was applied to all data points between 5 min and 60 min in GraphPad Prism 8.0. For extended data, see **Supplemental Table 3**.

e) - f) CD control measurement to **Figure 3**g, h of 20 bp dsDNA with (e) 100% GC or (f) 40% content and CbFic2 alone. 1 µM of dsDNA was diluted in measurement buffer (10 mM NaCl), high salt buffer to induce Z-DNA form (4 M NaCl) or 75% Ethanol to induce A-Form (75% EtOH) of DNA, respectively. 4 µM CbFic2 alone were diluted in measurement buffer (10 mM NaCl). Before each measurement, samples were incubated for 1 h at 25 °C. CD spectra between 230 and 320 nm were collected using a 0.75 cm quartz cell.

g) - h) Analysis of the binding affinity of (g) CbFic2$_{E66G\ \Delta HTH}$ and TS-CbFic2$_{E66G\ \Delta HTH}$ or (h) CbFic2 and TS-CbFic2 against 20 bp dsDNA by fluorescence anisotropy. 1 nM 5`-FITC-labeled DNA was mixed with a dilution series from 20 µM CbFic2 using a pipetting robot in a 384w format. Values were baseline corrected by anisotropy values of free DNA. Fit corresponds to "Specific binding with Hill slope" (3) (GraphPad Prism 8.0). Data shown correspond to the mean of technical triplicates, error bars to the standard deviation. For extended data, see **Supplemental Table 2**.

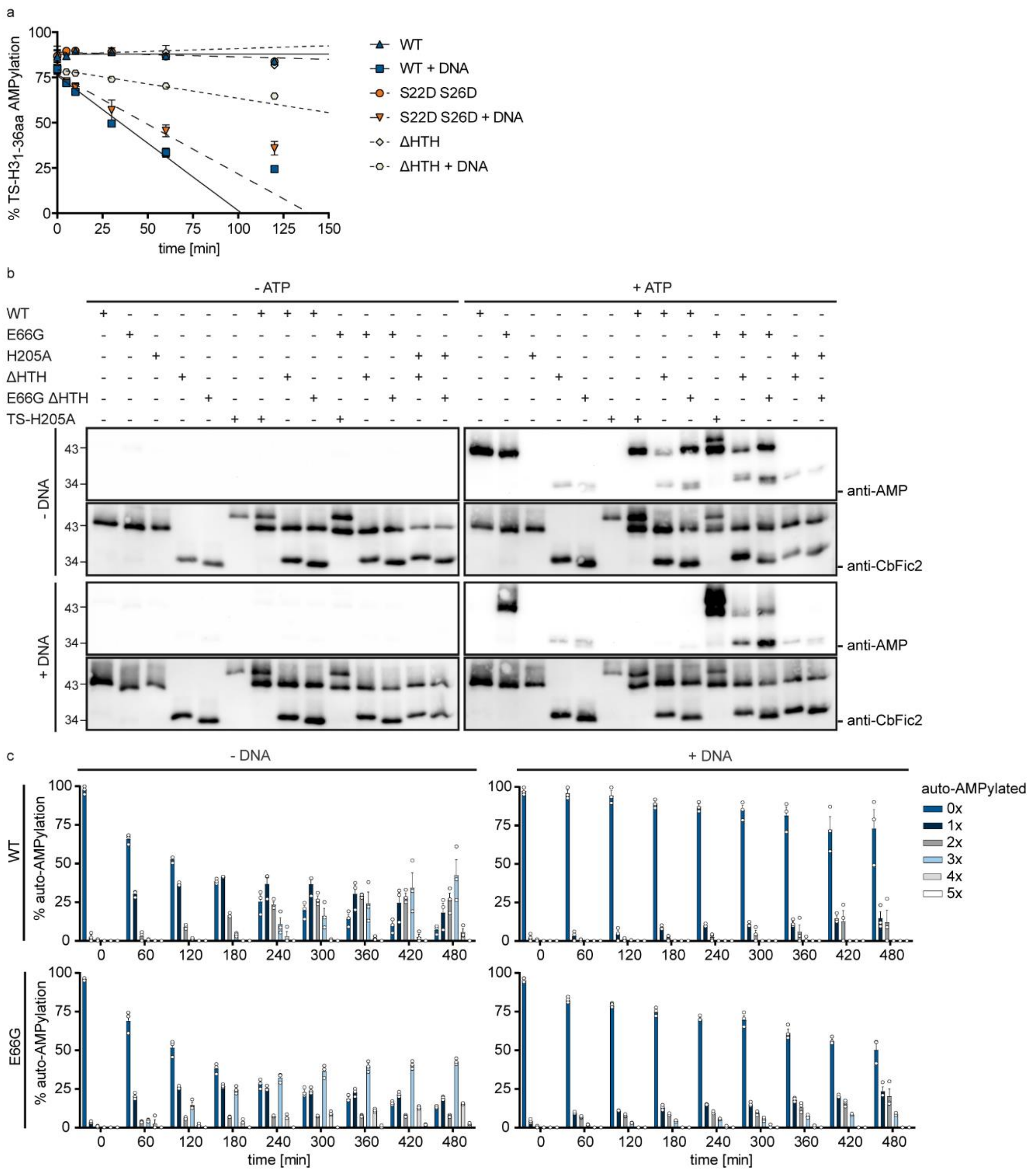


## Supplemental Figure S 4

a) Linear fit to the early linear steady-state phase of the graph obtained from intact MS analysis and quantification of time-resolved TS-H3$_{1\text{-}36aa}$-AMP deAMPylation by CbFic2, CbFic2$_{S22D\ S26D}$ or CbFic2$_{\Delta HTH}$ in the absence or presence of DNA *in vitro* (**Figure 4**b). 50 µM TS-H3$_{1\text{-}36aa}$-AMP were incubated with 0.5 µM CbFic2 as indicated in the presence of 5 µM DNA, 1 mM $MgCl_2$ at 37 °C for 24 h. DeAMPylation was measured by the mass loss of 329 Da, and peaks were quantified by intensity after deconvolution. deAMPylation was defined as increase in unAMPylated peptide over time. Each data point represents the mean of biological triplicates; error bars correspond to standard deviation. Linear fit was applied to all data points between 5 min and 60 min in GraphPad Prism 8.0. For extended data, see **Supplemental Table 3**.

b) Extended data of WB analysis of auto-AMPylation of CbFic2 in cis/trans including the control without ATP (**Figure 4**c). 0.3 µM CbFic2 versions as indicated were incubated alone or in the presence of another CbFic2 version, in the presence or absence of 2.5 µM 20 bp dsDNA, with or without 1 mM ATP, in the presence of 1 mM $MgCl_2$ for

8 h at 37 °C. 50 ng protein were run on Laemmli gels, blotted on PVDF and probed with an anti-AMP antibody. For loading controls, blots were stripped and incubated with an anti-CbFic2 antibody.

c) Full depiction of multiple auto-AMPylation from intact MS analysis of auto-AMPylation of CbFic2 and CbFic2$_{E66G}$ over the time course of incubation with ATP in the presence or absence of DNA. 0.2 mg ml$^{-1}$ (4 µM) CbFic2 or CbFic2$_{E66G}$ were incubated in 20 mM HEPES pH 7.5, 150 mM NaCl, 1 mM $MgCl_2$, 1 mM TCEP, 1 mM ATP both in the presence and absence of 5 µM 20 bp dsDNA at 37 °C and analyzed by MS. The degree of automodification was detected by the specific mass gain of AMPylation of 329 Da. AMPylation was quantified by the ratio of the specific signal intensity to the total intensity of all CbFic2 signals. As CbFic2 shows multiple auto-AMPylation, data representing the decrease of unAMPylated CbFic2 is shown in **Figure 4**d. Each bar represents the mean of biological triplicates (individual data shown as circles); error bars correspond to standard deviation.

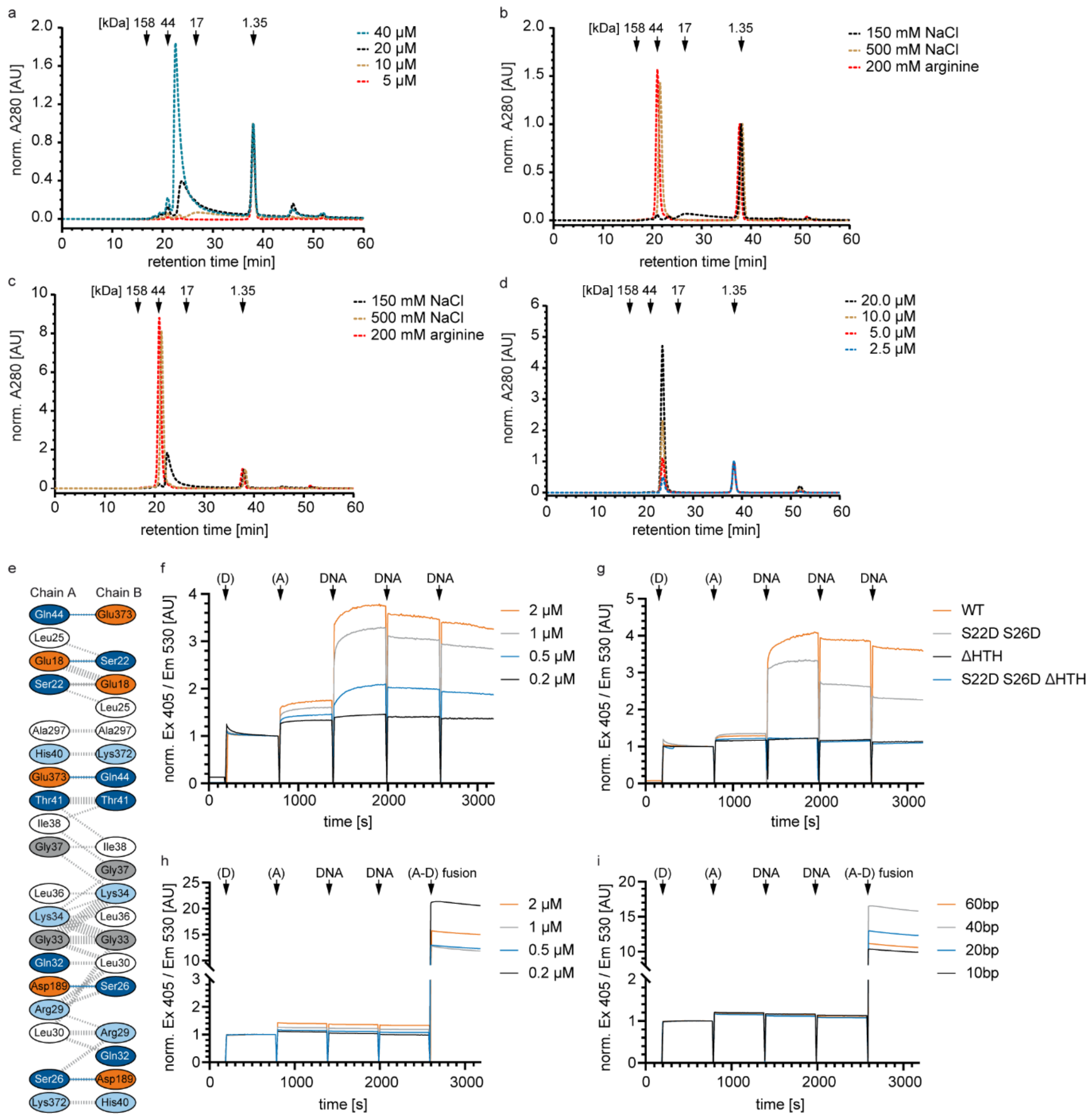


## Supplemental Figure S 5

a) Analysis of concentration dependent dimerization of CbFic2$_{E66G}$ by analytical size exclusion chromatography. CbFic2$_{E66G}$ was injected at indicated concentrations onto a Superdex 75pg 10/300 (Cytiva), run at 0.5 ml min$^{-1}$ in 20 mM HEPES pH 7.5, 150 mM NaCl, 1 mM $MgCl_2$, 1 mM TCEP, and protein was detected by absorbance at 280 nm. Data intensity was normalized to the internal control of vitamin $B_{12}$ ($t_R$ = 38.5 min). Arrows indicate the gel filtration standard (BioRad) comprising bovine γ-globulin (MW 158 kDa), chicken ovalbumin (MW 44 kDa), horse myoglobin (MW 17 kDa) and vitamin $B_{12}$ (MW 1.35 kDa).

b) –c) Secondary interaction of CbFic2 with analytical size exclusion chromatography. CbFic2 was injected at (b) 5 µM or (c) 20 µM concentration onto a Superdex 75pg 10/300 (Cytiva), run at 0.5 ml min$^{-1}$ in 20 mM HEPES pH 7.5, 150 mM NaCl, 1 mM $MgCl_2$, 1 mM TCEP (150 mM NaCl) or 20 mM HEPES pH 7.5, 500 mM NaCl, 1 mM $MgCl_2$, 1 mM TCEP (500 mM NaCl) or 20 mM HEPES pH 7.5, 150 mM NaCl, 200 mM arginine, 1 mM $MgCl_2$, 1 mM TCEP (200 mM arginine) and protein was detected by absorbance at 280 nm. Data intensity was normalized to the internal control of vitamin $B_{12}$ ($t_R$ = 38.5 min). Arrows indicate the gel filtration standard

(BioRad) comprising bovine γ-globulin (MW 158 kDa), chicken ovalbumin (MW 44 kDa), horse myoglobin (MW 17 kDa) and vitamin $B_{12}$ (MW 1.35 kDa).

d) Analysis of concentration dependent dimerization of CbFic2$_{S22D\ S26D\ \Delta HTH}$ by analytical size exclusion chromatography. CbFic2$_{S22D\ S26D\ \Delta HTH}$ was injected at indicated concentrations onto a Superdex 75pg 10/300 (Cytiva), run at 0.5 ml $min^{-1}$ in 20 mM HEPES pH 7.5, 150 mM NaCl, 1 mM $MgCl_2$, 1 mM TCEP, and protein was detected by absorbance at 280 nm. Data intensity was normalized to the internal control of vitamin $B_{12}$ ($t_R$ = 38.5 min). Arrows indicate the gel filtration standard (BioRad) comprising bovine γ-globulin (MW 158 kDa), chicken ovalbumin (MW 44 kDa), horse myoglobin (MW 17 kDa) and vitamin $B_{12}$ (MW 1.35 kDa).

e) Residue interactions across dimer interface of the AlphaFold model of CbFic2 (**Figure 5**d-f) according to PDBsum analysis. Orange lines represent salt bridges, blue lines hydrogen bonds, grey dashed lines non-bonded contacts. For non-bonded contacts the width of the striped line is proportional to the number of atomic contacts. Positive residues (H, K, R) are colored in light blue, negative residues (D, E) in orange, neutral residues (S, T, N, Q) in dark blue, aliphatic residues (V, L, I, M) in white, proline and glycine in grey.

f) – i) Analysis of DNA-induced dimerization by in-solution FP-fusion FRET time course measurements in 20 mM HEPES pH 7.5, 150 mM NaCl, 1 mM $MgCl_2$, 1 mM TCEP. After 3 min, donor CyPet-CbFic2 or its mutants (D) and after another 10 min, acceptor YPet-CbFic2 or its mutants (A) were added at concentrations of 0.2 µM, 0.5 µM, 1 µM or 2 µM as indicated (resulting in total CbFic2 concentrations of 0.4 µM, 1 µM, 2 µM or 4 µM, respectively). After another 10 min incubation, 4 µM of the indicated dsDNA was added three times in succession (DNA), with each incubation lasting 10 min. (f) represents 0.2 µM, 0.5 µM, 1 µM or 2 µM CyPet- and YPet-CbFic2 followed each by 4 µM 20 bp dsDNA, (g) 0.5 µM CyPet- and YPet-CbFic2 or CbFic2$_{S22D\ S26D}$ or CbFic2$_{\Delta HTH}$ or CbFic2$_{S22D\ S26D\ \Delta HTH}$ each followed by 4 µM 40 bp dsDNA. (h-i) For the control measurements, free CyPet and YPet were added in the order described, but DNA was added only twice, before a CyPet-YPet fusion protein was added at the same concentration as donor and acceptor alone as a positive control for maximum FRET signal. (h) represents 0.2 µM, 0.5 µM, 1 µM or 2 µM CyPet and YPet followed each by 4 µM 20 bp dsDNA (2x) and 0.2 µM, 0.5 µM, 1 µM or 2 µM CyPet-YPet fusion and (i) 0.5 µM CyPet and YPet followed by 4 µM 10 bp, 20 bp, 40 bp or 60 bp dsDNA. Measurements were performed at 25 °C, with an excitation wavelength of 405 nm and an emission wavelength of 530 nm. Intensities were normalized to the value at 760 s corresponding to the endpoint intensity of donor addition.

## Supplemental Tables:

### Supplemental Table 1: Enriched proteins in LC-MS/MS analysis after anti-AMP IP

*List of enriched proteins (Log2 FC >1.0) in LC-MS/MS analysis of anti-AMP IP from THP-1-MDMs after 48 h of CbFic2 E66G expression compared to CbFic2 H205A expression. Proteins identified exclusively in the CbFic2 E66G sample with at least 2 peptides were also included (Log2 FC n. def.).*

| Accession | Protein name | Gene name | # Peptides | Log2 FC | MW [kDa] |
|---|---|---|---|---|---|
| P98179 | RNA-binding protein 3 | RBM3 | 2 | 3.03 | 17.2 |
| P06748 | Nucleophosmin | NPM1 | 12 | 2.89 | 32.6 |
| Q14980 | Nuclear mitotic apparatus protein 1 | NUMA1 | 7 | 2.24 | 238.3 |
| P35251 | Replication factor C subunit 1 | RFC1 | 1 | 2.02 | 128.3 |
| P62995 | Transformer-2 protein homolog beta | TRA2B | 3 | 1.83 | 33.7 |
| Q8WYP5 | Protein ELYS | AHCTF1 | 3 | 1.75 | 252.5 |
| Q8IXT5 | RNA-binding protein 12B | RBM12B | 1 | 1.58 | 118.1 |
| P84243 | Histone H3.3 | H3-3A | 12 | 1.51 | 15.3 |
| Q9NYF8 | Bcl-2-associated transcription factor 1 | BCLAF1 | 1 | 1.43 | 106.1 |
| P62805 | Histone H4 | H4C1 | 10 | 1.30 | 11.4 |
| P27708 | CAD protein | CAD | 4 | 1.27 | 243.0 |
| O75367 | Core histone macro-H2A.1 | MACROH2A1 | 2 | 1.15 | 39.6 |
| Q86XP3 | ATP-dependent RNA helicase DDX42 | DDX42 | 3 | 1.15 | 103.0 |
| Q13247 | Serine/arginine-rich splicing factor 6 | SRSF6 | 6 | 1.13 | 39.6 |
| O94979 | Protein transport protein Sec31A | SEC31A | 4 | n. def. | 133.0 |
| Q13123 | Protein Red | IK | 2 | n. def. | 65.6 |
| P61163 | Alpha-centractin | ACTR1A | 2 | n. def. | 42.6 |

### Supplemental Table 2: Results of "Specific binding with Hill slope" fit of anisotropy data

*Results were obtained by fitting technical triplicates of anisotropy data with a least square fit "Specific binding with Hill slope" (3) in GraphPad Prism 8.0.*

| Protein | Ligand | $K_D$ [nM] | Hill coefficient | Figure |
|---|---|---|---|---|
| **CbFic2** | 5'-FITC 20 bp dsDNA | 220.4 | 1.354 | Figure 3i |
| **$CbFic2_{S22D\ S26D}$** | 5'-FITC 20 bp dsDNA | 333.2 | 0.934 | Figure 3i |
| **$CbFic2_{\Delta HTH}$** | 5'-FITC 20 bp dsDNA | 119.5 | 1.283 | Figure 3i |
| **CbFic2** | 5'-FITC 40 bp dsDNA | 108.5 | 0.772 | Figure 3j |
| **$CbFic2_{S22D\ S26D}$** | 5'-FITC 40 bp dsDNA | 96.0 | 0.485 | Figure 3j |
| **$CbFic2_{\Delta HTH}$** | 5'-FITC 40 bp dsDNA | 204.4 | 1.471 | Figure 3j |
| **CbFic2** | 5'-FITC 60 bp dsDNA | 36.5 | 0.988 | Figure 3k |
| **$CbFic2_{S22D\ S26D}$** | 5'-FITC 60 bp dsDNA | 63.5 | 0.921 | Figure 3k |
| **$CbFic2_{\Delta HTH}$** | 5'-FITC 60 bp dsDNA | 195.4 | 1.444 | Figure 3k |
| **$CbFic2_{E66G}$** | 5'-FITC 20 bp dsDNA | 98.6 | 1.716 | Figure 4e |
| **$CbFic2_{E66G}$-AMP** | 5'-FITC 20 bp dsDNA | 34.3 | 1.339 | Figure 4e |
| **$CbFic2_{E66G}$ + 1mM ATP** | 5'-FITC 20 bp dsDNA | 48.8 | 1.503 | Figure 4e |
| **CbFic2** | ATP-5-FAM | 568.3 | 1.256 | Supplemental Figure S 3c |
| **$CbFic2_{S22D\ S26D}$** | ATP-5-FAM | 1063.0 | 1.074 | Supplemental Figure S 3c |
| **$CbFic2_{\Delta HTH}$** | ATP-5-FAM | 259.4 | 1.078 | Supplemental Figure S 3c |
| **$CbFic2_{E66G}$** | ATP-5-FAM | 417.0 | 1.036 | Supplemental Figure S 3c |
| **TS-$CbFic2_{\Delta HTH}$** | 5'-FITC 20 bp dsDNA | 749.8 | 1.485 | Supplemental Figure S 3g |
| **TS-CbFic2** | 5'-FITC 20 bp dsDNA | 115.8 | 1.082 | Supplemental Figure S 3h |

**Supplemental Table 3: Initial rate of time-resolved AMPylation and deAMPylation by CbFic2**

*Results were obtained from the slope of a linear fit to the early linear steady-state phase of the product concentration vs reaction time graph obtained from biological triplicates in GraphPad Prism 8.0.*

| Reaction | Protein | DNA | Slope | Figure |
|---|---|---|---|---|
| **AMPylation** | $CbFic2_{E66G}$ | - | 0.03402 | Figure 3e, Supplemental Figure S 3d |
| **AMPylation** | $CbFic2_{E66G}$ | 5 bp dsDNA | 0.02868 | Figure 3e, Supplemental Figure S 3d |
| **AMPylation** | $CbFic2_{E66G}$ | 10 bp dsDNA | 0.2535 | Figure 3e, Supplemental Figure S 3d |
| **AMPylation** | $CbFic2_{E66G}$ | 20 bp dsDNA | 0.6819 | Figure 3e, Supplemental Figure S 3d |
| **deAMPylation** | CbFic2 | - | 0.0001873 | Figure 4b, Supplemental Figure S 4a |
| **deAMPylation** | CbFic2 | 20 bp dsDNA | -0.7454 | Figure 4b, Supplemental Figure S 4a |
| **deAMPylation** | $CbFic2_{S22D\ S26D}$ | - | -0.02597 | Figure 4b, Supplemental Figure S 4a |
| **deAMPylation** | $CbFic2_{S22D\ S26D}$ | 20 bp dsDNA | -0.5486 | Figure 4b, Supplemental Figure S 4a |
| **deAMPylation** | $CbFic2_{\Delta HTH}$ | - | 0.03145 | Figure 4b, Supplemental Figure S 4a |
| **deAMPylation** | $CbFic2_{\Delta HTH}$ | 20 bp dsDNA | -0.1587 | Figure 4b, Supplemental Figure S 4a |

**Supplemental Table 4: DNA oligonucleotides for use as co-substrates for CbFic2 (IDT)**

*Extinction coefficients $\varepsilon_{(260\ nm)}$ and molecular masses (MW) were calculated using the IDT Oligo Analyzer (IDT)*

| length | GC-content | sequence fw | sequence rv |
|---|---|---|---|
| 5 bp | 40% | CATGT | ACATG |
| 10 bp | 40% | TACGATGTAC | GTACATCGTA |
| 20 bp | 40% | AGTTTAGTGCGCTAATCTGA | TCAGATTAGCGCACTAAACT |
| 40 bp | 40% | TTGATTACCGTCAGTTGGAGCCCAT ATTATACATGAAGAC | GTCTTCATGTATAATATGGGCTC CAACTGACGGTAATCAA |
| 60 bp | 40% | TCACTCAAGAAGCAGACACAGTAA GACACGGTTTAGCTGATTGTTTATC GATTAGGTCAA | TTGACCTAATCGATAAACAATCA GCTAAACCGTGTCTTACTGTGTC TGCTTCTTGAGTGA |

**Supplemental Table 5: Oligonucleotides for fluorescence anisotropy**

*Extinction coefficients $\varepsilon_{(260\ nm)}$ and molecular masses (MW) were calculated using the IDT Oligo Analyzer (IDT), using 5'-6FAM as a label in the absence of 5'-FITC.*

| oligonucleotide | MW [Da] | $\varepsilon_{(260\ nm)}$ [$M^{-1}\ cm^{-1}$] | provider |
|---|---|---|---|
| 5‘-FITC-20bp_40%GC_fw | 6 715 | 217 760 | Eurofins, Luxemburg |
| 5‘-FITC-40bp_40%GC_fw | 12 836 | 416 060 | Eurofins |
| 5‘-FITC-60bp_40%GC_fw | 19 074 | 622 360 | Eurofins |
| 20bp_40%GC_rv | 6 085 | 195 600 | IDT |
| 40bp_40%GC_rv | 12 294 | 394 100 | IDT |
| 60bp_40%GC_rv | 18 414 | 573 400 | IDT |
