# Supplementary material for: The DNA-binding induced (de)AMPylation activity of a Coxiella burnetii Fic enzyme targets Histone H3
Source: Commun Biol. 2023 Nov 6;6:1124. doi: 10.1038/s42003-023-05494-7 (PMC10628234; doi:10.1038/s42003-023-05494-7)
Supplement: Supplementary file 2 — Description of Additional Supplementary Files [file 42003_2023_5494_MOESM2_ESM.pdf]

Description of Additional Supplementary Files

File name: Supplementary Data

Description: The source data behind graphs and modelled structures, as well as all raw images behind Western Blot and gel depictions.
