# Supplementary material for: The DNA-binding induced (de)AMPylation activity of a Coxiella burnetii Fic enzyme targets Histone H3
Source: Commun Biol. 2023 Nov 6;6:1124. doi: 10.1038/s42003-023-05494-7 (PMC10628234; doi:10.1038/s42003-023-05494-7)
Supplement: Supplementary file 4 — Reporting Summary [file 42003_2023_5494_MOESM4_ESM.pdf]

nature portfolio

Corresponding author(s): Aymelt Itzen

Last updated by author(s): Oct 19, 2023

# Reporting Summary

Nature Portfolio wishes to improve the reproducibility of the work that we publish. This form provides structure for consistency and transparency in reporting. For further information on Nature Portfolio policies, see our Editorial Policies and the Editorial Policy Checklist.

## Statistics

For all statistical analyses, confirm that the following items are present in the figure legend, table legend, main text, or Methods section.

| n/a | Confirmed | |
|---|---|---|
| ☐ | ☒ | The exact sample size (*n*) for each experimental group/condition, given as a discrete number and unit of measurement |
| ☐ | ☒ | A statement on whether measurements were taken from distinct samples or whether the same sample was measured repeatedly |
| ☐ | ☒ | The statistical test(s) used AND whether they are one- or two-sided<br>*Only common tests should be described solely by name; describe more complex techniques in the Methods section.* |
| ☒ | ☐ | A description of all covariates tested |
| ☒ | ☐ | A description of any assumptions or corrections, such as tests of normality and adjustment for multiple comparisons |
| ☐ | ☒ | A full description of the statistical parameters including central tendency (e.g. means) or other basic estimates (e.g. regression coefficient) AND variation (e.g. standard deviation) or associated estimates of uncertainty (e.g. confidence intervals) |
| ☐ | ☒ | For null hypothesis testing, the test statistic (e.g. *F*, *t*, *r*) with confidence intervals, effect sizes, degrees of freedom and *P* value noted<br>*Give P values as exact values whenever suitable.* |
| ☒ | ☐ | For Bayesian analysis, information on the choice of priors and Markov chain Monte Carlo settings |
| ☒ | ☐ | For hierarchical and complex designs, identification of the appropriate level for tests and full reporting of outcomes |
| ☒ | ☐ | Estimates of effect sizes (e.g. Cohen's *d*, Pearson's *r*), indicating how they were calculated |

*Our web collection on statistics for biologists contains articles on many of the points above.*

## Software and code

Policy information about availability of computer code

| | |
|---|---|
| Data collection | Microscopy images were acquired with LAS X 2 software (Leica microsystems). WBs and gel images were acquired with ChemoStar Touch v0.5.65 (Intas Science Imaging). Anisotropy data was collected with a Spark plate reader using SparkControl V2.3 software (Tecan). TSA data was acquired with the Mx3000P Real Time PCR Cycler (Agilent Technologies). Intact MS with amaZon speed ESI-LCMS or maXis II ETD ESI-qTOF LC-MS were acquired using Compass OpenAccess 1.4 software (Bruker Daltonik). Data of LC-MS/MS identification of AMP modification site were acquired using Xcalibur software version 3.0sp2 (Thermo Scientific). CbFic2 protein structure and dimer prediction were generated by ColabFold: AlphaFold2 using MMseqs2 (Jumper et al. 2021; Mirdita et al. 2022a; 2022b). CD spectra were collected with Chirascan Spectrometer Control Panel Application v4.7.0.194. FRET data were collected with the Spectra Time Course Measurement within the Spectra Manager Version 2.15.01 (JASCO Corporation). aSEC data were collected with Prominence HPLC system (Shimadzu, Kyōto, Japan). |
| Data analysis | aSEC data, anisotropy data and TSA data was analyzed and fitted with GraphPad Prism 8.0. All intact MS data were analyzed with DataAnalysis 5.1 (Bruker Daltonics). MS raw file of LC-MS/MS identification of AMP modification site was analyzed with MaxQuant software (version 1.5.3.8). LC-MS/MS data after anti-AMP IP were searched with the Sequest algorithm integrated in the Proteome Discoverer software (v 2.41.15, Thermo Fisher Scientific) against a reviewed human Swissprot database, obtained in April 2020, containing 20365 entries. Structural data were visualized with The PyMOL Molecular Graphics System, Version 2.3.2 Schrödinger, LLC. CD spectra were analyzed via Pro-Data Viewer v4.2. Crystallography data were analyzed with XDS v2021, AIMLESS v0.7.4, AutoRickshaw v1.08, MoRDa v1.5.07, Buccaneer v1.5, REFMAC5 v5.8, PHENIX v1.19 and COOT v0.9.8. FRET data were smoothed by the means-movement method using a convolution width of 11 with Spectra Analysis Version 2.15.18 within the Spectra Manager Version 2.15.01 (JASCO Corporation). |

For manuscripts utilizing custom algorithms or software that are central to the research but not yet described in published literature, software must be made available to editors and reviewers. We strongly encourage code deposition in a community repository (e.g. GitHub). See the Nature Portfolio guidelines for submitting code & software for further information.

## Data

Policy information about availability of data

All manuscripts must include a data availability statement. This statement should provide the following information, where applicable:

- Accession codes, unique identifiers, or web links for publicly available datasets
- A description of any restrictions on data availability
- For clinical datasets or third party data, please ensure that the statement adheres to our policy

Structure factors and model coordinates have been deposited in the RCSB PDB under the accession code 8CIL. All generated AlphaFold models are provided as supplement.
The mass spectrometry proteomics data have been deposited to the ProteomeXchange Consortium (Deutsch et al. 2020) via the PRIDE (Perez-Riverol et al. 2019) partner repository with the dataset identifier PXD040330. During review, the data can be accessed via a Reviewer account:
Username: reviewer_pxd040330@ebi.ac.uk
Password: Mua7hwkV

## Human research participants

Policy information about studies involving human research participants and Sex and Gender in Research.

| | |
|---|---|
| Reporting on sex and gender | n/a |
| Population characteristics | n/a |
| Recruitment | n/a |
| Ethics oversight | n/a |

Note that full information on the approval of the study protocol must also be provided in the manuscript.

# Field-specific reporting

Please select the one below that is the best fit for your research. If you are not sure, read the appropriate sections before making your selection.

☒ Life sciences ☐ Behavioural & social sciences ☐ Ecological, evolutionary & environmental sciences

For a reference copy of the document with all sections, see nature.com/documents/nr-reporting-summary-flat.pdf

# Life sciences study design

All studies must disclose on these points even when the disclosure is negative.

| | |
|---|---|
| Sample size | Statistical predetermination of sample size was not performed. For intact MS in vitro assays, three independent biological replicates were performed to ensure reproducibility of the findings (n=3). Anti-AMP IP for LC-MS/MS analysis was performed in three independent biological replicates (n=3). Anisotropy, CD, and TSA measurements were performed in technical triplicates and were not used for statistical evaluation. All WB analyses based on in vitro assays, THP-1 cells and HEK293 cells were performed in biological triplicates to ensure reproducibility, representative blots are shown. WB analysis of Coxiella infection was performed in technical triplicates based on one biological replicate, representative blots are shown. Sample sizes were chosen by following the rule of providing three independant biological or technical replicates, depending on the experiment as mentioned in the manuscript for all individual experiments. Analytical size exclusion chromatography was performed as biological duplicates; representative chromatograms are shown. At least technical duplicates were produced and representative data is shown for time resolved FP-FRET. Co-IP experiments of HA- and V5-tagged CbFic2 were performed as technical duplicates, representative blots are shown. |
| Data exclusions | No data were excluded. |
| Replication | All data presented in the manuscript are reproducible. |
| Randomization | n/a |
| Blinding | n/a |

# Reporting for specific materials, systems and methods

We require information from authors about some types of materials, experimental systems and methods used in many studies. Here, indicate whether each material, system or method listed is relevant to your study. If you are not sure if a list item applies to your research, read the appropriate section before selecting a response.

## Materials & experimental systems

| n/a | Involved in the study |
|---|---|
| ☐ | ☒ Antibodies |
| ☐ | ☒ Eukaryotic cell lines |
| ☒ | ☐ Palaeontology and archaeology |
| ☐ | ☒ Animals and other organisms |
| ☒ | ☐ Clinical data |
| ☒ | ☐ Dual use research of concern |

## Methods

| n/a | Involved in the study |
|---|---|
| ☒ | ☐ ChIP-seq |
| ☒ | ☐ Flow cytometry |
| ☒ | ☐ MRI-based neuroimaging |

# Antibodies

Antibodies used

For IF, anti-AMP antibody 17G6 (Höpfner et al. 2020, Lot. A218101379) was used. For IP, anti-histone H3 antibody ab1791 (abcam, Lot. GR322435-1), anti-myc antibody 9E10 sc-40 (Santa Cruz Biotechnology, Lot. C1119), anti-HA Tag Monoclonal Antibody (2-2.2.14) (Thermo Fisher Scientific, Lot.YB382604) and anti-AMP antibody 17G6 (Höpfner et al. 2020, Lot. A218101379) were used. For WB, mouse anti-AMP monoclonal antibody 17G6 (Höpfner et al. 2020, Lot. A218101379), mouse anti-GAPDH monoclonal antibody 0411 sc-47724 (Santa Cruz Biotechnology, Lot. C01117), rabbit polyclonal anti histone H3 antibody ab1791 (abcam, Lot. GR322435-1), polyclonal rabbit anti-GRP78/BiP antibody PA5-34941 (Thermo Fisher Scientific, Lot. VE2993689C and UA2704091A), chicken anti-CbFic2 antibody (custom production by AG Kaspers, LMU, Munich, Germany), rabbit anti-GFP Polyclonal Antibody A111-22 (Thermo Fisher Scientific, Lot. 2477546), mouse anti-V5 Tag Monoclonal Antibody (TCM5), eBioscience™ 14-6796-82 (Thermo Fisher Scientific, Lot. 2093237), HisProbe™-HRP-conjugate 15165 (Thermo Fisher Scientific, Lot. UL293993), secondary goat anti-mouse IgG (H + L) HRP conjugate 31430 (Thermo Fisher Scientific, Lot. TL272497), secondary goat anti-rabbit IgG H&L (HRP) preadsorbed ab7090 (abcam, Lot. GR340710-4), secondary rabbit anti-chicken IgY (IgG) (H+L) HRP conjugate 303-035-003 (Jackson ImmunoResearch Laboratories, Lot. 135423), anti-HA Tag Monoclonal Antibody (2-2.2.14) (Thermo Fisher Scientific, Lot.YB382604) were used.

Validation

anti-histone H3 antibody is suitable for IP and WB, reacts with: Mouse, Human (source: abcam)
anti-myc antibody is recommended for detection of c-Myc tagged fusion proteins of mouse, human origin by WB and IP (source: Santa Cruz Biotechnology).
anti-AMP antibody is validated for IP and WB and reacts with AMPylated proteins and ADP ribosylated proteins independent of backbone (Höpfner et al. 2020).
anti-GAPDH antibody is recommended for detection of GAPDH of human origin by Western Blotting (source: Santa Cruz Biotechnology).
anti-GRP78/BiP antibody is suitable for WB and reacts with Human and Mouse Hspa5 aa 594 - 655 (source Thermo Fisher Scientific).
anti-CbFic2 antibody was validated in WB for recognition of recombinant CbFic2 and CbFic2 wo HTH domain and no recognition of Histone H3 (In-house).
anti-GFP antibody is suitable for WB and reacts with GFP (Aequorea victoria) tags (source Thermo Fisher Scientific).
anti-V5 antibody is suitable for WB and reacts with the tag sequence -Gly-Lys-Pro-Ile-Pro-Asn-Pro-Leu-Leu-Gly-Leu-Asp-Ser-Thr- (source Thermo Fisher Scientific).
HisProbe™-HRP-conjugate is suitable for WB and reacts with His-tags (source Thermo Fisher Scientific).
anti-HA Tag Monoclonal Antibody (2-2.2.14) is suitable for WB and IP and reacts with the HA peptide YPYDVPDYA derivitized to ovalbumin (source Thermo Fisher Scientific)

# Eukaryotic cell lines

Policy information about cell lines and Sex and Gender in Research

Cell line source(s)

HEK293 (DSMZ ACC-305)
Cos7 cells (Sigma)
HEK293-T cells (Prof. Manfred Jücker, Institute of Biochemistry and Signal Transduction, University Medical Center Hamburg-Eppendorf)
THP-1 cells (ATCC TIB-202)
J774 and L929 cells (Soraya Mezouar)

Authentication

HEK293 (DSMZ ACC-305), THP-1 cells (ATCC TIB-202) and Cos7 cells (Sigma) were freshly purchased from supplier and not further verified. L929, J774 cells and HEK293-T cells were not futher verified.

Mycoplasma contamination

All cell lines tested negative for mycoplasm contamination

Commonly misidentified lines
(See ICLAC register)

*Name any commonly misidentified cell lines used in the study and provide a rationale for their use.*

## Animals and other research organisms

Policy information about studies involving animals; ARRIVE guidelines recommended for reporting animal research, and Sex and Gender in Research

| | |
|---|---|
| Laboratory animals | Escherichia coli (strains: Mach1, Lemo21(DE3), Rosetta 2), Coxiella burnetii Nine Mile I (NMI) strain |
| Wild animals | n/a |
| Reporting on sex | n/a |
| Field-collected samples | n/a |
| Ethics oversight | n/a |

Note that full information on the approval of the study protocol must also be provided in the manuscript.
